# Supplementary material for: Mitofusin 2 displays fusion-independent roles in proteostasis surveillance
Source: Nat Commun. 2025 Feb 10;16:1501. doi: 10.1038/s41467-025-56673-5 (PMC11811173; doi:10.1038/s41467-025-56673-5)
Supplement: Supplementary file 1 — Supplementary Information [file 41467_2025_56673_MOESM1_ESM.pdf]

Supplementary information

Mitofusin 2 displays fusion-independent roles in proteostasis surveillance

Mariana Joaquim[1,2,3#], Selver Altin[1,2#], Maria-Bianca Bulimaga[1,2,3,4], Tânia Simões[1,2] Hendrik Nolte[1,2$], Verian Bader[5], Camilla Aurora Franchino[2,3,6], Solenn Plouzennec[7], Karolina Szczepanowska[2,8], Elena Marchesan[9], Kay Hofmann[1], Marcus Krüger[1,2,3], Elena Ziviani[9], Aleksandra Trifunovic[2,3], Arnaud Chevrollier[7], Konstanze F. Winklhofer[5], Elisa Motori[2,3,6], Margarete Odenthal[3,4], Mafalda Escobar-Henriques[1,2,3*]

[#] Contributed equally to this work

* Corresponding author
E-mail: Mafalda.Escobar@uni-koeln.de
Mailing address: Institute for Genetics, Zülpicher Str. 47a, 50674 Cologne, Germany
Phone: +49 221 470 89053

[1]Institute for Genetics, University of Cologne, Germany, [2]Cologne Excellence Cluster on Cellular Stress Responses in Aging-Associated Diseases (CECAD), University of Cologne, Germany, [3]Center for Molecular Medicine Cologne (CMMC), University of Cologne, Germany, [4]Institute of Pathology, Medical Faculty of the University of Cologne and University Hospital of Cologne, Germany, [5]Department Molecular Cell Biology, Institute of Biochemistry and Pathobiochemistry, Ruhr University Bochum, Germany, and Cluster of Excellence RESOLV, Bochum, Germany, [6]Institute for Biochemistry, University of Cologne, Germany, [7]University of Angers, MitoLab Team, MitoVasc Unit, CNRS UMR6015, INSERM U1083, SFR ICAT, Angers, France, [8]ReMedy International Research Agenda Unit, International Institute of Molecular Mechanisms and Machines (IMol), Polish Academy of Sciences, 00-783 Warsaw, Poland, [9]Deparment of Biology, University of Padova, Italy.

[$] current address: MPI for Biology of Ageing, 50931 Cologne, Germany

Supplementary Figures

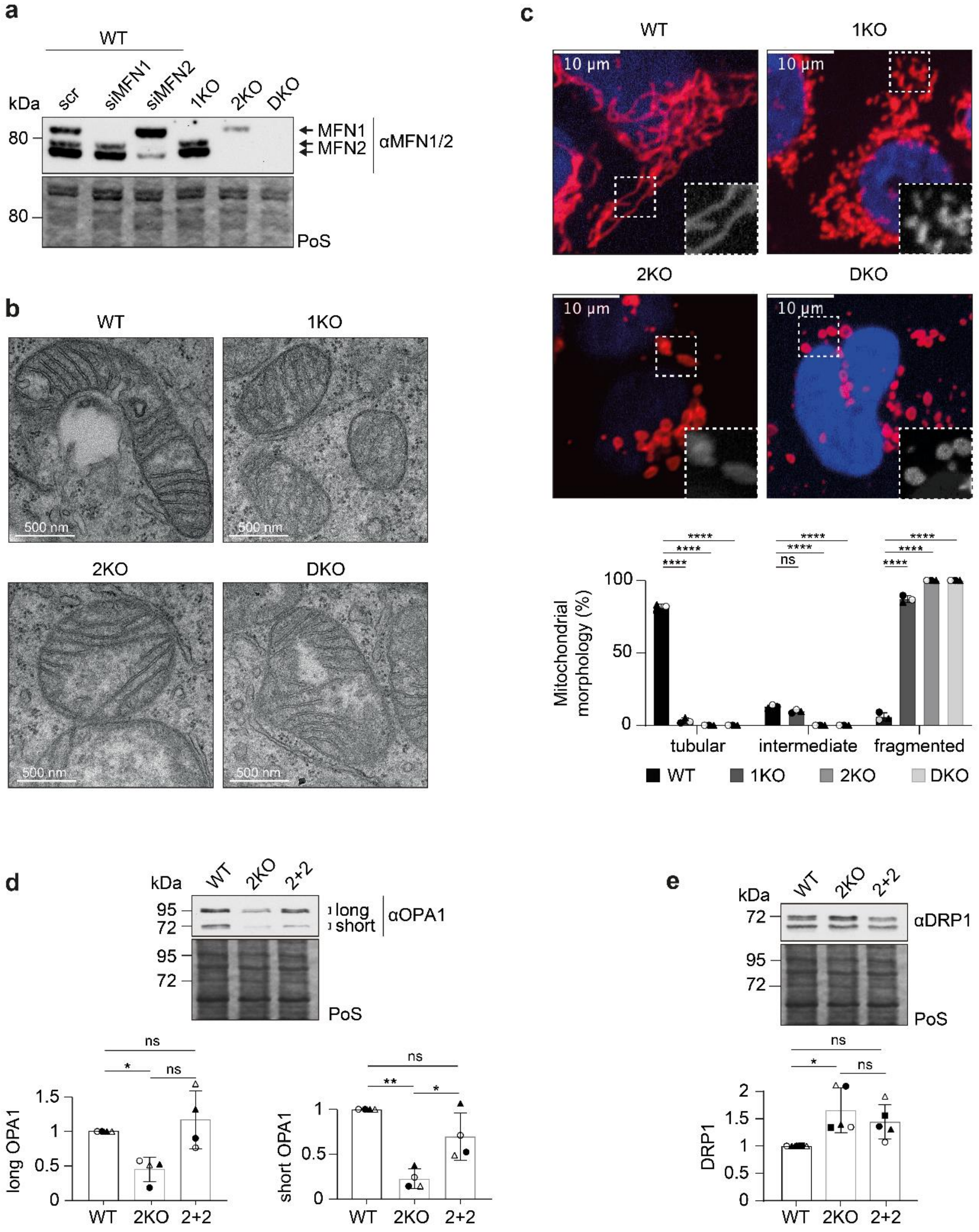

Supplementary Fig. 1: Mitochondrial fragmentation upon loss of MFN1 or MFN2 in HEK cells. **(a)** Western blot analysis of total cell lysates from HEK WT, 1KO, 2KO or double MFN1/2 knockout (DKO) cells, immunoblotted with anti-MFN1/2. WT cells were transfected with either a scramble siRNA (scr), MFN1 siRNA (siMFN1) or MFN2 siRNA (siMFN2). Staining of total protein with PoS was used as loading control. Two-way ANOVA was applied. **(b)** Electron microscopy images of

mitochondria in HEK WT, 1KO, 2KO and DKO cells. Scale bar: 5µm. **(c)** Confocal images and mitochondrial morphology quantification, after immunostaining of the outer mitochondrial membrane protein TOM20 (in red) and nucleus staining with DAPI (in blue) of HEK WT, 1KO, 2KO and DKO cells. Scale bar: 10µm. Zoomed insets are shown within each image. Mitochondrial morphology was categorized in tubular, intermediate and fragmented. Percentage of cells showing the respective mitochondrial phenotypes was quantified. At least 100 cells per condition were counted. Bars represent the average fold change relative to WT ± SD (n=3 biological replicates). Individual values for each experiment are discriminated in black triangles and white and black filled circles. **(d)** Western blot analysis (upper panel) and quantification (lower panels) of total cell lysates from HEK WT, 2KO and 2+2 cells, immunoblotted with anti-OPA1. Staining of total protein with PoS was used as loading control. RM one-way ANOVA was applied. **(e)** Western blot analysis (upper panel) and quantification (lower panel) of total cell lysates from HEK WT, 2KO and 2+2 cells, immunoblotted with anti-DRP1. Staining of total protein with PoS was used as loading control. RM one-way ANOVA was applied. Source data and exact p values are provided as a Source Data file.

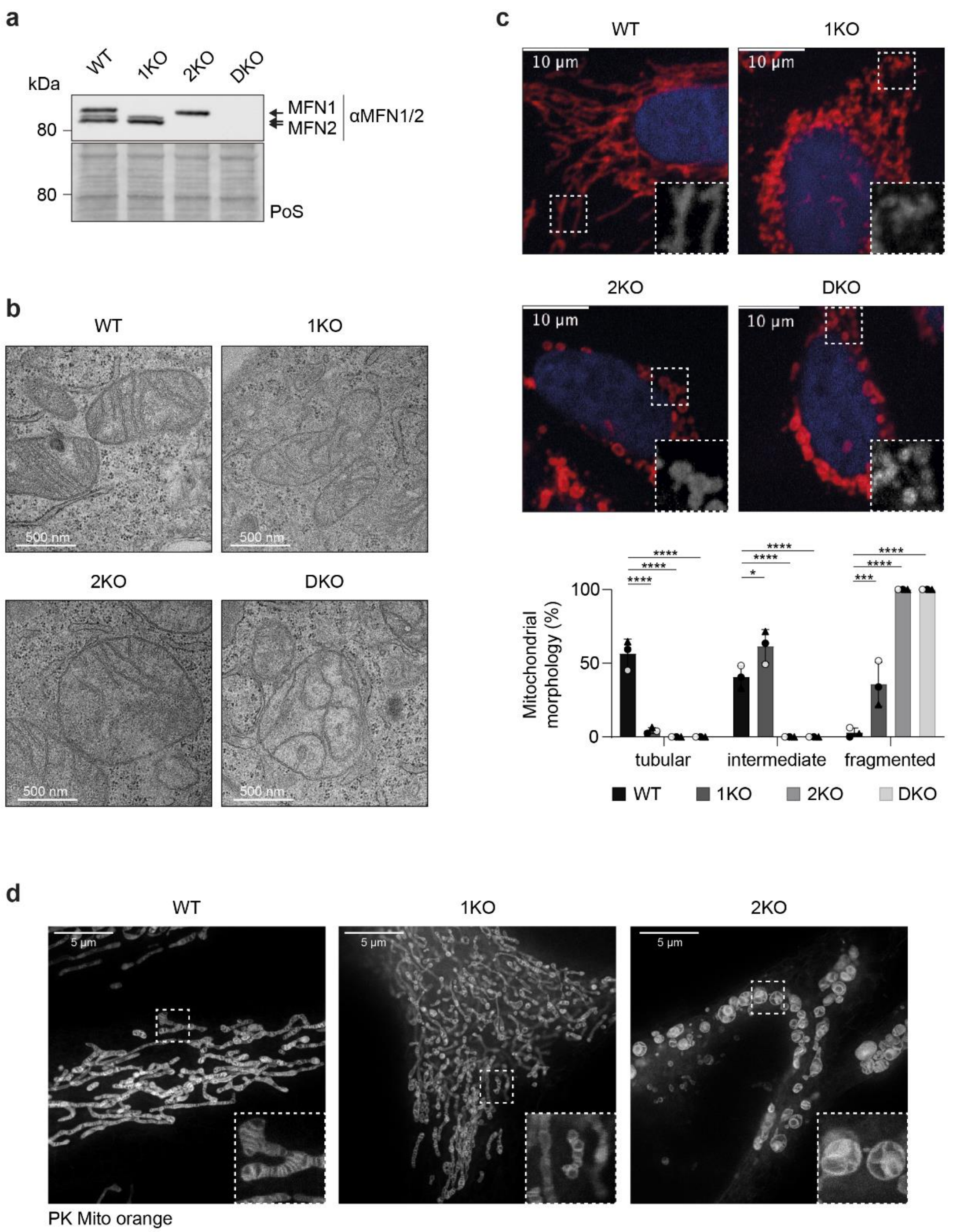

Supplementary Fig. 2: Mitochondrial fragmentation upon loss of MFN1 or MFN2 in HeLa cells. **(a)** Western blot analysis of total cell lysates from HeLa WT, 1KO, 2KO or double MFN1/2 knockout (DKO), immunoblotted with anti-MFN1/2. Staining of total protein with PoS was used as loading control. **(b)** Electron microscopy images of mitochondria in HeLa WT, 1KO, 2KO and DKO cells. Scale bar: 5μm. **(c)** Confocal images and mitochondrial morphology quantification, after immunostaining of the outer mitochondrial membrane protein TOM20 (in red) and nucleus staining with DAPI (in blue) of HeLa WT, 1KO, 2KO and DKO cells. Scale bar: 10μm. Zoomed insets are shown within each image. Mitochondrial morphology was categorized in tubular,

intermediate and fragmented. Percentage of cells showing the respective mitochondrial phenotypes was quantified. One biological replicate with at least 100 cells per condition as quantified. Bars represent the average fold change relative to WT ± SD (n=3 biological replicates). Individual values for each experiment are discriminated in black triangles and white and black filled circles. Two-way ANOVA was applied. **(d)** Confocal images after live PK Mito Orange staining of Hela WT, 1KO and 2KO cells. Scale bar: 5µm. Zoomed images corresponding to the dotted white boxes are shown within each image. Source data and exact p values are provided as a Source Data file.

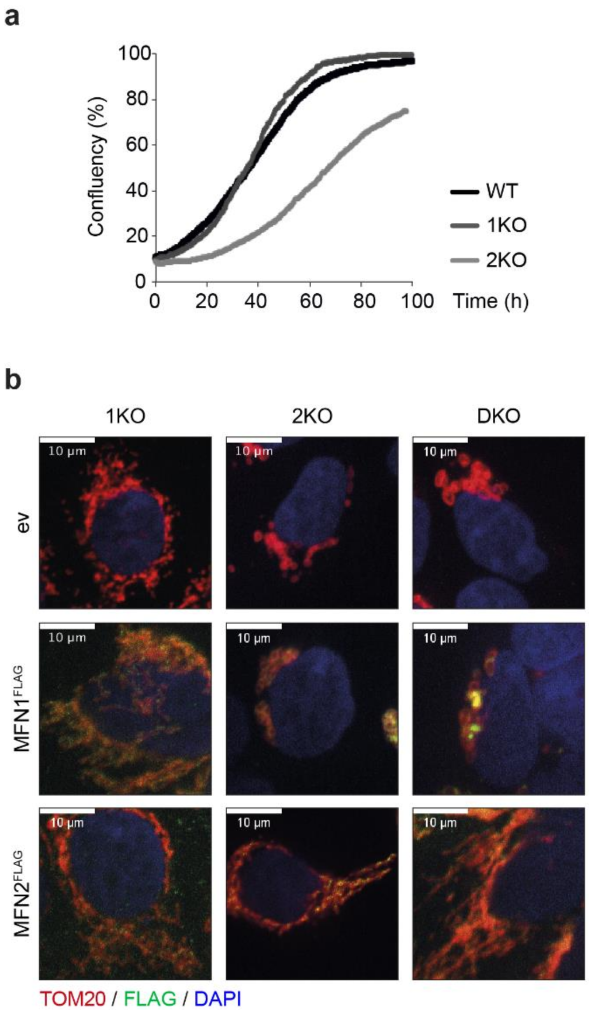

Supplementary Fig. 3: MFN2 is sufficient to promote mitochondrial fusion in single and double KOs. **(a)** Cellular growth curve measured by cell confluency (in percentage) of HEK WT, 1KO and 2KO cells, over the course of 4 days. **(b)** Confocal images after immunostaining of TOM20 (in red) and FLAG (in green) and nuclear staining with DAPI (in blue) of HEK 1KO, 2KO and DKO cells, transfected with either empty vector (ev), MFN1^{FLAG} or MFN2^{FLAG}. Scale bar: 10µm. Source data are provided as a Source Data file.

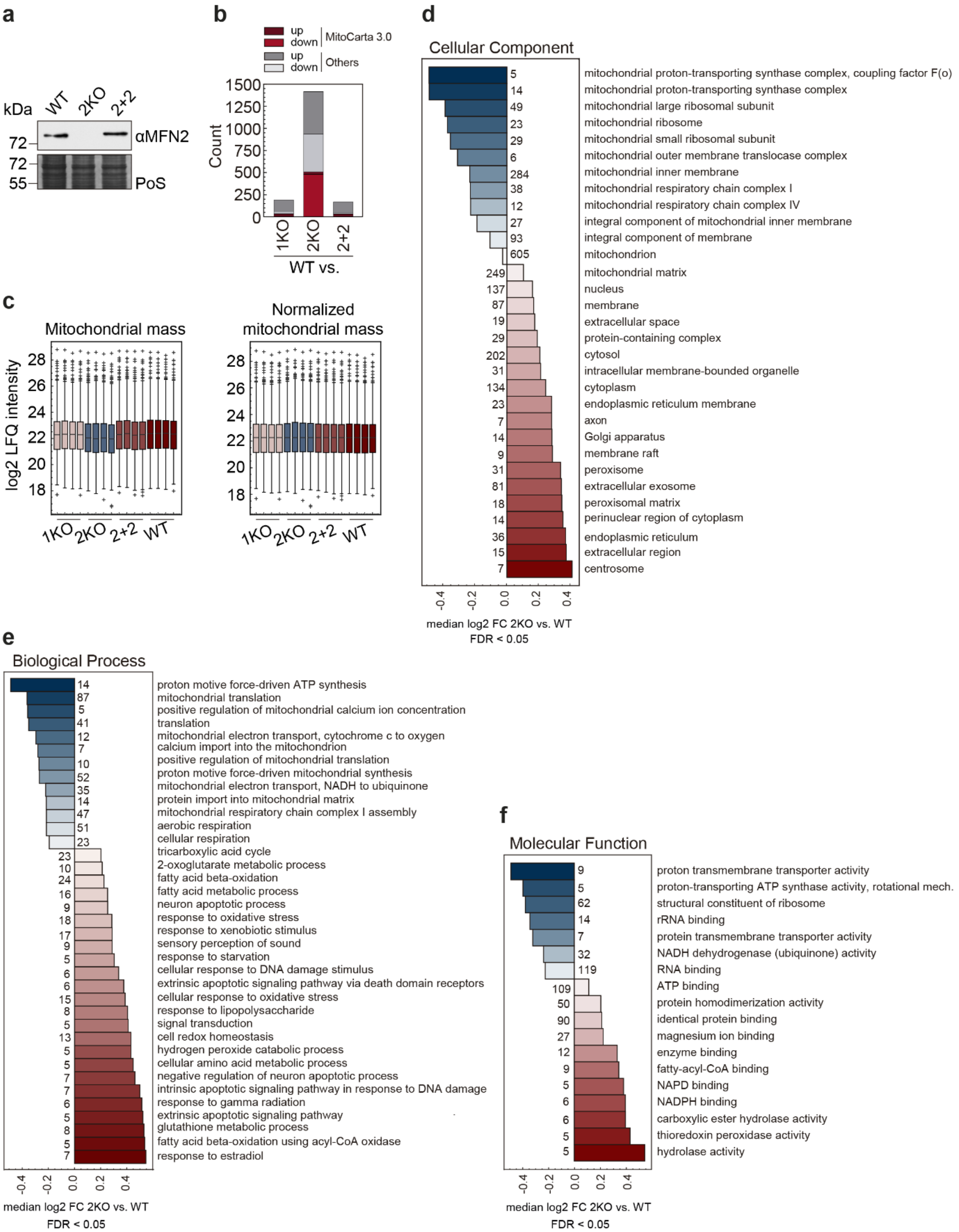

Supplementary Fig. 4: Cellular pathways differentially regulated in 2KO cells. **(a)** Western blot analysis of total cell lysates from HEK WT, 2KO and 2+2 cells, immunoblotted with anti-MFN2. Staining of total protein with PoS was used as loading control. **(b)** Bar graph showing the number

of significantly upregulated and downregulated protein groups, belonging to MitoCarta3.0 (red) or others (grey), between 1KO, 2KO or 2+2 versus WT cells (n=4 biological replicates), based on an unpaired t-test followed by a permutation-based FDR (5%). **(c)** Boxplot analysis of raw (left) and normalized (right) log2 LFQ intensities of mitochondrial proteins (MitoCarta 3.0) in 1KO, 2KO, 2+2 or WT cells (n=4 biological replicates). The normalization for mitochondrial mass was done by shifting the median of each sample to the global median of all samples. **(d-f)** 1D Enrichment analysis showing the median log2 fold change (all protein groups annotated by the given pathway) of 2KO versus WT cells (n=4 biological replicates) using the gene ontology biological process annotations **(on d)**, the gene ontology cellular component **(on e)** and the gene ontology molecular function **(on f)**. The number of proteins identified in each pathway are annotated next to respective bar. FDR < 0.02. Source data and exact p values are provided as a Source Data file.

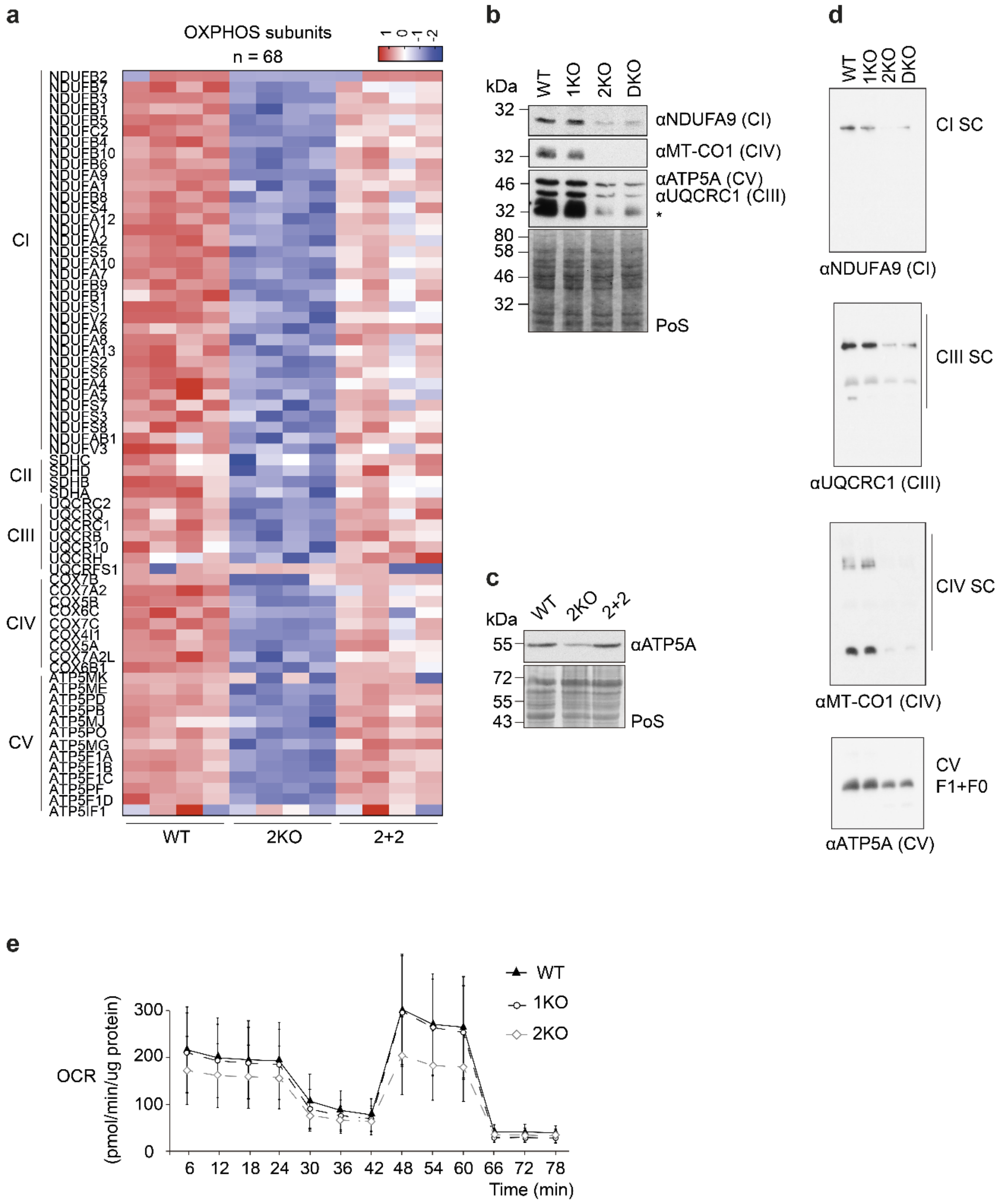

Supplementary Fig. 5: MFN2 is required for OXPHOS function. **(a)** Hierarchical clustering of log2 transformed LFQ intensities, Z-Score normalized, of OXPHOS subunits proteins, in HEK WT, 2KO and 2+2 cells (n=4 biological replicates). **(b)** Western blot analysis of total cell lysates from HEK WT, 1KO, 2KO and DKO cells, immunoblotted with anti-NDUFA9, anti-MT-CO1, anti-ATP5α and anti-UQCRC1. In the third panel, (*) indicates a previous signal from anti-MT-CO1.

Staining of total protein with PoS was used as loading control. **(c)** Western blot analysis of crude mitochondrial lysates from HEK WT, 2KO and 2+2 cells, immunoblotted with anti-ATP5α. Staining of total protein with PoS was used as loading control. **(d)** Blue Native-PAGE of mitochondrial crude extracts from HEK WT, 1KO, 2KO and DKO cells, followed by Western blot analysis of the mitochondrial respiratory supercomplexes, immunoblotted with anti-NDUFA9, anti-UQCRC1, anti-MT-CO1 and anti-ATP5α. **(e)** Oxygen consumption rate measurement of HEK WT, 1KO and 2KO cells for 78 min. At minute 24, 42 and 66, oligomycin, CCCP and antimycin A/rotenone, previously loaded into the ports, were added to the cells, respectively. The points (triangle, circle and diamond) represent the average fold change ± SD (n=4 biological replicates). Source data and exact p values are provided as a Source Data file.

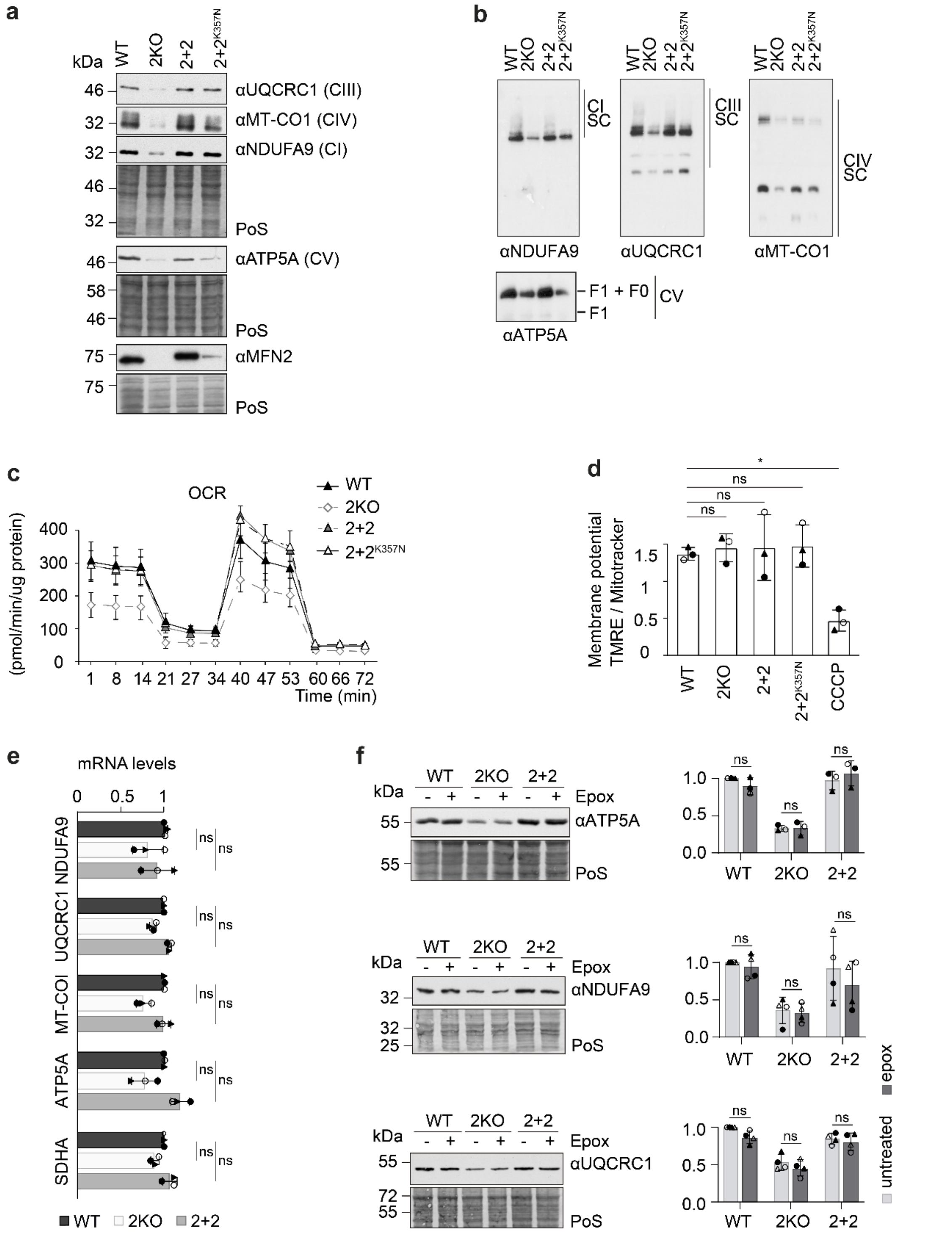

Supplementary Fig. 6: Rescue of OXPHOS defects by exogenous expression of MFN2. **(a)** Western blot analysis of total cell lysates from HEK WT, 2KO, 2+2 and 2+2^{K357N} cells, immunoblotted with anti-UQCRC1, anti-MT-CO1, anti-NDUFA9, anti-ATP5α and anti-MFN2. Staining of total protein with PoS was used as loading control. **(b)** Blue Native-PAGE of mitochondrial crude extracts from HEK WT, 2KO, 2+2 and 2+2^{K357N} cells, followed by Western blot analysis of the mitochondrial respiratory supercomplexes, immunoblotted with anti-NDUFA9,

anti-UQCRC1, anti-MT-CO1 and anti-ATP5α. **(c)** Oxygen consumption rate measurement of HEK WT, 2KO, 2+2 and 2+2^{K357N} cells for 72 min. At minute 21, 40 and 53, oligomycin, CCCP and antimycin A/rotenone, previously loaded into the ports, were added to the cells, respectively. The points (triangles and diamond) represent the average fold change ± SD (n=5 biological replicates). **(d)** Mitochondrial membrane potential was measured as a ratio between TMRE intensity and Mitotracker Deep Red intensity in HEK WT untreated or treated with CCCP (20µM, 2h), 2KO, 2+2 and 2+2^{K357N} cells. Individual values of each experiment are discriminated in triangles and while or blacked filled circles. Bars represent means ± SD (n=3 biological replicates). RM one-way ANOVA was applied. **(e)** Relative mRNA levels of NDUFA9, UQCRC1, MT-COI, ATP5α and SDHA, measured by qPCR of total mRNA extracted from HEK WT, 2KO and 2+2 cells. Bars represent the average of transcript levels ± SD (n=3 biological replicates). Individual values of each experiment are discriminated in triangles and white or black filled circles. RM one-way ANOVA was applied. **(f)** Western blot analysis (left panels) and quantification (right panels) of total cell lysates from HEK WT, 2KO, 2+2 cells, untreated (-) or treated (+) with epoxomicin (1µM, 2h), immunoblotted with anti-ATP5α (upper panel), anti-NDUFA9 (middle panel) or anti-UQCRC1 (lower panel). Staining of total protein with PoS was used as loading control**.** Bars represent the average fold change relative to WT ± SD (n=4 biological replicates). Individual values of each experiment are discriminated in white or black filled circles and triangles. Two-way ANOVA was applied. Source data and exact p values are provided as a Source Data file.

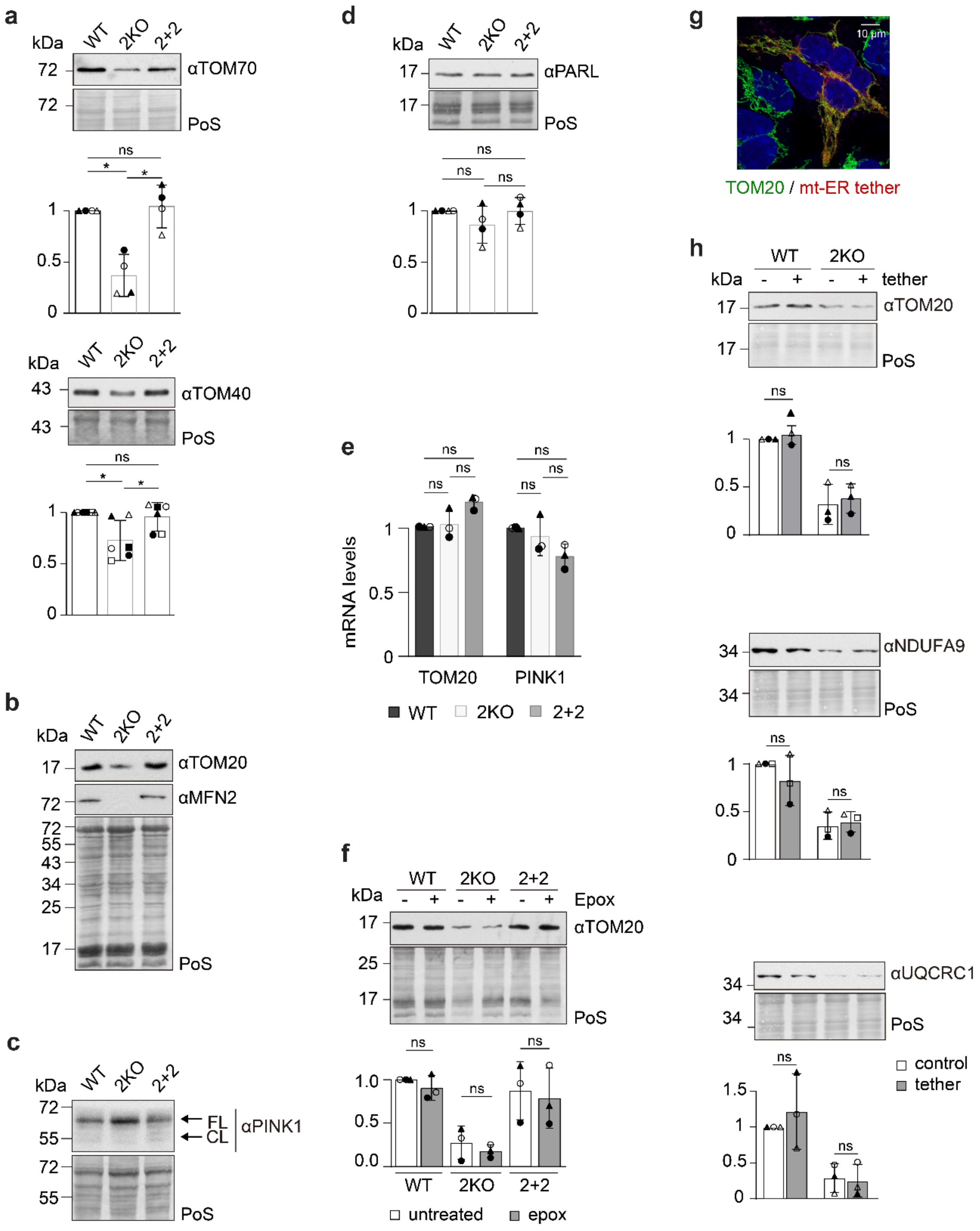

Supplementary Fig. 7: MFN2 loss affects import machinery levels. **(a)** Western blot analysis (upper panel) and quantification (lower panel) of total cell lysates from HEK WT, 2KO and 2+2 cells, immunoblotted with anti-TOM70 (first two panels) and anti-TOM40 (second two panels). Staining of total protein with PoS was used as loading control**.** Bars represent the average fold change relative to WT ± SD (n=4 biological replicates for TOM70 and n=6 biological replicates for TOM40). Individual values for each experiment are discriminated in white and black filled circles, triangles and squares. RM one-way ANOVA was applied. **(b)** Western blot analysis of crude

mitochondrial lysates from HEK WT, 2KO and 2+2 cells, immunoblotted with anti-TOM20 and anti-MFN2. Staining of total protein with PoS was used as loading control. **(c)** Western blot analysis of crude mitochondrial lysates from HEK WT, 2KO and 2+2 cells, immunoblotted with anti-PINK1. Staining of total protein with PoS was used as loading control. **(d)** Western blot analysis (upper panel) and quantification (lower panel) of total cell lysates from HEK WT, 2KO and 2+2 cells, immunoblotted with anti-PARL. Staining of total protein with PoS was used as loading control. Bars represent the average fold change relative to WT ± SD (n=4 biological replicates). Individual values for each experiment are discriminated in white or black filled circles and triangles. RM one-way ANOVA was applied. **(e)** Relative mRNA levels of TOM20 and PINK1 measured by qPCR of total mRNA extracted from HEK WT, 2KO and 2+2 cells. Bars represent the average of transcript levels ± SD (n=3 biological replicates). Individual values of each experiment are discriminated in triangles and white or black filled circles. RM one-way ANOVA was applied. **(f)** Western blot analysis (upper panel) and quantification (lower panel) of total cell lysates from HEK WT and 2KO cells, untreated (-) or treated (+) with epoxomicin (1µM, 2h), immunoblotted with anti-TOM20. Staining of total protein with PoS was used as loading control. Bars represent the average fold change relative to WT ± SD (n=3 biological replicates). Individual values for each experiment are discriminated in triangles and white or black filled circles. **(g)** Confocal image after immunostaining of TOM20 (in green), RFP (in red) and nuclear staining with DAPI (in blue) of HEK WT cells transfected with the artificial mito-ER tether AKAP1-mRFP-UBC6. Scale bar: 10µm. Two-way ANOVA was applied. **(h)** Western blot analysis (upper panel) and quantification (lower panel) of total cell lysates from HEK WT and 2KO cells, transfected with the control vector (-) or with the mito-ER tether AKAP1-mRFP-UBC6 (+), immunoblotted with anti-TOM20 (upper panel), anti-NDUFA9 (middle panel) or anti-UQCRC1 (lower panel). Staining of total protein with PoS was used as loading control. Bars represent the average fold change relative to WT ± SD (n=3 biological replicates). Individual values for each experiment are discriminated in white or black filled circles, triangles and squares. Two-way ANOVA was applied. Source data and exact p values are provided as a Source Data file.

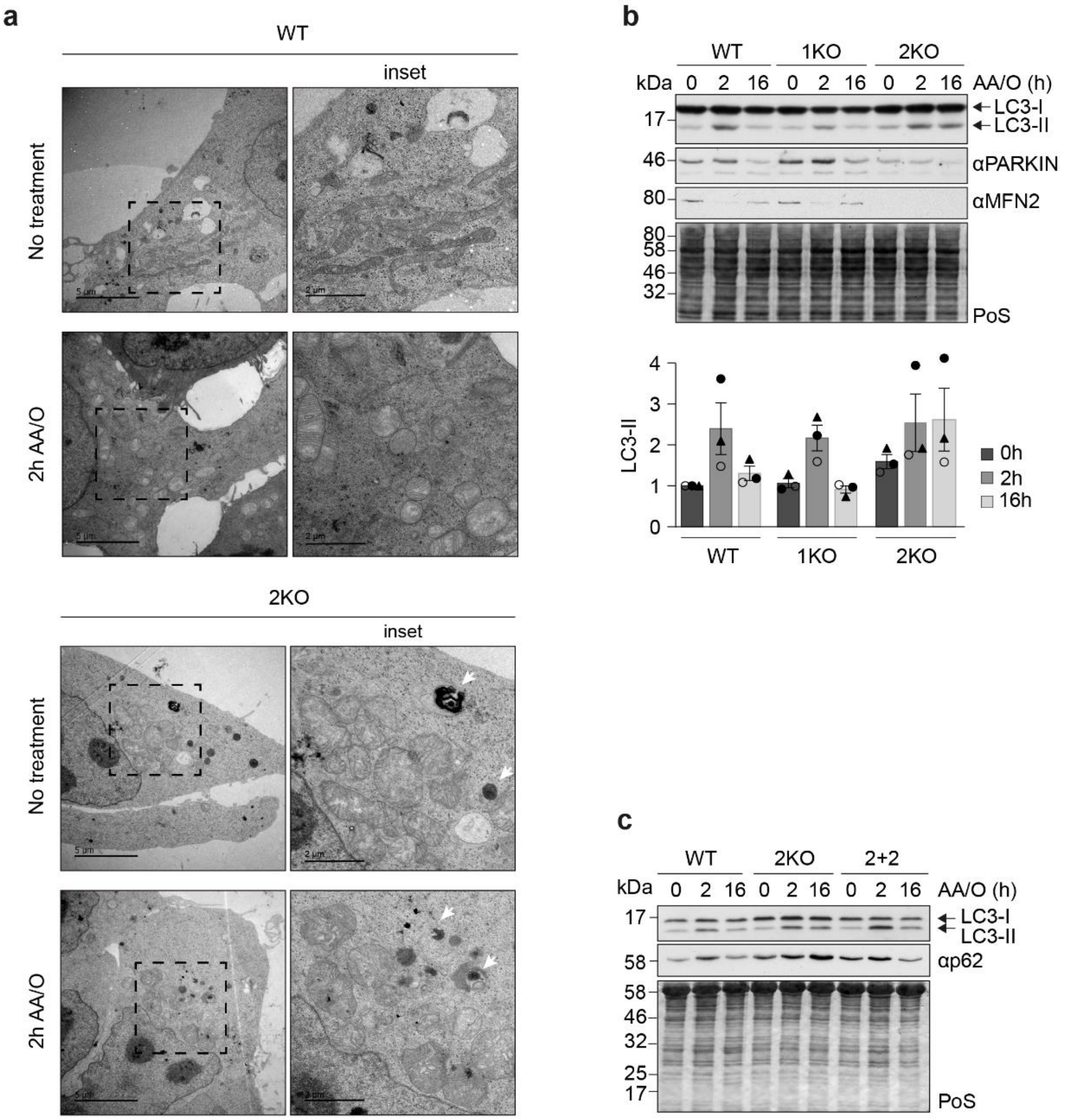

Supplementary Fig. 8: 2KO cells exhibit increased mitophagy. **(a)** Electron microscopy images of HEK WT and 2KO cells, untreated or treated with antimycin A (1µM) and oligomycin (10µM) (AA/O) for 2h. Zoomed insets are shown on the right side of each image. Scale bar: 5µm (left) and 2µm (right). **(b)** Western blot analysis (upper panel) and quantification (lower panel) of total cell lysates from HEK WT, 1KO and 2KO cells untreated (0) or treated with antimycin A (1µM) and oligomycin (10µM) (AA/O) for 2 or 16h, immunoblotted with anti-LC3 I/II, anti-PARKIN and anti-MFN2. Staining of total protein with PoS was used as loading control. Bars represent the average fold change relative to WT ± SD (n=3 biological replicates). The individual values for each experiment are discriminated in triangles and white or black filled circles. **(c)** Western blot analysis of crude mitochondrial fractions of HEK WT, 2KO and 2+2 cells untreated (0) or treated with antimycin A (1µM) and oligomycin (10µM) (AA/O) for 2 or 16h, immunoblotted with anti-LC3 I/II and anti-p62. Staining of total protein with PoS was used as loading control. Source data and exact p values are provided as a Source Data file.

a

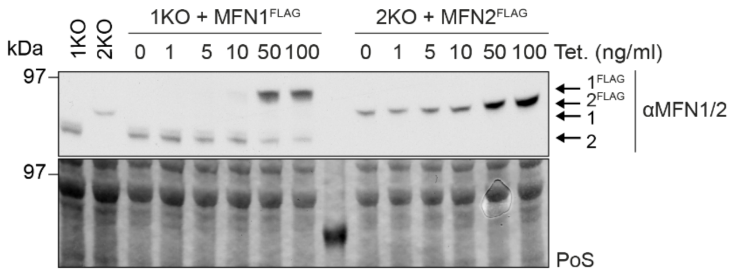

b

MFN2 interactome

	Proteins	Enrichment MFN2	Enrichment MFN1
	MFN2	10.90	4.81
Proteasomal subunits	A1	4.31	2.80
	A3	4.79	1.14
	A4	2.40	1.50
	A5	3.02	2.16
	A6	3.63	2.08
	A7;A8	5.15	2.06
	B1	3.47	1.32
	B2	2.65	2.19
	C2	2.97	1.60
	C3	4.17	2.13
	C4	4.06	2.15
	C5	4.23	1.76
	C6	3.45	0.48
	D1	4.07	1.20
	D2	2.18	1.29
	D3	2.67	1.03
	D4	3.76	2.38
	D6	3.31	0.87
	D13	1.84	1.85
QC proteins	p97/VCP	3.56	2.20
	HSP90	2.22	2.29
	HSC70	1.06	0.89
	STIP1	4.33	3.55
	BAG2	3.67	2.56
	UBL4A	1.41	0.19
	PPIL4	2.10	-0.32
	NAP1L1	1.06	-0.17
E3 lig.	MARCH5	3.39	0.94

c

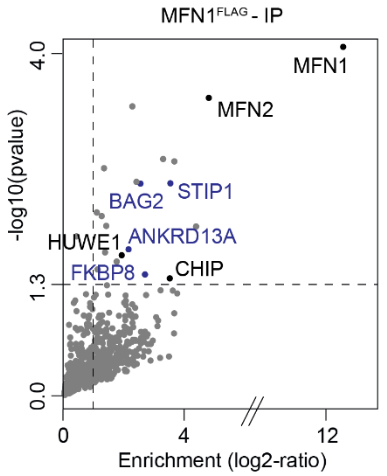

MFN1 interactome

	Proteins	Enrichment MFN1	Enrichment MFN2
	MFN1	12.57	2.67
	MFN2	4.81	10.90
QC proteins	STIP1	3.55	4.33
	FKBP8	2.71	1.53
	BAG2	2.56	3.67
	ANKRD13A	2.17	-0.68
E3 lig.	CHIP	3.53	4.14
	HUWE1	1.94	1.08

Supplementary Fig. 9: MFN1 and MFN2 do not overlap in their interactome. **(a)** Western blot analysis of total cell lysates from HEK 1KO and 2KO cells stably expressing tetracycline inducible MFN1^{FLAG} and MFN2^{FLAG}, respectively, after a tetracycline titration (0-100 ng/mL), immunoblotted with anti-MFN1/2. The arrows point to the bands corresponding to MFN2, MFN1, MFN2^{FLAG} and MFN1^{FLAG}. **(b)** Comparison of enrichment (log2 ratio) of the proteins significantly enriched in the MFN2-FLAG immunoprecipitated interactome and respective enrichment in the MFN1-FLAG immunoprecipitated interactome. (n=3 biological replicates). Non-significant enrichment values (p-value < -log10 1.3) are displayed in grey and significant (p-value ≥ -log10 1.3) enrichment values are displayed in black. **(c)** Upper panel: Volcano plot of significantly enriched proteins (p=0,05, 2-fold enrichment) identified by label-free quantification of immunoprecipitated MFN1^{FLAG}. Proteins of interest are highlighted in colors: E3 ligases (in black) and quality control-related proteins (in blue). Lower panel: Comparison of enrichment (log2 ratio) of the proteins

significantly enriched in the MFN1-FLAG immunoprecipitated interactome and respective enrichment in the MFN2-FLAG immunoprecipitated interactome. (n=3 biological replicates). Non-significant enrichment values (p-value < -log10 1.3) are displayed in grey and significant (p-value ≥ -log10 1.3) enrichment values are displayed in black. Source data and exact p values are provided as a Source Data file.

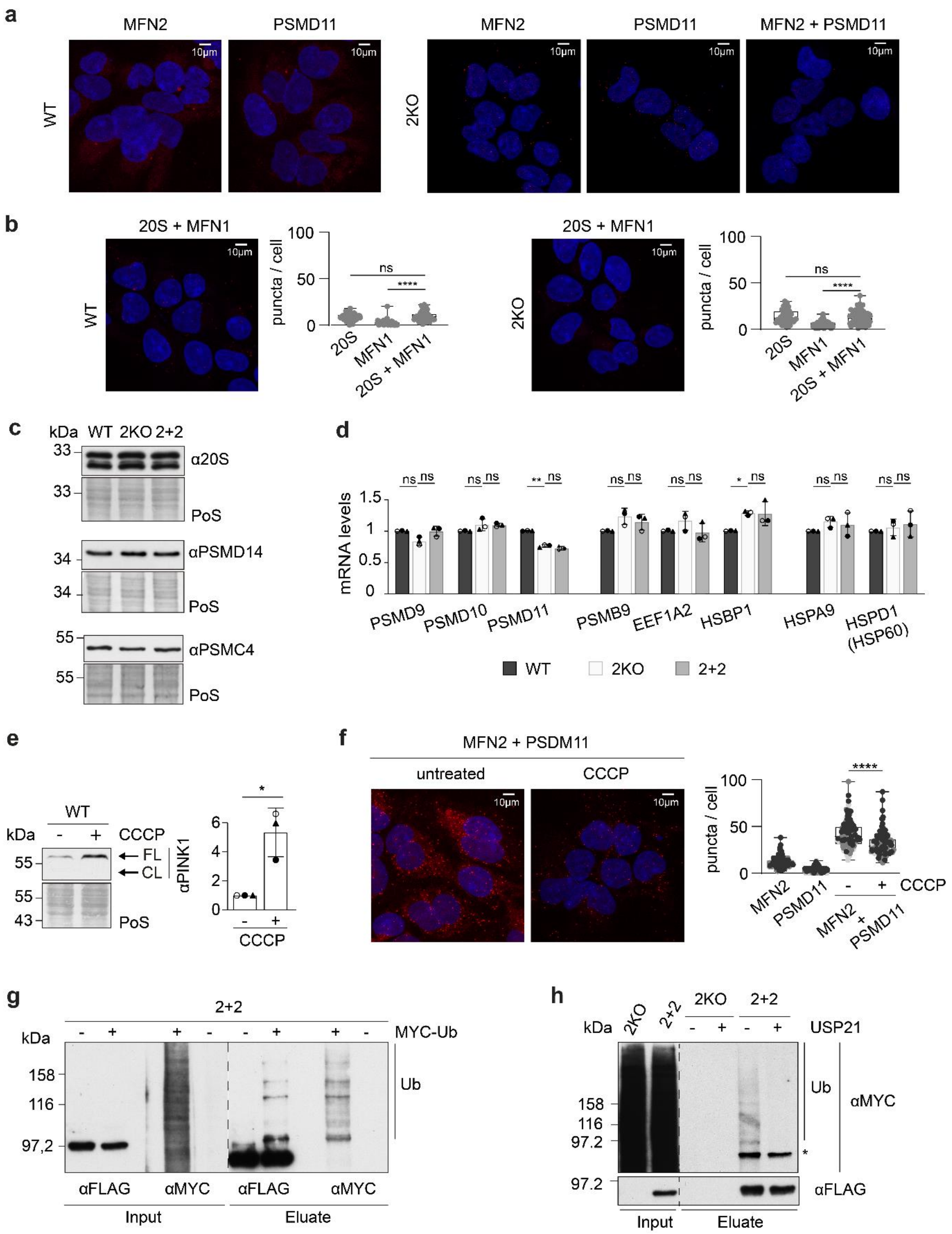

Supplementary Fig. 10: Analysis of MFN2 ubiquitylation of proteasome interaction. **(a)** Confocal images of proximity ligation assays of HEK WT and 2KO cells, with antibodies against MFN1 and 20S (in red), either used individually or together, and DAPI staining (in blue). Scale bar: 10µm.

(b) Confocal images of proximity ligation assays of HEK WT and 2KO cells, with antibodies against MFN2 and PSMD11 (in red), either used individually or together, and DAPI staining (in blue). Scale bar: 10µm. Quantification of number of puncta per cell using exclusively each of the antibodies or both (n=1 biological replicate). Ordinary one-way ANOVA was applied. **(c)** Western blot analysis of total cell lysates from HEK WT, 2KO and 2+2 cells immunoblotted with anti-20S (upper panel), anti-PSMD14 (middle panel) or anti-PSMC4 (lower panel). Staining of total protein with PoS was used as loading control. **(d)** Relative mRNA levels of PSMD9, PSMD10, PSMD11, PSMB9, EEF1A2, HSBP1, HSPA9 and HSPD1, measured by qPCR of total mRNA extracted from HEK WT, 2KO and 2+2 cells. Bars represent the average of transcript levels ± SD (n=3 biological replicates). Individual values of each experiment are discriminated in triangles and white or black filled circles. RM one-way ANOVA was applied. **(e)** Western blot analysis (left panel) and quantification (right panel) of total cell lysates from HEK WT cells untreated (-) or treated with CCCP (+; 20µM, 2h), immunoblotted with anti-PINK1 (FL: full-length; CL: cleaved). Staining of total protein with PoS was used as loading control. Bars represent the average fold change relative to WT ± SD (n=3 biological replicates). The individual values for each experiment are discriminated in triangles and white or black filled circles. **(f)** Confocal images of proximity ligation assay of HEK WT cells untreated (-) or treated with CCCP (20µM, 2h), with antibodies against MFN2 and PSMD11 (in red) and DAPI staining (in blue) (left panel). Scale bar: 10µm. Quantification of number of puncta per cell using exclusively each of the antibodies or both (right panel) (n=3 biological replicates). Ordinary one-way ANOVA was applied. **(g)** Western blot analysis of $MFN2^{FLAG}$, immunoprecipitated from total cell lysates of 2+2 cells, transiently transfected with an empty (-) or a MYC-ubiquitin (+) vector, immunoblotted with anti-FLAG and anti-MYC. **(h)** Western blot analysis of $MFN2^{FLAG}$, immunoprecipitated from HEK 2KO or 2+2 cells, untreated (-) or treated (+) with the DUB USP21, immunoblotted with anti-MYC and anti-FLAG. Source data and exact p values are provided as a Source Data file.

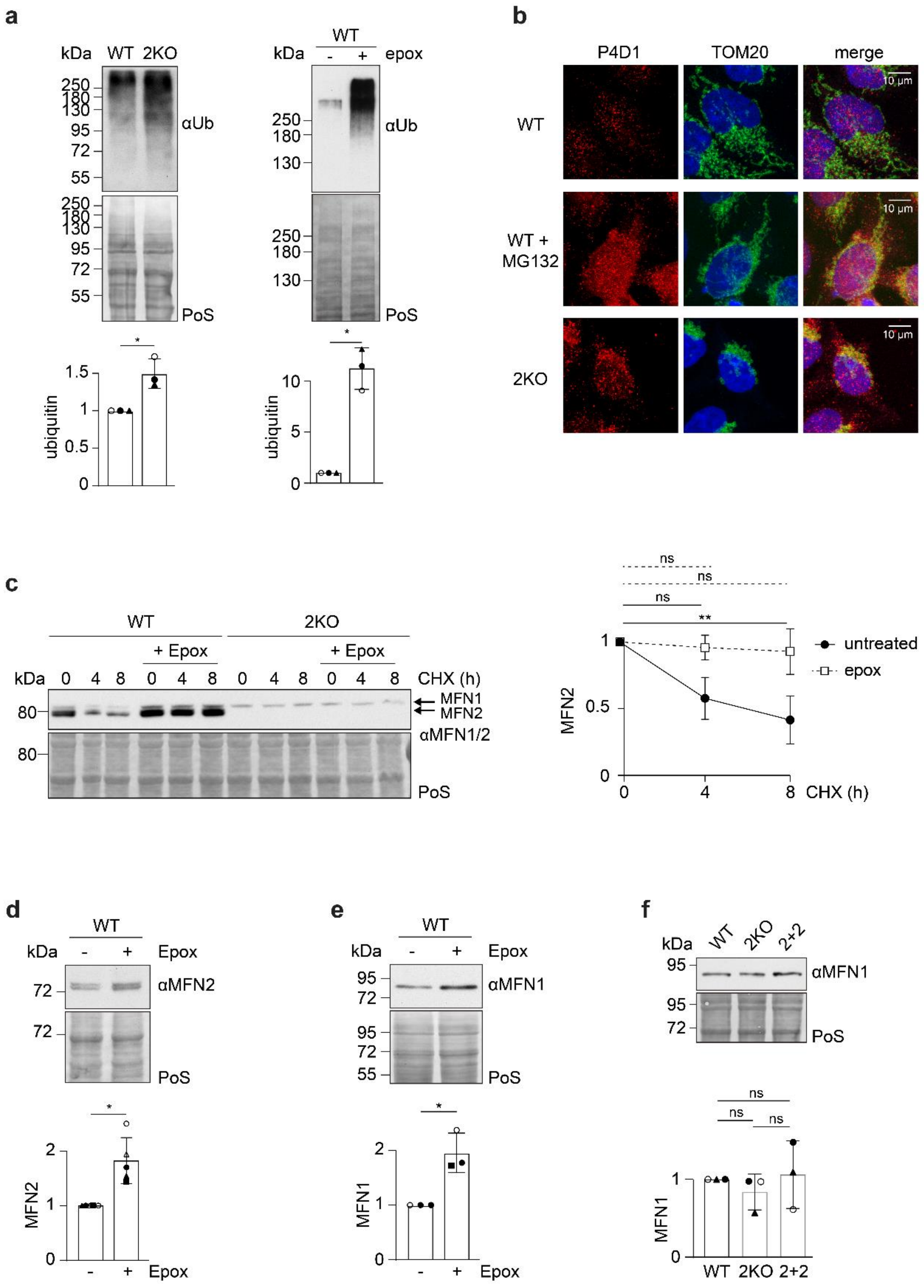

Supplementary Fig. 11: MFN1 and MFN2 are constutively regulated by the proteasome. **(a)** Western blot analysis (upper panels) and quantification (lower panels) of total cell lysates from HEK WT and 2KO cells (left panels) or HEK WT untreated (-) or treated (+) with epoxomicin (1µM, 2h) (right panels), immunoblotted with anti-ubiquitin. Staining of total protein with PoS was used

as loading control. Bars represent the average fold change relative to WT ± SD (n=4 biological replicates). Individual values of each experiment are discriminated in triangles and white or black filled circles. Paired t-test was applied. **(b)** Confocal images after immunostaining of P4D1 (in red), TOM20 (in green) and nucleus staining with DAPI (in blue) of HEK WT untreated or treated with MG132 (10μM, 9h) and 2KO cells. Scale bar: 10μm. **(c)** Western blot analysis (left panel) and quantification (of MFN2 levels, in WT cells, right panel) of total cell lysates from HEK WT and 2KO cells, untreated (0) or treated with cycloheximide (CHX, 100μg/mL) for 4 or 8h, and simultaneously treated with epoxomicin (1μM) when indicated, immunoblotted with anti-MFN1/2. Staining of total protein with PoS was used as loading control. Shown are the mean and SD of the individual timepoints, relative to the correspondent untreated control, labelled as "0" on the Western blot (n=3 biological replicates). Two-way ANOVA was applied. **(d-e)** Western blot analysis (upper panels) and quantification (lower panels) of total cell lysates from HEK WT cells untreated (-) or treated (+) with epoxomicin (1μM, 2h), immunoblotted with anti-MFN2 **(on d)** or anti-MFN1 **(on e)**. Staining of total protein with PoS was used as loading control. Bars represent the average fold change relative to WT ± SD (n=5 biological replicates for MFN2 and n=3 biological replicates for MFN1, respectively). Individual values of each experiment are discriminated in white or black filled squares, circles or triangles. Paired t-test was applied. **(f)** Western blot analysis (upper panel) and quantification (lower panel) of total cell lysates from HEK WT, 2KO and 2+2 cells, immunoblotted with anti-MFN1. Staining of total protein with PoS was used as loading control. Bars represent the average fold change relative to WT ± SD (n=3 biological replicates). Individual values of each experiment are discriminated in triangles and white or black filled circles. RM one-way ANOVA was applied. Source data and exact p values are provided as a Source Data file.

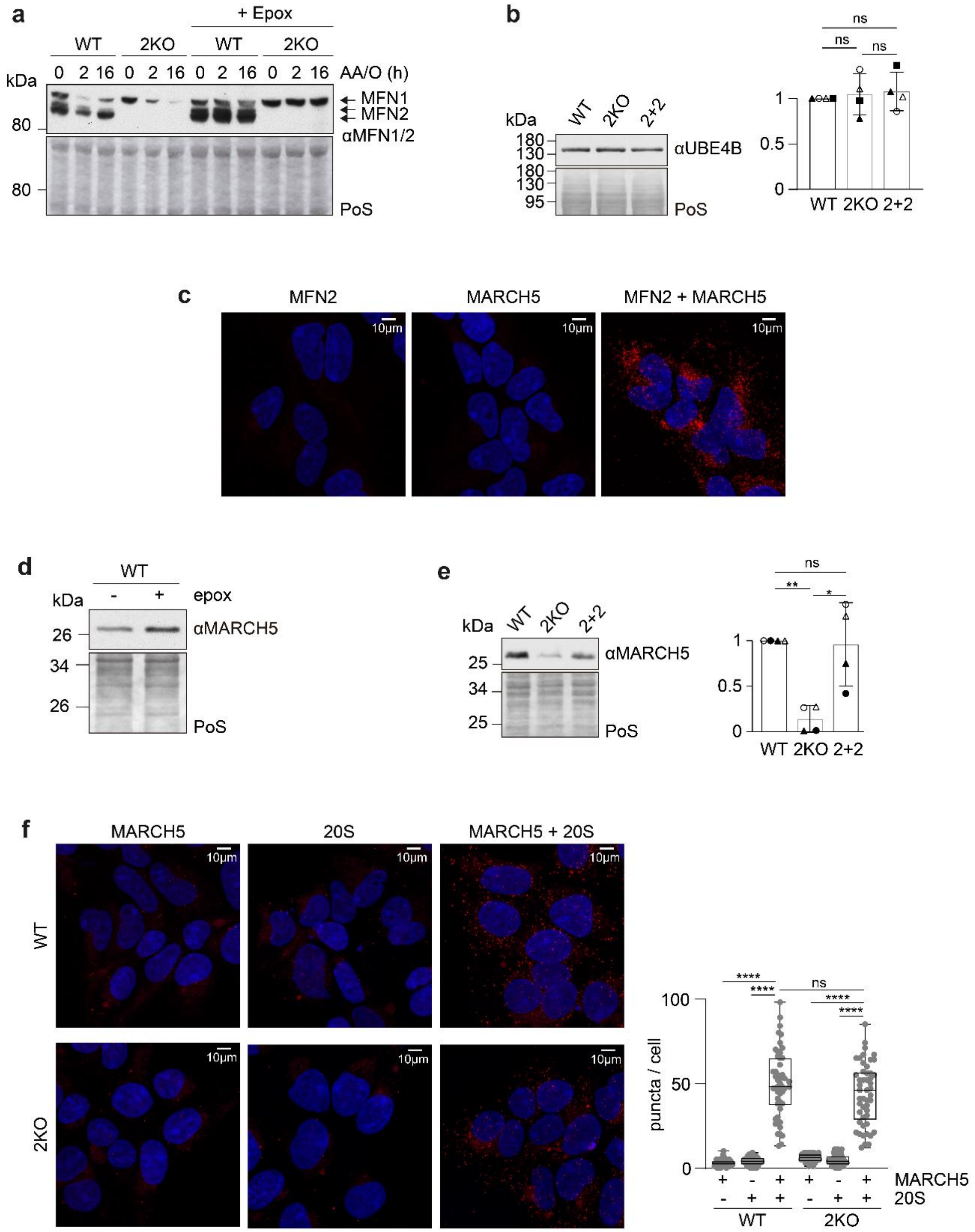

Supplementary Fig. 12: Effect of MFN2 on ubiquitin-related components. **(a)** Western blot analysis of total cell lysates from HEK WT and 2KO cells untreated (0) or treated with antimycin A (1µM) and oligomycin (10µM) (AA/O) for 2 or 16h, and simultaneously treated with epoxomicin (1µM), when indicated, and immunoblotted with anti-MFN1/2. Staining of total protein with PoS was used as loading control. **(b)** Western blot analysis (left panel) and quantification (right panel) of total cell lysates from HEK WT, 2KO and 2+2 cells, immunoblotted with anti-UBE4B. Staining

of total protein with PoS was used as loading control. Bars represent the average fold change relative to WT ± SD (n=4 biological replicates). Individual values of each experiment are discriminated in circles, squares and white or black filled triangles. RM one-way ANOVA was applied. **(c)** Confocal images of proximity ligation assay of HEK WT cells, with antibodies against MFN2 and MARCH5 (in red), either used individually or together, and DAPI staining (in blue). Scale bar: 10µm. **(d)** Western blot analysis of total cell lysates from HEK WT cells untreated (-) or treated with epoxomicin (+; 1µM, 2h), immunoblotted with anti-MARCH5. Staining of total protein with PoS was used as loading control. **(e)** Western blot analysis (left panel) and quantification (right panel) of total cell lysates from HEK WT, 2KO and 2+2 cells, immunoblotted with anti-MARCH5. Staining of total protein with PoS was used as loading control. Bars represent the average fold change relative to WT ± SD (n=4 biological replicates). Individual values of each experiment are discriminated in white or black filled circles and triangles. RM one-way ANOVA was applied. **(f)** Confocal images of proximity ligation assays of HEK WT and 2KO cells, with antibodies against 20S subunits and MARCH5 (in red), either used individually or together, and DAPI staining (in blue) (left panel). Scale bar: 10µm. Quantification of number of puncta per cell using exclusively each of the antibodies or both (right panel) (n=1 biological replicates). Ordinary one-way ANOVA was applied. Source data and exact p values are provided as a Source Data file.

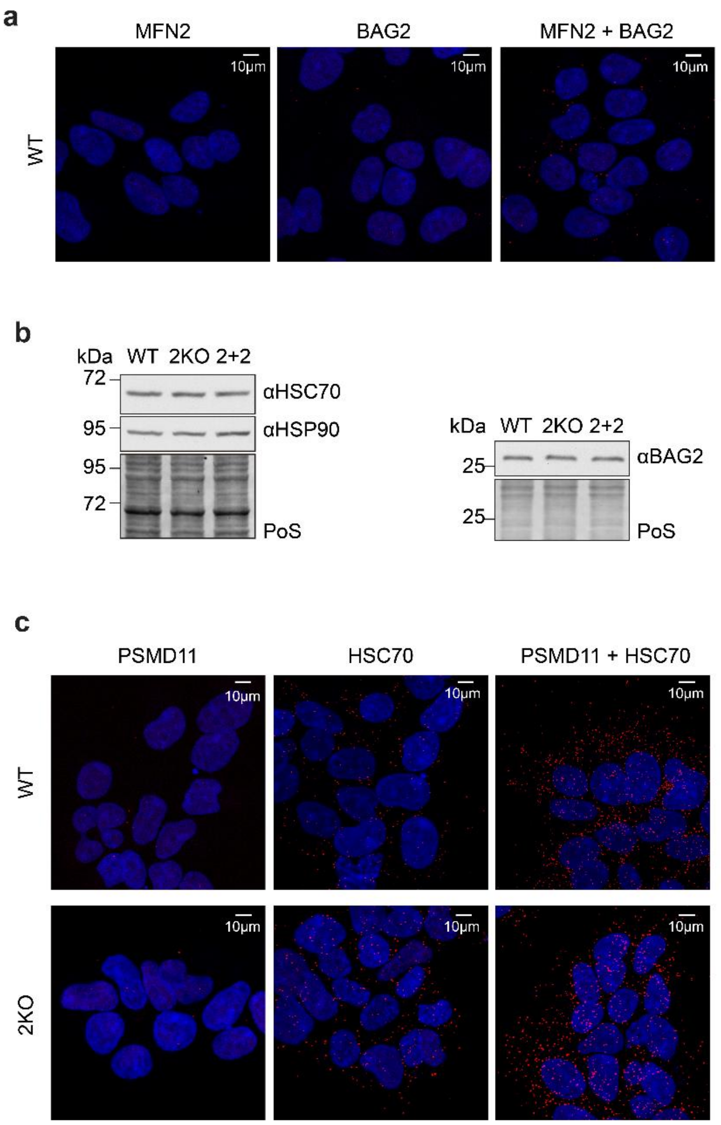

Supplementary Fig. 13: MFN2 effect of quality control components. **(a)** Confocal images of proximity ligation assay of HEK WT cells, with antibodies against MFN2 and BAG2 (in red), either used individually or together, and DAPI staining (in blue). Scale bar: 10µm. **(b)** Western blot analysis of total cell lysates from HEK WT, 2KO and 2+2 cells, immunoblotted with anti-HSC70, anti-HSP90 (left panel) or anti-BAG2 (right panel). Staining of total protein with PoS was used as loading control. **(c)** Confocal images of proximity ligation assays of HEK WT and 2KO cells, with antibodies against PSMD11 and HSC70 (in red), either used individually or together. Scale bar: 10µm. Source data are provided as a Source Data file.

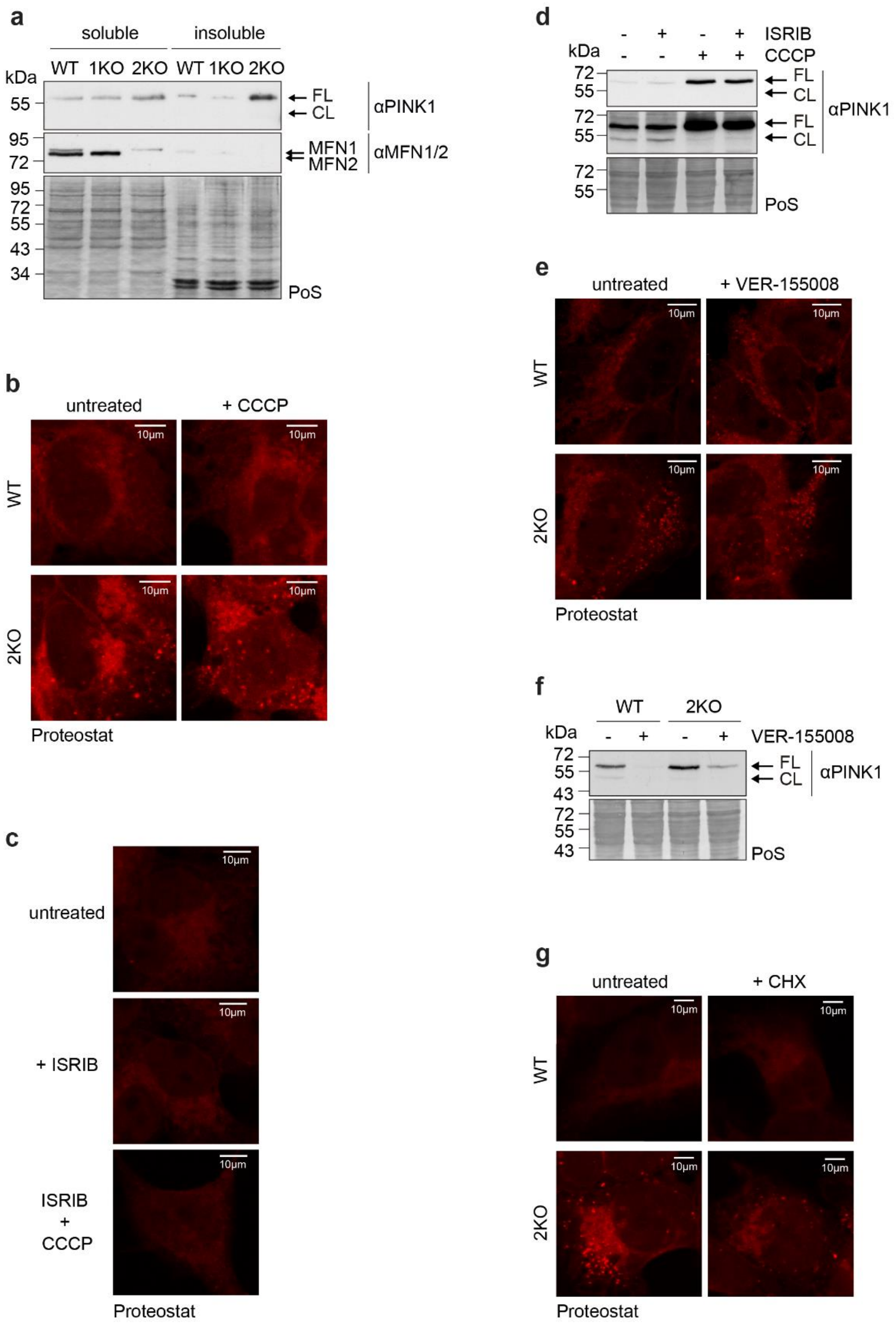

Supplementary Fig. 14: Effect of PINK1 on protein aggregation. **(a)** Western blot analysis of soluble and 8M urea-resistant insoluble fractions of HEK WT, 1KO and 2KO cells, immunoblotted with anti-PINK1 (FL: full-length; CL: cleaved) and anti-MFN1/2. Staining of total protein with PoS was used as loading control. **(b)** Confocal images of HEK WT and 2KO cells, untreated or treated

with CCCP (20µM, 2h), stained with PROTEOSTAT® Aggresome detection kit. Scale bar: 10µm. **(c)** Confocal images of HEK WT and 2KO cells, untreated or treated with ISRIB (5µM, 16h) alone or in combination with CCCP (20µM, 2h), stained with PROTEOSTAT® Aggresome detection kit. Scale bar: 10µm. **(d)** Western blot analysis of total cell lysates from HEK WT cells untreated (-,-), treated with ISRIB (+,-) (5µM, 16h), with CCCP (-,+) (20µM, 2h), or with both combined (+,+), immunoblotted with anti-PINK1 (FL: full-length; CL: cleaved). Staining of total protein with PoS was used as loading control **(e)** Confocal images of HEK WT and 2KO cells, untreated or treated with VER-155008 (+;50µM, 2h), stained with PROTEOSTAT® Aggresome detection kit. Scale bar: 10µm. **(f)** Western blot analysis of total cell lysates from HEK WT cells untreated (-) or treated with VER-155008 (+;50µM, 2h), immunoblotted with anti-PINK1 (FL: full-length; CL: cleaved). Staining of total protein with PoS was used as loading control. **(g)** Confocal images of HEK WT and 2KO cells, untreated or treated with CHX (5µM, 5h), stained with PROTEOSTAT® Aggresome detection kit. Scale bar: 10µm. Source data are provided as a Source Data file.

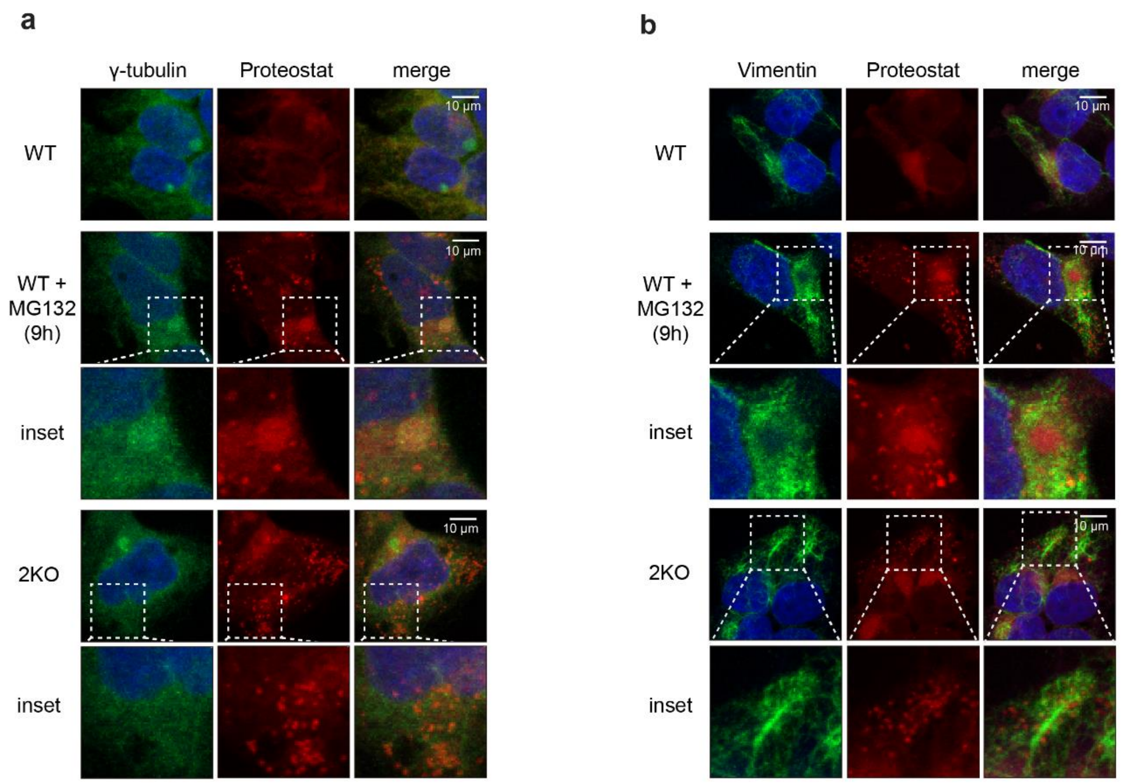

Supplementary Fig. 15: Co-localization of aggresomes markers with protein aggregates. **(a)** Confocal images of HEK WT untreated or treated with MG132 (5µM, 9h) and 2KO cells, co-stained with PROTEOSTAT® Aggresome detection kit (in red), Vimentin (in green) and DAPI (in blue). Scale bar: 10µm. Insets of white dotted boxes are shown below each image. **(b)** Confocal images of HEK WT untreated or treated with MG132 (5µM, 9h) and 2KO cells co-stained with PROTEOSTAT® Aggresome detection kit (in red), γ-tubulin (in green) and DAPI (in blue). Scale bar: 10µm. Insets of white dotted boxes are shown below each image.

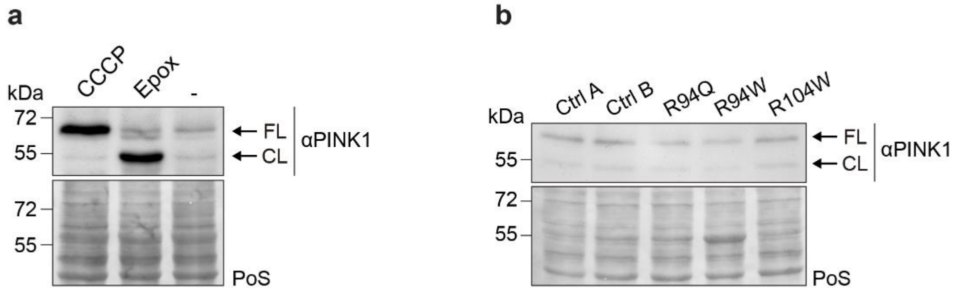

Supplementary Fig. 16: CMT2A patient fibroblasts do not exhibit increased PINK1. **(a)** Western blot analysis of total cell from healthy primary fibroblasts untreated (-) or treated with CCCP (20µM, 2h) or with Epoxomicin (1µM, 2h), immunoblotted with anti-PINK1 (FL: full-length; CL: cleaved). Staining of total protein with PoS was used as loading control. **(b)** Western blot analysis of total cell lysates from two control (A and B) and three CMT2A patients' primary fibroblasts, immunoblotted with anti-PINK1 (FL: full-length; CL: cleaved). Staining of total protein with PoS was used as loading control. Source data are provided as a Source Data file.

Supplementary Tables

Supplementary Table 1 – Plasmids

Plasmid	Resistance	Reference
pX335-hMFN1 CRISPR Cas9 construct 1	Ampicillin	This paper
pX335-hMFN1 CRISPR Cas9 construct 2	Ampicillin	This paper
pX335-hMFN2 CRISPR Cas9 construct 5	Ampicillin	This paper
pX335-hMFN2 CRISPR Cas9 construct 6	Ampicillin	This paper
MFN1-3xFLAG in pCDNA5	Amplicilin	This paper
MFN2-3xFLAG in pCDNA5	Amplicilin	This paper
MYC-UB in pCMV	Amplicilin	Provided by K. Hoffman
PINK1-HA in pCDNA3	Amplicilin	Provided by T. Langer
pLVX-puro-MtKeima	Puromycin	Provided by T. Finkel
pMD2.G	Amplicilin	Addgene #12259
pMDLg/pRRE	Amplicilin	Addgene #12251
pOG44 in pCMV	Hygromycin	ThermoFisher Scientific #V600520
pRSV-Rev	Amplicilin	Addgene #12253
AKAP1-mRFP	Amplicilin	Provided by G. Hajnoczky
AKAP1-mRFP-UBC6	Amplicilin	Provided by G. Hajnoczky

Supplementary Table 2 – siRNAs

siRNA	Reference
siMFN1	ThermoFisher Scientific ID: HSS124777
siMFN2	ThermoFisher Scientific ID: HSS115027

Supplementary Table 3 – Primary Antibodies

Antibody	Reference	Dilution	Method
ATP5α	Abcam #ab14748	1:1000	WB
ATP5β	Thermo Fisher #A21351	1:500	IF
BAG2	Abcam #ab79406	1:300; 1:500	WB; PLA
DRP1	BD Biosciences #611113	1:1000	WB
FLAG	Sigma #F1804	1:1000	WB
FLAG	Sigma #F1804	1:500	IF
GM130	BD Biosciences #610822	1:1000	WB
HSC70	Abnova #MAB6636	1:1000; 1:500	WB; PLA
HSP90	Thermo Fisher #MA1-10372	1:1000	WB
LAMP1	Abcam #24170	1:500	IF
LC3	Cell Signalling #2775	1:1000	WB
MARCH5	Cell Signaling #19168S	1:1000; 1:500	WB; PLA
Mitofusin 1/2	Abnova #H00055669-M04	1:1000	WB

Mitofusin 1	Cell Signaling #14739	1:500	WB
Mitofusin 2	Abcam #ab50838	1:1000; 1:500	WB; PLA
Mitofusin 2 (murine)	Abcam #ab56889	1:1000	WB
MT-CO1	Molecular Probes #459600	1:1000; 1:500	WB; PLA
MYC	Cell Signaling #2276	1:1000	WB
NDUF9A	Molecular Probes #459100	1:1000	WB
OPA1	BD Biosciences #612606	1:1000	WB
p62	Abcam #ab155686	1:1000	WB
PARL	Genscript, costumized	1:1000	WB
PARKIN	Sigma #P6248	1:1000	WB
PINK1	Cell Signalling #6946T	1:500	WB
PSMC4	Bethyl laboratories #A303-850A	1:1000	WB
PSMD11	Novus Biologicals # NBP1-46191	1:500	PLA
PSMD14	Cell Signalling #4197	1:1000	WB
STIP1	Abcam, #ab126753	1:300	WB
TOM20	Sigma #HPA011562	1:1000; 1:500	WB; IF

TOM20 (murine)	Santa Cruz, #sc-11415	1:1000	WB
UBE4B	Proteintech, #18148-1-AP	1:1000	WB
ubiquitin	Cell Signaling (P4D1) #3936	1:1000; 1:500	WB; IF
ubiquitin	Cell Signalling, #43124	1:1000	WB
UQCRC1	Molecular Probes #459140	1:1000	WB
vimentin	Thermo Fisher, #MA5-16409	1:400	IF
20S proteasome α1,2,3,4,5,6,7	Enzo #BML-PW8195-0100	1:1000; 1:500	WB; PLA
α-tubulin	Sigma #T6074	1:500	IF
γ-tubulin	Sigma, #T6557	1:400	IF

Supplementary Table 4 – qPCR primers

**Primer name**	**Sequence**
TOM20_F	GAGCTGGGCTTTCCAAGTTAC
TOM20_R	TTGTCAGATGGTCTACGCCC
DRP1_F	ACCTGCTTCCCAGAGGTACT
DRP1_R	TCTGCTTCCACCCCATTTTCT
MARCH5_F	GATGCTGGACAGAAGTTGCTGG
MARCH5_R	CCACTCTGGCTGTACTGTTTCC
OPA1_F	TGTGAGGTCTGCCAGTCTTTA
OPA1_R	TGTCCTTAATTGGGGTCGTTG

HSPA9B_F	AATTACTTGGGGCACACAGC
HSPA9B_R	CGAAGCACATTCAGTCCAGA
HSPD1_F	CGTCTTGAATAGGCTAAAGG
HSPD1_R	TGAACGTCTTCAAGATTCAG
HSPB1_F	AAGCTAGCCACGCAGTCCAA
HSPB1_R	CGACTCGAAGGTGACTGGGA
EEF1A2_F	TGCACCACGAGGCTCTGA
EEF1A2_R	TGCTGTCCCCACACACGTT
NDUFA9_F	TGACCCAGGGTTGGTCATTG
NDUFA9_R	GGGCAGGTGGAGAACTTACC
UQCRC1_F	CAGTCCTCTCAGCCCACTTG
UQCRC1_R	AAGCCAGATGCTCCAAAAAG
MT-CO1_F	GACGTAGACACACGAGCATATTTCA
MT-CO1_R	AGGACATAGTGGAAGTGAGCTACAAC
ATP5a_F	GCAGGCCTAAATACACTGCC
ATP5a_F	TCTCAAATGTTCACCACCCCC
SDHA_F	TGCTGCCGTGTTCCGTGTGG
SDHA_R	ACCATTCCCCGGTCGAACGTCT
PSMD9_F	AAGGCCAACTATGACGTGCTG
PSMD9_R	ATATGATGTTGTGCCTGGCG
PSMD10_F	GCAGCTTCGAAAAACAGGCA
PSMD10_F	GGATGTTTGTGGATGCTTTG
PSMD11_F	GCCATCTACTGCCCCCCTAA
PSMD11_R	ATGGATAATACCCGACTGCATGT

Supplementary Data

Supplementary Data 1: LC-MS/MS analysis of the whole cell proteome of HEK WT, 1KO, 2KO and 2+2 cells (n=4 biological replicates). The pairwise comparison using two-sided t-test and permutation based FDR correction (FDR < 0.05, number permutation = 500, s0=0.1), the data normalized to the mitochondrial mass, and the 1D pathway enrichment are included.

Supplementary Data 2: LC-MS/MS analysis of the interactome of immunoprecipitated MFN1-3x-FLAG or MFN2-3x-FLAG in 1KO or 2KO cells, respectively (n=3 biological replicates). Pairwise comparisons were made using an unpaired two-sided t-test. The p-value was adjusted by the Benjamini Hochberg correction procedure (FDR <= 0.05).
