# Supplementary material for: Mitofusin 2 displays fusion-independent roles in proteostasis surveillance
Source: Nat Commun. 2025 Feb 10;16:1501. doi: 10.1038/s41467-025-56673-5 (PMC11811173; doi:10.1038/s41467-025-56673-5)
Supplement: Supplementary file 2 — Description of Additional Supplementary Files [file 41467_2025_56673_MOESM2_ESM.pdf]

Description of Additional Supplementary Files

Supplementary Data 1 - MS analysis of whole proteome of HEK WT 1KO 2KO and 2+2 cells.

Supplementary Data 2 - MS analysis of interactome of MFN1-FLAG and MFN2-FLAG.
