# Supplementary material for: Mitofusin 2 displays fusion-independent roles in proteostasis surveillance
Source: Nat Commun. 2025 Feb 10;16:1501. doi: 10.1038/s41467-025-56673-5 (PMC11811173; doi:10.1038/s41467-025-56673-5)
Supplement: Supplementary file 5 — Reporting Summary [file 41467_2025_56673_MOESM5_ESM.pdf]

nature portfolio

Corresponding author(s): Mafalda Escobar-Henriques

Last updated by author(s): Dec 18, 2024

Reporting Summary

Nature Portfolio wishes to improve the reproducibility of the work that we publish. This form provides structure for consistency and transparency in reporting. For further information on Nature Portfolio policies, see our Editorial Policies and the Editorial Policy Checklist.

Statistics

For all statistical analyses, confirm that the following items are present in the figure legend, table legend, main text, or Methods section.

n/a	Confirmed	
☐	☒	The exact sample size (*n*) for each experimental group/condition, given as a discrete number and unit of measurement
☒	☐	A statement on whether measurements were taken from distinct samples or whether the same sample was measured repeatedly
☐	☒	The statistical test(s) used AND whether they are one- or two-sided<br>*Only common tests should be described solely by name; describe more complex techniques in the Methods section.*
☒	☐	A description of all covariates tested
☒	☐	A description of any assumptions or corrections, such as tests of normality and adjustment for multiple comparisons
☐	☒	A full description of the statistical parameters including central tendency (e.g. means) or other basic estimates (e.g. regression coefficient) AND variation (e.g. standard deviation) or associated estimates of uncertainty (e.g. confidence intervals)
☐	☒	For null hypothesis testing, the test statistic (e.g. *F*, *t*, *r*) with confidence intervals, effect sizes, degrees of freedom and *P* value noted<br>*Give P values as exact values whenever suitable.*
☒	☐	For Bayesian analysis, information on the choice of priors and Markov chain Monte Carlo settings
☒	☐	For hierarchical and complex designs, identification of the appropriate level for tests and full reporting of outcomes
☒	☐	Estimates of effect sizes (e.g. Cohen's *d*, Pearson's *r*), indicating how they were calculated

Our web collection on statistics for biologists contains articles on many of the points above.

Software and code

Policy information about availability of computer code

Data collection	Microscopic images were acquired with UltraView Vox (Perkin Elmer), Stellaris 5 (Leica) and TCS SP8 gSTED (Leica) microscopes.
Data analysis	Mass spectometry data was analyzed with Instant Clue v.0.10.10.20210316<br>Confocal microscopy images were analyzed with ImageJ v.1.53c<br>Statistical analysis was performed with GraphPad Prims v.9.4.1

For manuscripts utilizing custom algorithms or software that are central to the research but not yet described in published literature, software must be made available to editors and reviewers. We strongly encourage code deposition in a community repository (e.g. GitHub). See the Nature Portfolio guidelines for submitting code & software for further information.

Data

Policy information about availability of data

All manuscripts must include a data availability statement. This statement should provide the following information, where applicable:

- Accession codes, unique identifiers, or web links for publicly available datasets
- A description of any restrictions on data availability
- For clinical datasets or third party data, please ensure that the statement adheres to our policy

All relevant data are included in the paper and/or the Supplementary Materials. Source data are provided with this paper. The whole proteome data generated in this study have been deposited in the PRIDE database under accession code PXD058425 [https://www.ebi.ac.uk/pride/archive/projects/PXD058425].

Research involving human participants, their data, or biological material

Policy information about studies with human participants or human data. See also policy information about sex, gender (identity/presentation), and sexual orientation and race, ethnicity and racism.

Reporting on sex and gender	Human primary fibroblasts sex and age information:<br>Healthy individual A: male, 55 years old;<br>Healthy individual B: male, 46 years old;<br>CMT2A patient carrying the MFN2 mutation R94Q: female, 60 years old;<br>CMT2A patient carrying the MFN2 mutation R94W: female, 43 years old;<br>CMT2A patient carrying the MFN2 mutation R104W: male, 30 years old.<br>Sex determination was indicated in the file by the patient's declaration. Gender was not considered in this study since to date, no clinical data have established a link between the CMT2A phenotype and gender. The gender of the samples was not taken into account. However, we use a balanced mixture of male and female fibroblasts for quantitative western-blot measurements.
Reporting on race, ethnicity, or other socially relevant groupings	No socially relevant groupings were performed.
Population characteristics	No population characteristics were considered.
Recruitment	Patients were sampled during a neurological consultation by a clinical neurologist in CHU Angers. A confirmed pathogenic mutation was required.
Ethics oversight	For the human primary fibroblasts used in this study, written informed consent was obtained from all participants (Ethics Committee from the Angers University Hospital approval: CPP Ouest 6 – Angers, France; Identification number: CPP1402 HPS2.; Declaration number: 21.04.27.3982; Authorization number: 2021-A00837-34. No patient was remunerated during this study. The witnesses were granted a travel allowance of 30 euros.

Note that full information on the approval of the study protocol must also be provided in the manuscript.

Field-specific reporting

Please select the one below that is the best fit for your research. If you are not sure, read the appropriate sections before making your selection.

☒ Life sciences ☐ Behavioural & social sciences ☐ Ecological, evolutionary & environmental sciences

For a reference copy of the document with all sections, see nature.com/documents/nr-reporting-summary-flat.pdf

Life sciences study design

All studies must disclose on these points even when the disclosure is negative.

Sample size	The figure legends / Statistics and Reproducibility section states how many biological replicates were performed for each experiment. For the quantification of each experiment, at least 3 distinct biological replicates were used. For quantification of cellular characteristics, at least 100 cells per biological replicate were considered. These sample sizes are the commonly used ones for the techniques used in this manuscript.
Data exclusions	No data was excluded
Replication	Quantifications of biological replicates have been analyzed statistically. All replications were sucessfull.
Randomization	No randomization has been done
Blinding	Since group allocation did not occur, blinding was not relevant in our study

Reporting for specific materials, systems and methods

We require information from authors about some types of materials, experimental systems and methods used in many studies. Here, indicate whether each material, system or method listed is relevant to your study. If you are not sure if a list item applies to your research, read the appropriate section before selecting a response.

Materials & experimental systems

n/a	Involved in the study	
☐	☒	Antibodies
☐	☒	Eukaryotic cell lines
☒	☐	Palaeontology and archaeology
☐	☒	Animals and other organisms
☒	☐	Clinical data
☒	☐	Dual use research of concern
☒	☐	Plants

Methods

n/a	Involved in the study	
☒	☐	ChIP-seq
☒	☐	Flow cytometry
☒	☐	MRI-based neuroimaging

Antibodies

Antibodies used

ATP5α Abcam #ab14748
ATP5β Thermo Fisher #A21351
BAG2 Abcam #ab79406
DRP1 BD Biosciences #611113
FLAG Sigma #F1804
FLAG Sigma #F1804
GM130 BD Biosciences #610822
HSC70 Abnova #MAB6636
HSP90 Thermo Fisher #MA1-10372
LAMP1 Abcam #24170
LC3 Cell Signalling #2775
MARCH5 Cell Signaling #19168S
Mitofusin 1/2 Abnova #H00055669-M04
Mitofusin 1 Cell Signaling #14739
Mitofusin 2 Abcam #ab50838
Mitofusin 2 Abcam #ab56889
MT-CO1 Molecular Probes #459600
MYC Cell Signaling #2276
NDUF9A Molecular Probes #459100
OPA1 BD Biosciences #612606
p62 Abcam #ab155686
PARL Genscript, costumized
PARKIN Sigma #P6248
PINK1 Cell Signalling #6946T
PSMC4 Bethyl laboratories #A303-850A
PSMD11 Novus Biologicals # NBP1-46191
PSMD14 Cell Signalling #4197
SDHA Molecular Probes #459200
STIP1 Abcam, #ab126753
TOM20 Sigma #HPA011562
TOM20 Santa Cruz, #sc-11415
UBE4B Proteintech, #18148-1-AP
ubiquitin Cell Signaling (clone P4D1) #3936
ubiquitin Cell Signalling, #43124
UQCRC1 Molecular Probes #459140
vimentin Thermo Fisher, #MA5-16409
20S proteasome α1,2,3,4,5,6,7 Enzo #BML-PW8195-0100
α-tubulin Sigma #T6074
γ-tubulin Sigma, #T6557

Validation

ATP5α, Abcam #ab14748:
https://www.abcam.com/en-us/products/primary-antibodies/atp5a-antibody-15h4c4-mitochondrial-marker-ab14748?srsltid=AfmBOoo1753JBxjupCDAFQptNav3sBX_SuDQbo5UN-l1I1ge-VDe6F3T
ATP5β, Thermo Fisher #A21351:
https://www.thermofisher.com/antibody/product/ATP-Synthase-beta-Antibody-clone-3D5AB1-Monoclonal/A-21351
BAG2, Abcam #ab79406:
https://www.abcam.com/en-us/products/primary-antibodies/bag2-antibody-epr3567-ab79406?srsltid=AfmBOorIKJ5tBd5piQNI-wSoJYdPNGfhvaaTYE4Sq3TtT_vVpLHxRS6N
DRP1, BD Biosciences #611113:
https://www.bdbiosciences.com/en-de/products/reagents/microscopy-imaging-reagents/immunofluorescence-reagents/purified-mouse-anti-dlp1.611113?tab=product_details
FLAG, Sigma #F1804:
https://www.sigmaaldrich.com/DE/en/product/sigma/f1804
GM130, BD Biosciences #610822:
https://www.bdbiosciences.com/en-de/products/reagents/microscopy-imaging-reagents/immunofluorescence-reagents/purified-mouse-anti-gm130.610822?tab=product_details
HSC70, Abnova #MAB6636:
https://www.abnova.com/en-global/product/detail/mab6636

HSP90, Thermo Fisher #MA1-10372:
https://www.thermofisher.com/antibody/product/HSP90-Antibody-clone-MBH90AB-Monoclonal/MA1-10372
LAMP1, Abcam #24170:
https://www.abcam.com/en-us/products/primary-antibodies/lamp1-antibody-lysosome-marker-ab24170?srsltid=AfmBOorIMalIVeshLRKELkPFJzY6SOiyR4nY-4LdXzvrFnhwzy5E0vPr
LC3, Cell Signalling #2775:
https://www.cellsignal.com/products/primary-antibodies/lc3b-antibody/2775?srsltid=AfmBOoohjWNlwrm6yWp2Lw77Zh5bnoC5UsmSxUWTUdYcQTc88IFnFD6j
MARCH5, Cell Signaling #19168S:
https://www.cellsignal.com/products/primary-antibodies/march5-antibody/19168?srsltid=AfmBOoowppWFZc07K7sePXxPTVmOUIO-5PkfhtTsuJiQp60jVzqwE28z
Mitofusin 1/2, Abnova #H00055669-M04:
https://www.abnova.com/en-global/product/detail/H00055669-M04
Mitofusin 1, Cell Signaling #14739:
https://www.cellsignal.com/products/primary-antibodies/mitofusin-1-d6e2s-rabbit-mab/14739?srsltid=AfmBOoosLuri_KnWoq8Tp1fnaeDkx2sitf7P_KLP7LzwOILsSGmP0nTk
Mitofusin 2, Abcam #ab50838:
https://www.abcam.com/en-us/products/primary-antibodies/mitofusin-2-antibody-ab50838?srsltid=AfmBOoqhNywQra1OVnPjXyOuMrIWvhuS68izzA44KI3VvllBXlmimBqf
Mitofusin 2, Abcam #ab56889:
https://www.abcam.com/en-us/products/primary-antibodies/mitofusin-2-antibody-6a8-ab56889?srsltid=AfmBOoroJRDi8OjfEX8ccbH-FmFaD5TWnnfA2JIYVlVqOuKv3GXeywQ9
MT-CO1, Molecular Probes #459600:
https://www.fishersci.com/shop/products/mtco1-monoclonal-antibody-1d6e1a8-invitrogen/459600
MYC, Cell Signaling #2276:
https://www.cellsignal.com/products/primary-antibodies/myc-tag-9b11-mouse-mab/2276?srsltid=AfmBOoqAxS66mtXDNkHf7oRnHouu0hK9lL5a7iFEVJoex87AGqrRhJTW
NDUF9A, Molecular Probes #459100:
https://www.thermofisher.com/antibody/product/NDUFA9-Antibody-clone-20C11B11B11-Monoclonal/459100
OPA1, BD Biosciences #612606:
https://www.bdbiosciences.com/en-de/products/reagents/microscopy-imaging-reagents/immunofluorescence-reagents/purified-mouse-anti-opa1.612606
p62, Abcam #ab155686:
https://www.abcam.com/en-us/products/primary-antibodies/sqstm1-p62-antibody-ab155686?srsltid=AfmBOoq_DoU4W0tku4uVhe3o1Jv-MLLwqCd7P8hft4HuZ_PXGY3yPPTk
PARL, Genscript, costumized – Shotaro et al, 2017, Nature Cell Biology, doi: 10.1038/ncb3488
PARKIN, Sigma #P6248:
https://www.sigmaaldrich.com/DE/en/product/sigma/p6248?srsltid=AfmBOoq51Q2zc8p91aP9zu4nFwcYCQSk1nXjqRlhy-WNMIdw9B01CzM7
PINK1, Cell Signalling #6946T:
https://www.cellsignal.com/products/primary-antibodies/pink1-d8g3-rabbit-mab/6946?srsltid=AfmBOopm55Y4tPHgsshT-OY7OrvQ6aU8xJhU1-jLAy_PE17EAQTaCaSh
PSMC4, Bethyl laboratories #A303-850A:
https://www.thermofisher.com/antibody/product/PSMC4-Antibody-Polyclonal/A303-850A
PSMD11, Novus Biologicals # NBP1-46191:
https://www.novusbio.com/products/psmd11-antibody_nbp1-46191?srsltid=AfmBOoqFa6wCqLsLyWYfvU0enn0CaVAdRRaz35-SvNBgI-1Q84QflWIa
PSMD14, Cell Signalling #4197:
https://www.cellsignal.com/products/primary-antibodies/psmd14-d18c7-rabbit-mab/4197?srsltid=AfmBOooXJJS27vWT5_CvuGWXejxhOyx1pfNr6bHOtBlOw3yZyYMbNOgc
SDHA, Molecular Probes #459200:
https://www.fishersci.com/shop/products/sdha-monoclonal-antibody-2e3gc12fb2ae2-invitrogen/459200
STIP1, Abcam #ab126753:
https://www.abcam.com/en-us/products/primary-antibodies/stip1-sti1-antibody-epr6606-ab126753?srsltid=AfmBOopJ4wvZ9mKNR_ov4EPFQAPkY6iEmBqnRhAOMqiqdf5bXnuceuDN
TOM20, Sigma #HPA011562:
https://www.sigmaaldrich.com/DE/en/product/sigma/hpa011562?srsltid=AfmBOorm77U9ZltoLME15lRbkbtTWGT7ImyO7h8dL9umRrye1s-Vl32M
TOM20, Santa Cruz #sc-11415:
https://www.scbt.com/p/tom20-antibody-fl-145?srsltid=AfmBOooG5maFR2BDj64ZvtywbT35NuKYC4ZlNHPJ-YhIbiMLVLMhXB3z
UBE4B, Proteintech #18148-1-AP:
https://www.ptglab.com/products/UBE4B-Antibody-18148-1-AP.htm?srsltid=AfmBOooL9b5T-GyOXG073u2b8SA2c5P_esgtf6iC80Ol6ExYyCQqWFVm
ubiquitin, Cell Signaling (clone P4D1) #3936:
https://www.cellsignal.com/products/primary-antibodies/ubiquitin-p4d1-mouse-mab/3936?srsltid=AfmBOoq4dK4Is7_W3ntRl7YAw_GsJXHoMI4mY7irfJ3RPG0Om26yVVQU
ubiquitin, Cell Signalling #43124:
https://www.cellsignal.com/products/primary-antibodies/ubiquitin-e4i2j-rabbit-mab/43124?srsltid=AfmBOooFJAs4swCCnxcbODHQE8pQj8gxHaUTQrO0tKBHmOPL0-ByAjb6
UQCRC1, Molecular Probes #459140: https://www.thermofisher.com/antibody/product/UQCRC1-Antibody-clone-16D10AD9AH5-Monoclonal/459140
Vimentin, abcam #ab16700:
https://www.abcam.com/en-us/products/primary-antibodies/vimentin-antibody-sp20-ab16700?srsltid=AfmBOop9G6775_K7k-6jE1wIiT8hCKSiXC58bsirJ-cx2bMQ5z7uV4Ly
20S proteasome α1,2,3,4,5,6,7, Enzo #BML-PW8195-0100: https://www.enzo.com/product/proteasome-20s-%CE%B11-2-3-5-6-7-subunits-monoclonal-antibody-mcp231/

α-tubulin, Sigma #T6074:
https://www.sigmaaldrich.com/DE/en/product/sigma/t6074?srsltid=AfmBOoo-QZwQq1inHtoFseQQcCRvS-ipFnu9tjdhlN0dW_DylJyGY69n
γ-tubulin, Sigma #T6557: https://www.sigmaaldrich.com/DE/en/product/sigma/t6557?srsltid=AfmBOoqNG3F3f5Tw9IDML8bQvbbLP0-QSNS7_N0J8Rad5Hnb_lK0SE_P

Eukaryotic cell lines

Policy information about cell lines and Sex and Gender in Research

Cell line source(s)	HEK 293: purchased from Invitrogen, R75007<br>HeLa: purchased from ATCC, CCL-2
Authentication	None of the cell lines were autenthicated
Mycoplasma contamination	All cell lines tested negative for mycoplasma
Commonly misidentified lines (See ICLAC register)	No commonly misidentified cell lines were used in this study

Animals and other research organisms

Policy information about studies involving animals; ARRIVE guidelines recommended for reporting animal research, and Sex and Gender in Research

Laboratory animals	Mus Musculus, C57/BL6N, embryonic day 14, 5-7 embryos of both sexes per litter from at least 3 pregnant females of both sexes. For more details, please refer to Methods sections “Mouse model” and “Neuronal Cultures”
Wild animals	The study did not involve wild animals
Reporting on sex	Both male and female embryos were pooled to generate primary neuronal cultures.
Field-collected samples	This study did not involve samples collected from the field
Ethics oversight	All animal procedures were conducted in accordance with the European, national, and institutional guidelines and were approved by the Landesamt für Natur, Umwelt und Verbraucherschutz, Nordrhein-Westfalen, Germany. Also followed the guidelines of the Federation of European Laboratory Animal Science Associations.

Note that full information on the approval of the study protocol must also be provided in the manuscript.

Plants

Seed stocks	This study did not involve seed stocks
Novel plant genotypes	This study did not involve novel plant genotypes
Authentication	This study did not involve autenthication of any of the above
