# Supplementary material for: Mitofusin 2 displays fusion-independent roles in proteostasis surveillance
Source: Nat Commun. 2025 Feb 10;16:1501. doi: 10.1038/s41467-025-56673-5 (PMC11811173; doi:10.1038/s41467-025-56673-5)

Source Data Main Figures

Fig. 2a

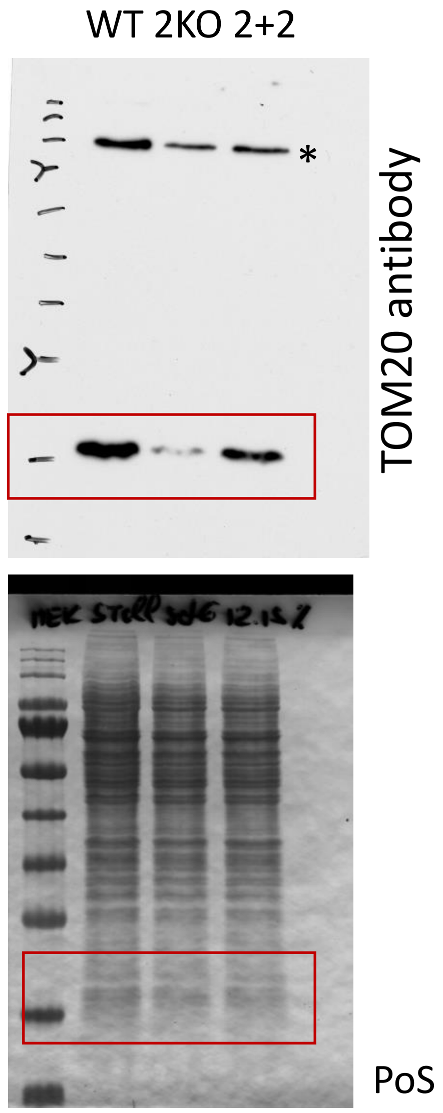

* cross-reaction

Fig. 2b

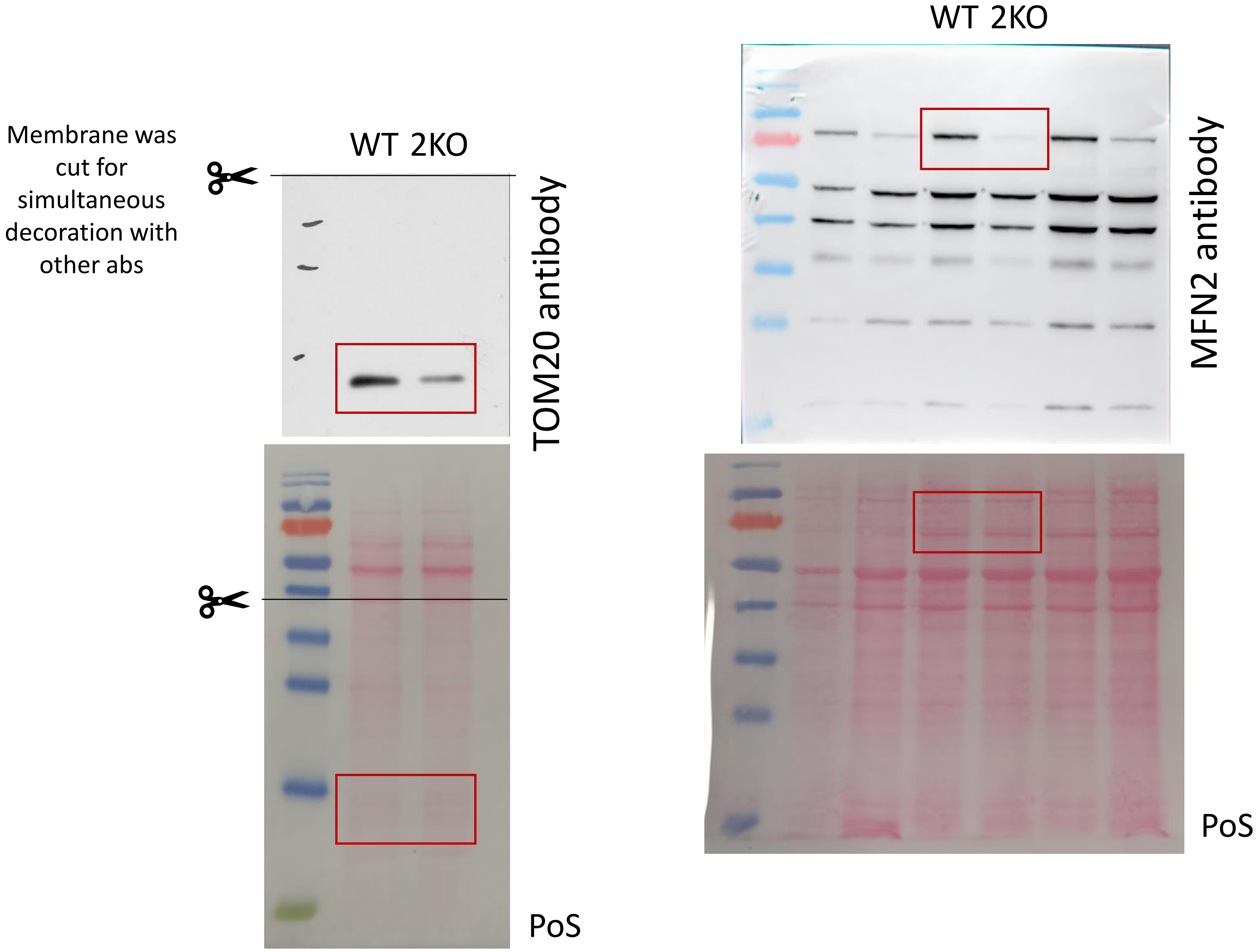

Fig. 2c

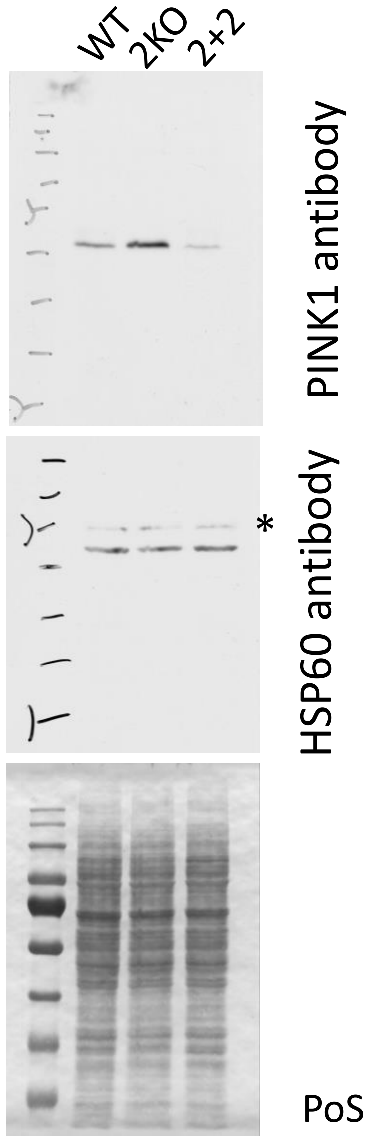

Fig. 2d

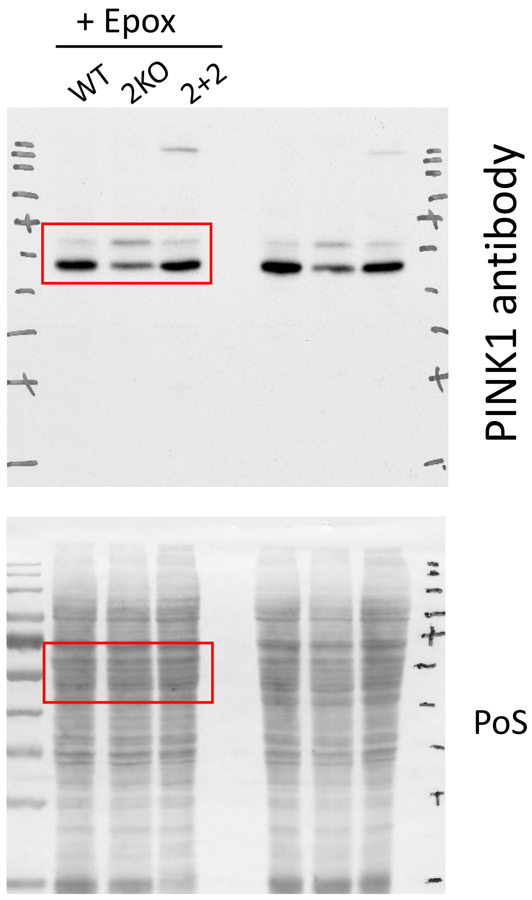

* cross-reaction

Fig. 2e

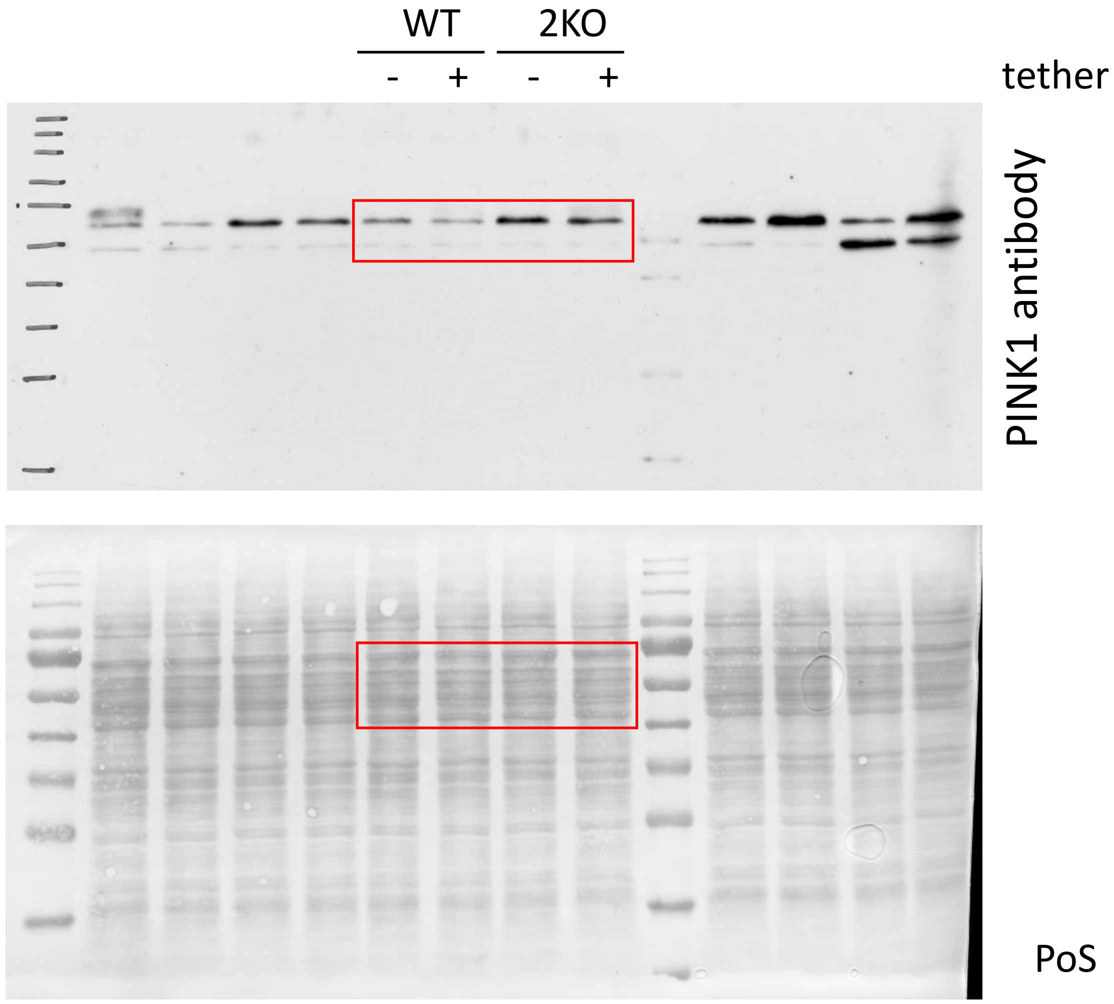

Fig. 3c

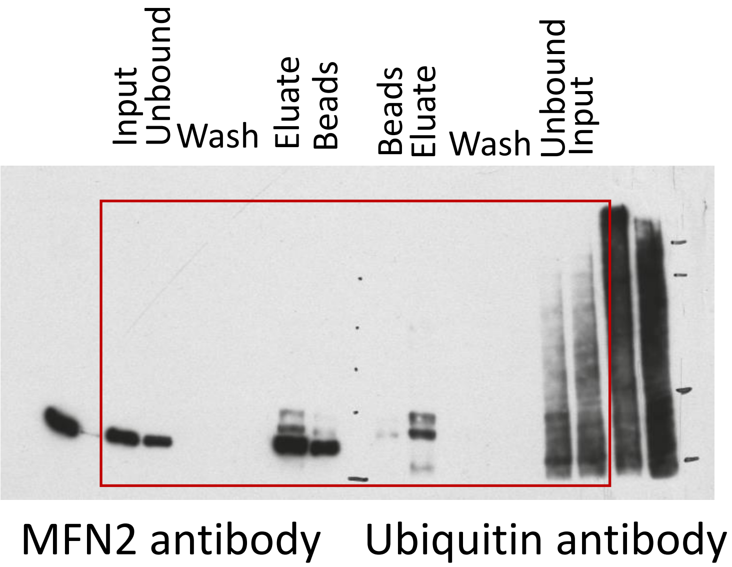
Input
Unbound
Wash
Eluate
Beads
Beads
Eluate
Wash
Unbound
Input
MFN2 antibody
Ubiquitin antibody

Fig. 3d

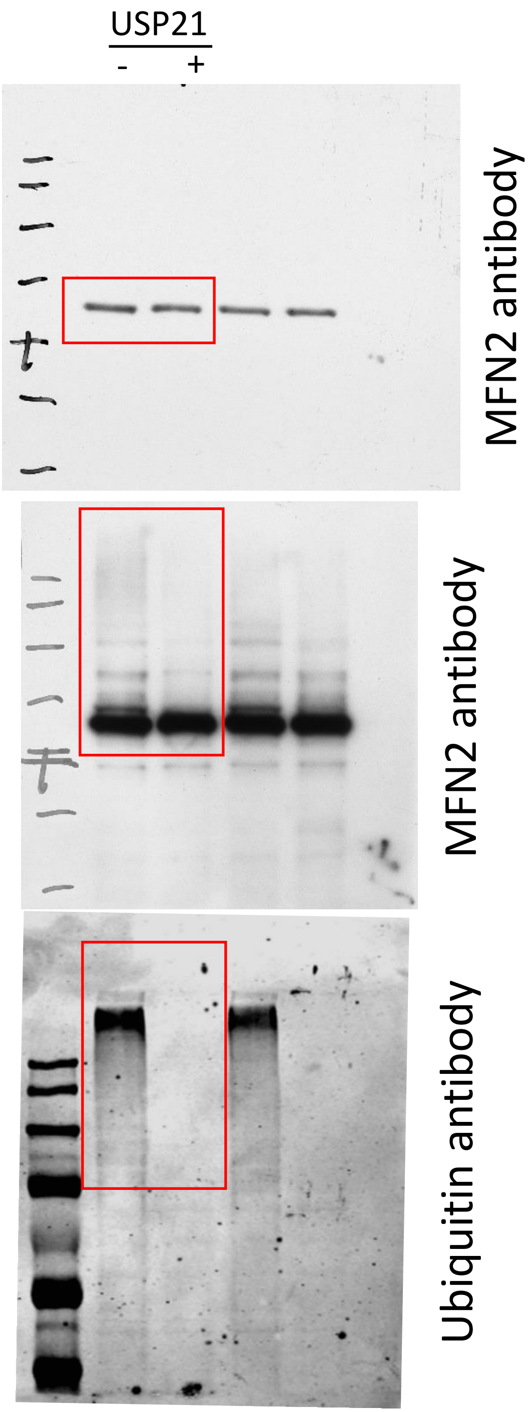
USP21
-
+
MFN2 antibody
MFN2 antibody
Ubiquitin antibody

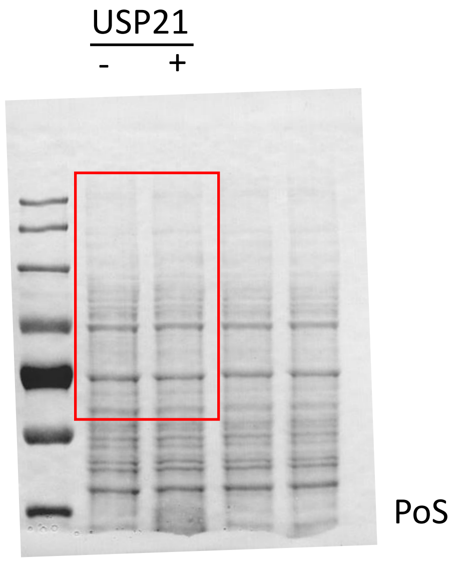
USP21
-
+
PoS

Fig. 3e

*MFN2 was was the first decorated but as the signal was saturated we used the signal still present on the second decaration, done with ubiquitin

*previous decoration

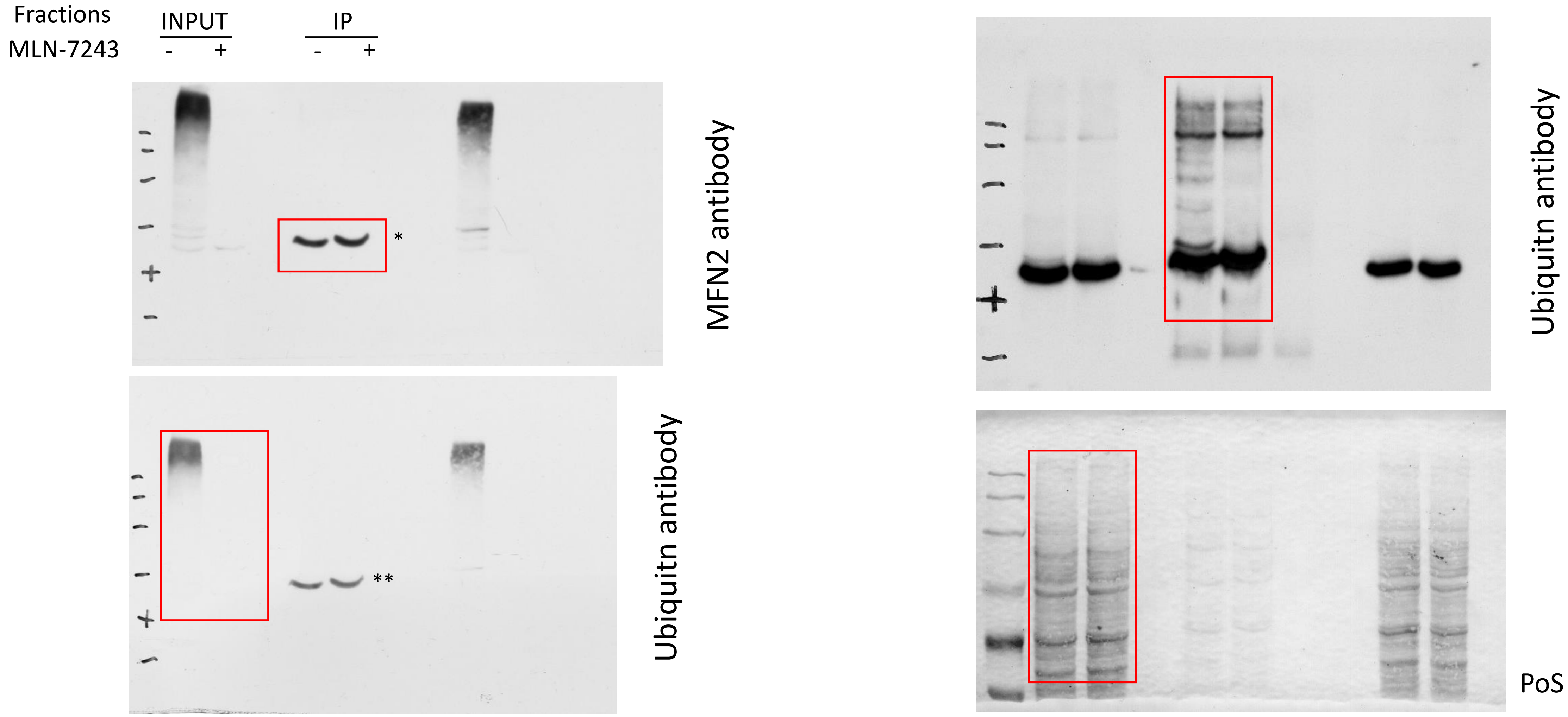

Fig. 4a

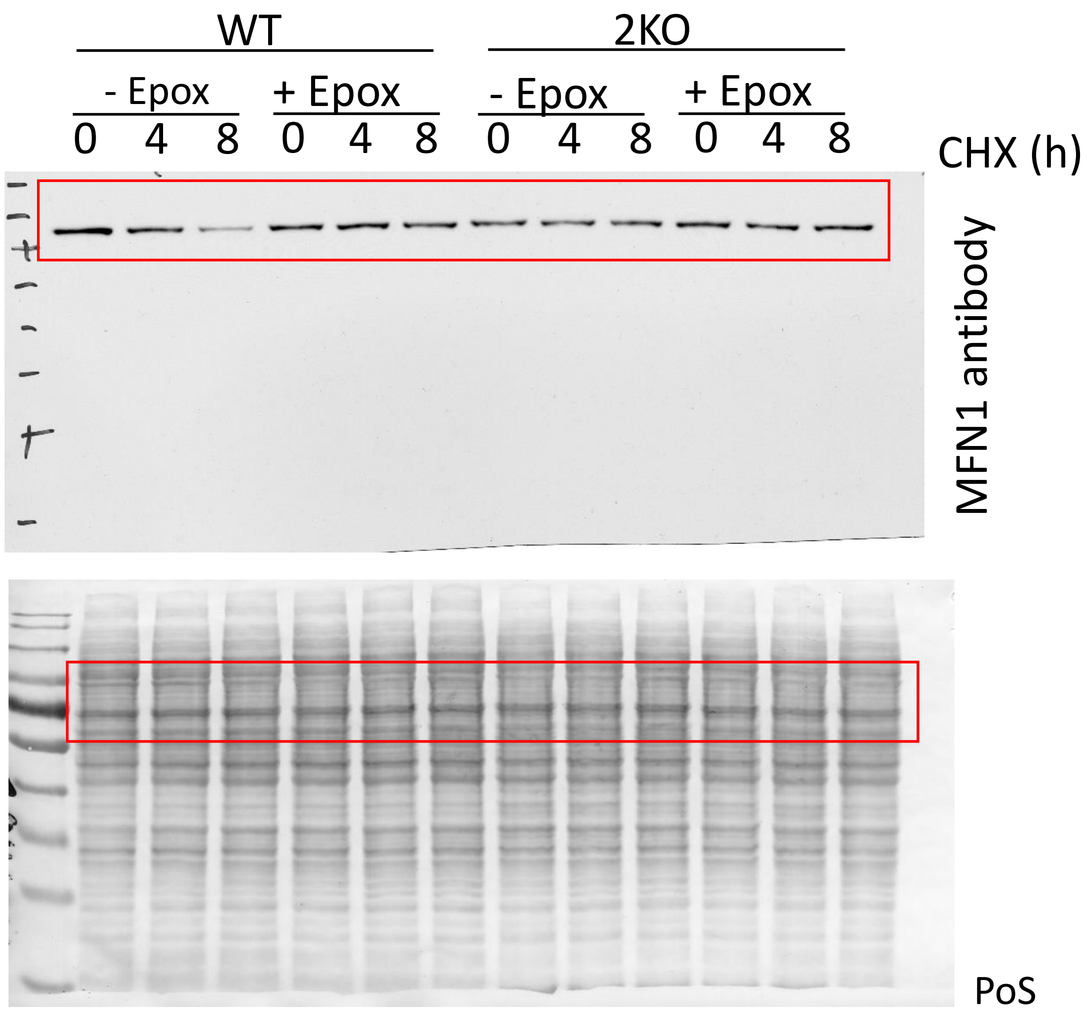
WT
2KO
- Epox
+ Epox
- Epox
+ Epox
0 4 8 0 4 8 0 4 8 0 4 8
CHX (h)
MFN1 antibody
PoS

Fig. 4b

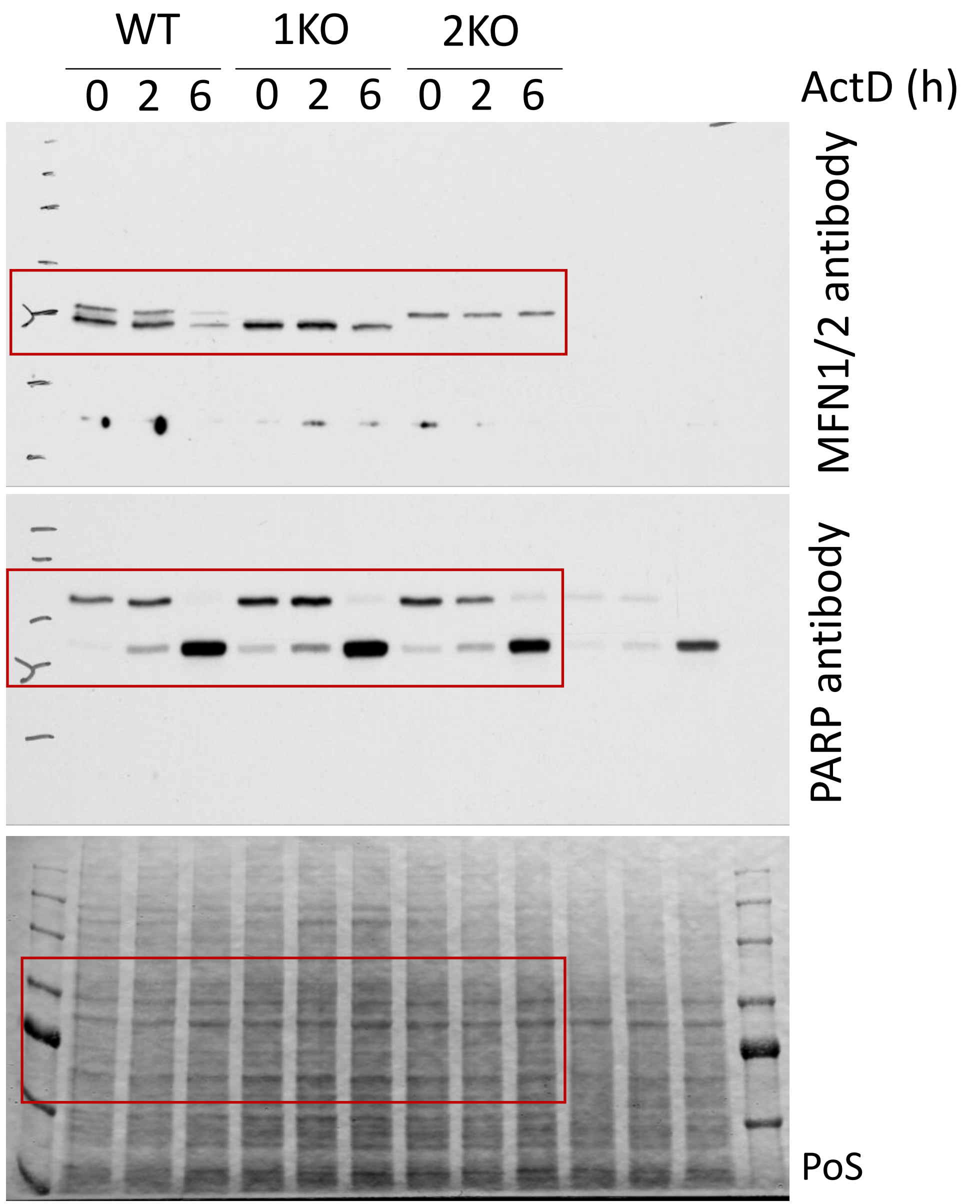
WT
1KO
2KO
0 2 6 0 2 6 0 2 6
ActD (h)
MFN1/2 antibody
PARP antibody
PoS

Fig. 4c

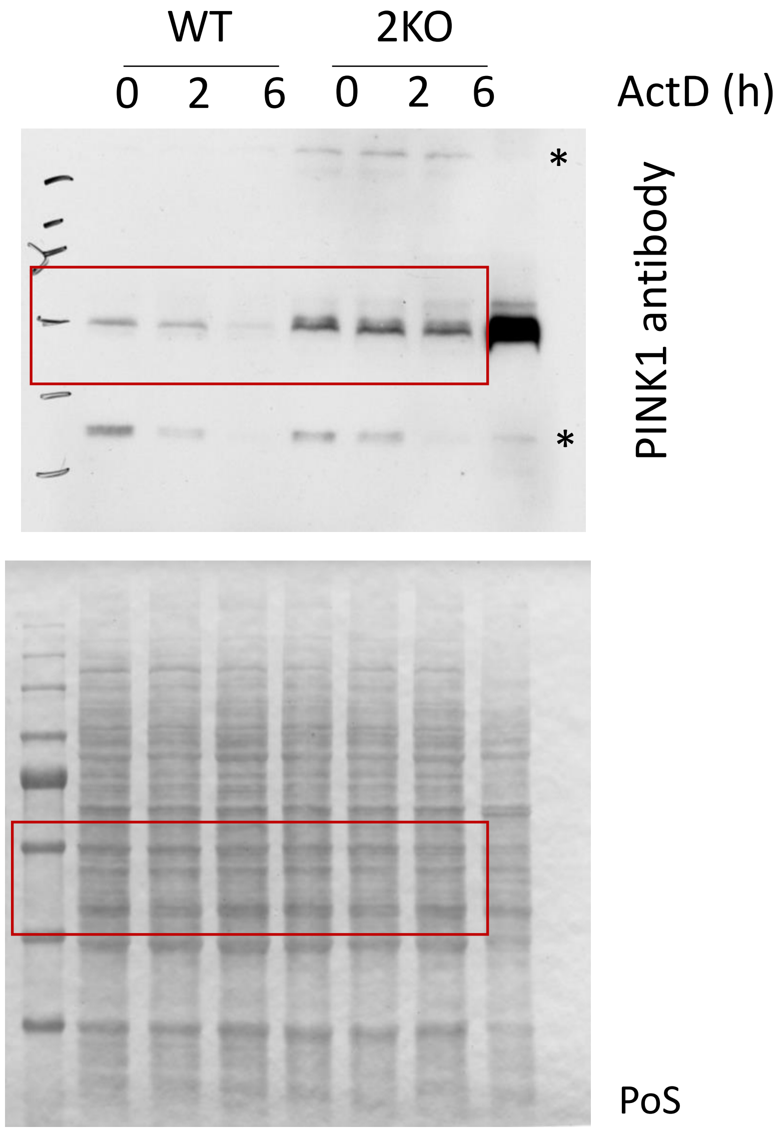

* cross-reaction

Fig. 4d

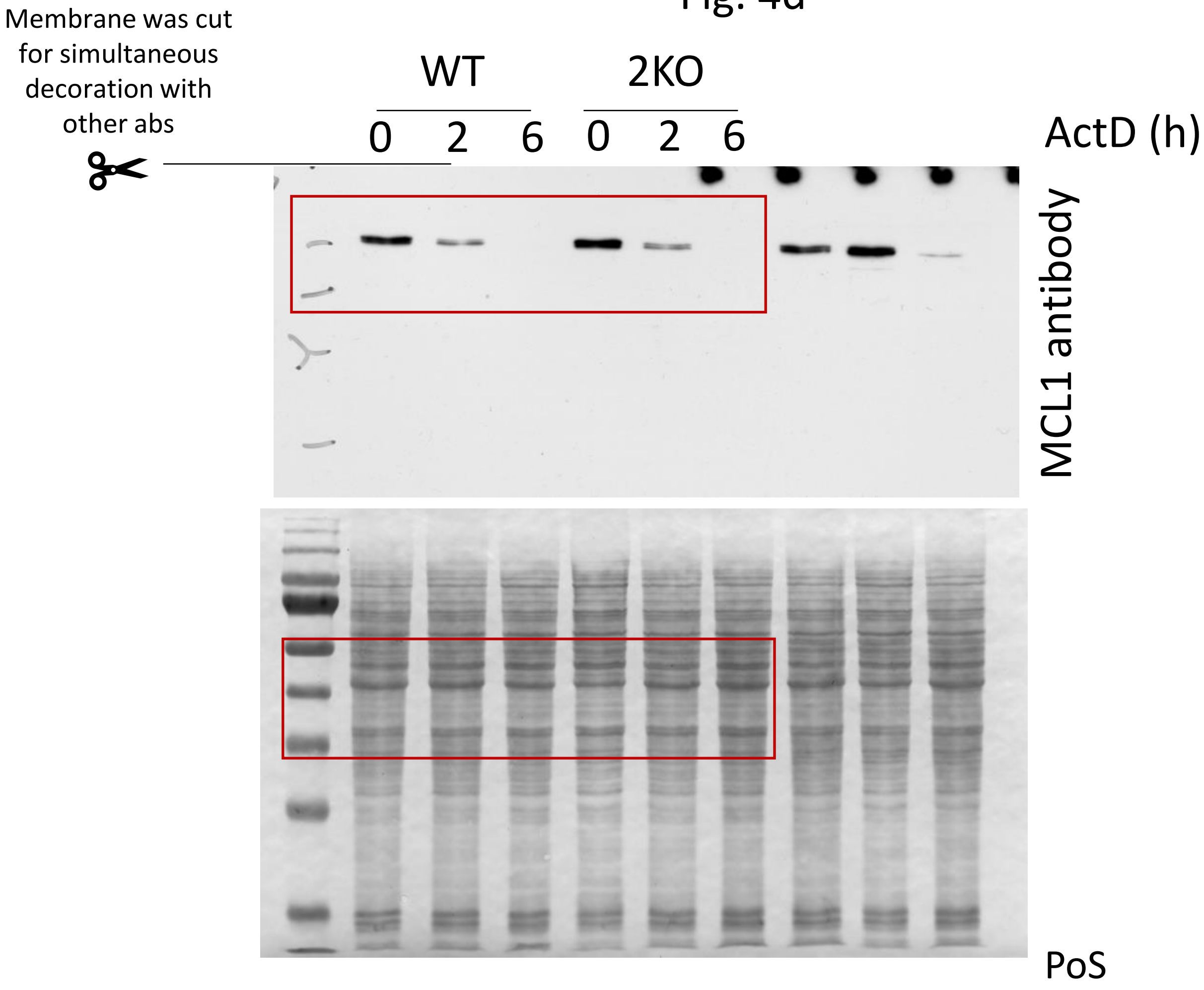

Source Data Supplementary Figures

Sup. 1a

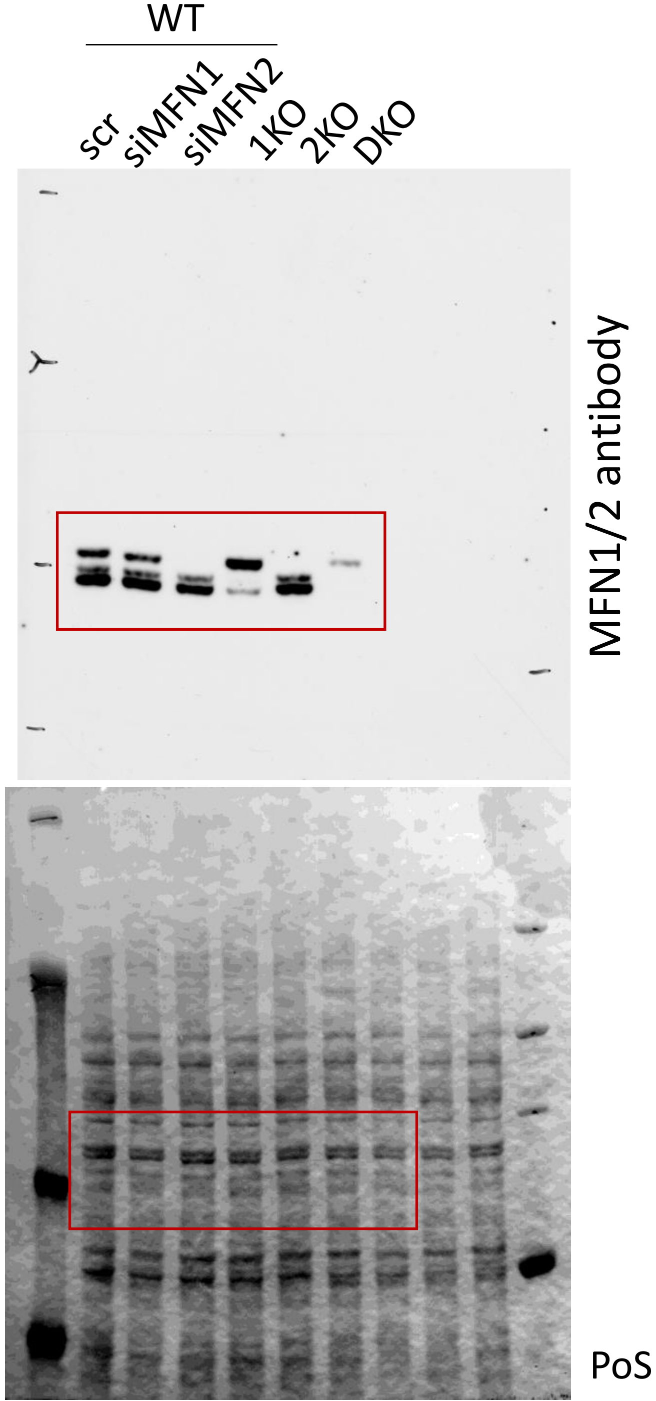

Sup. 1d

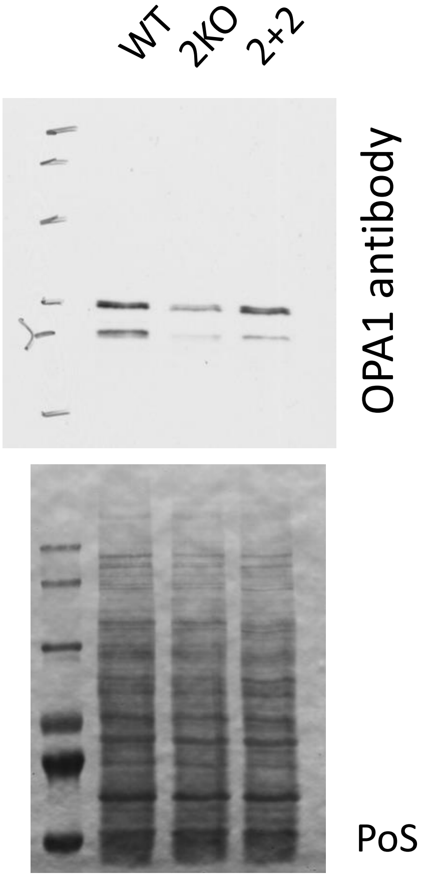

Sup. 1e

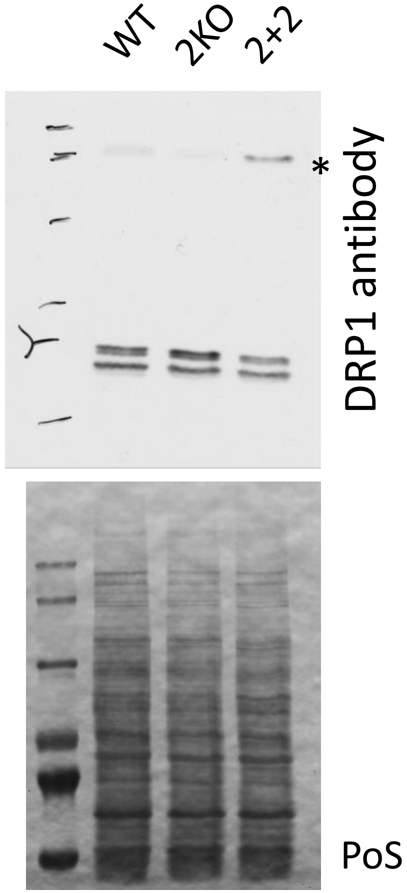

*previous decoration

Sup. 2a

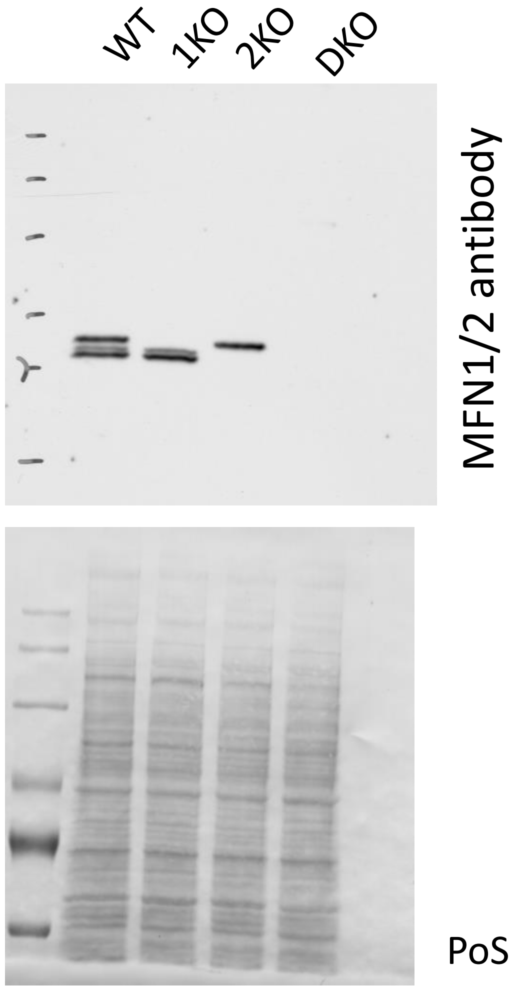

Sup. 4a

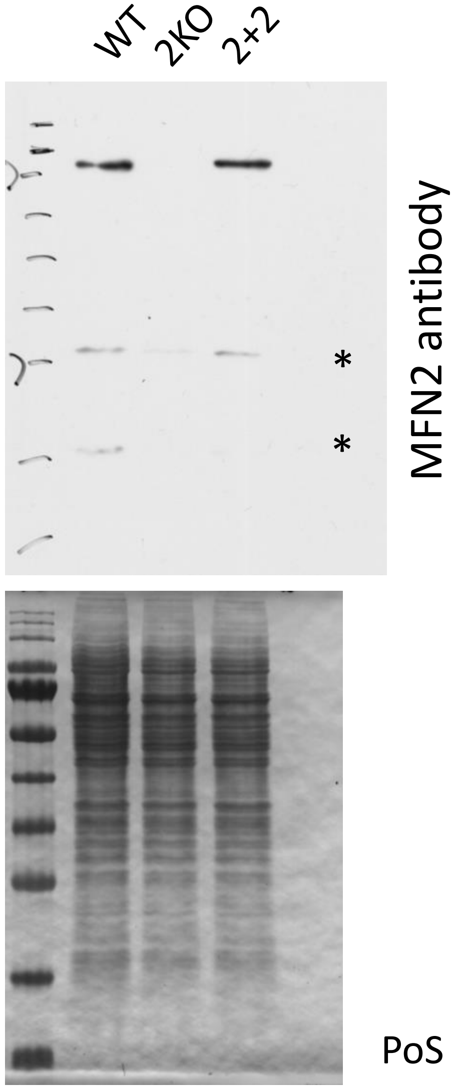

*previous decoration

Sup. 5b

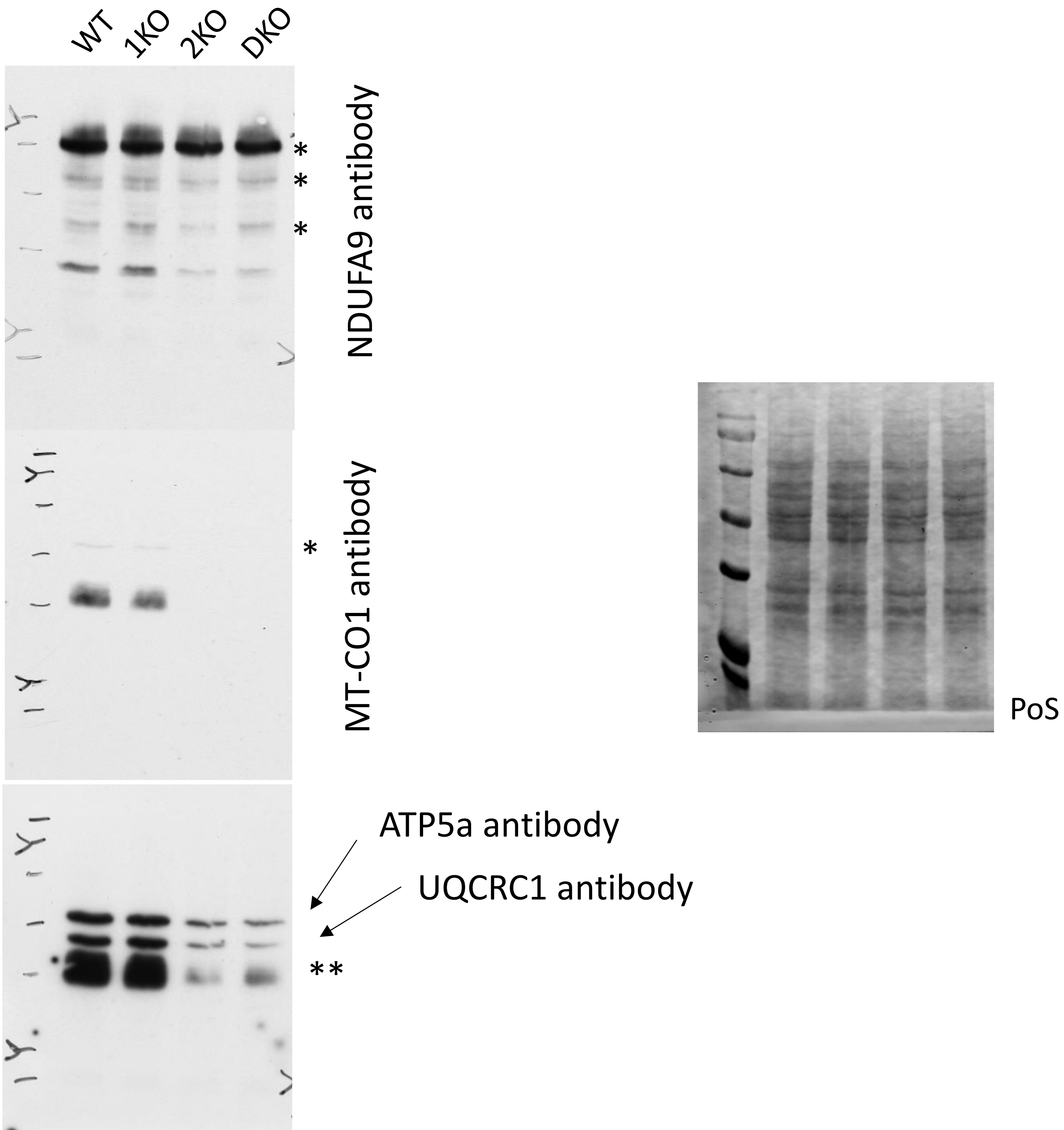

*cross-reaction
** previous decoration

Sup. 5b

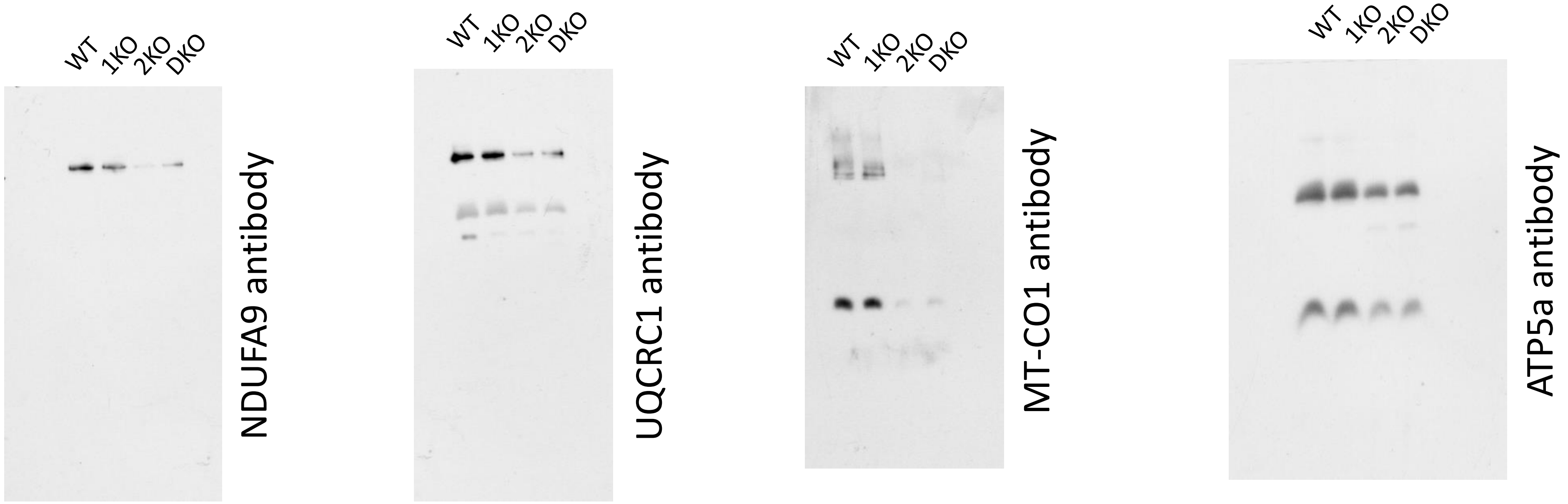
WT
1KO
2KO
DKO
NDUFA9 antibody
UQCRC1 antibody
MT-CO1 antibody
ATP5a antibody

Sup. 5c

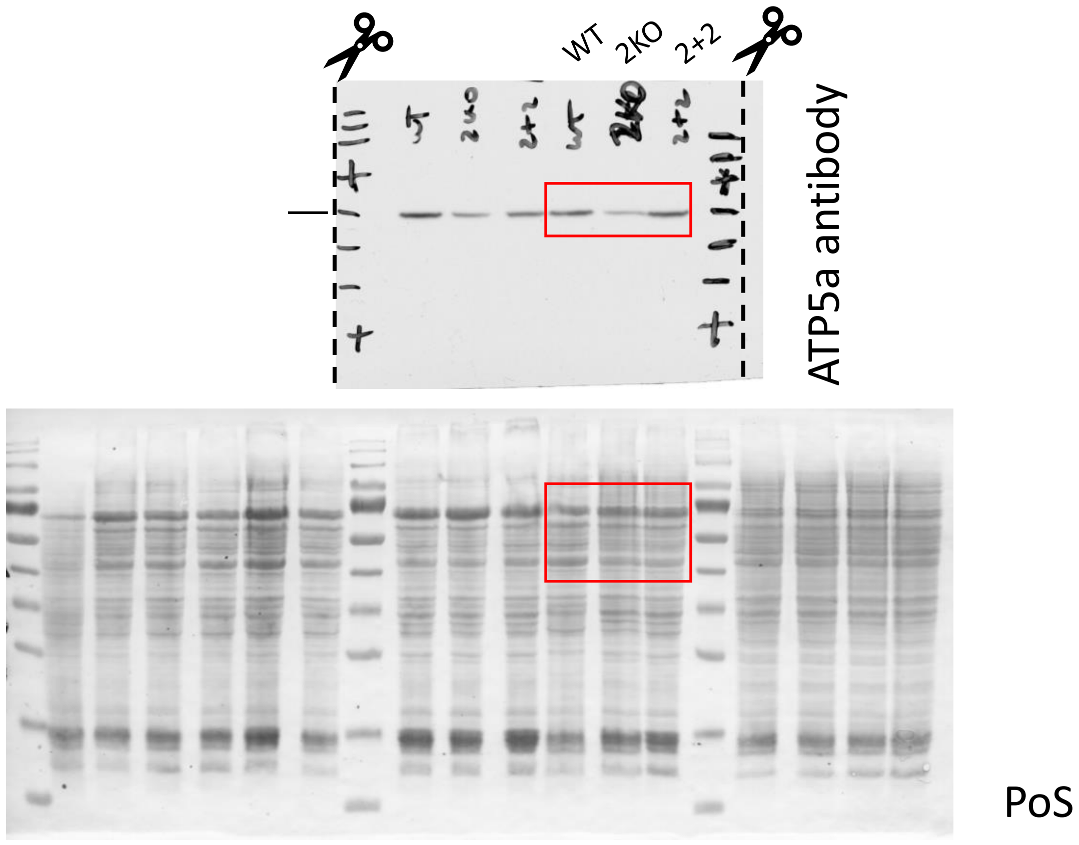

Sup. 6a

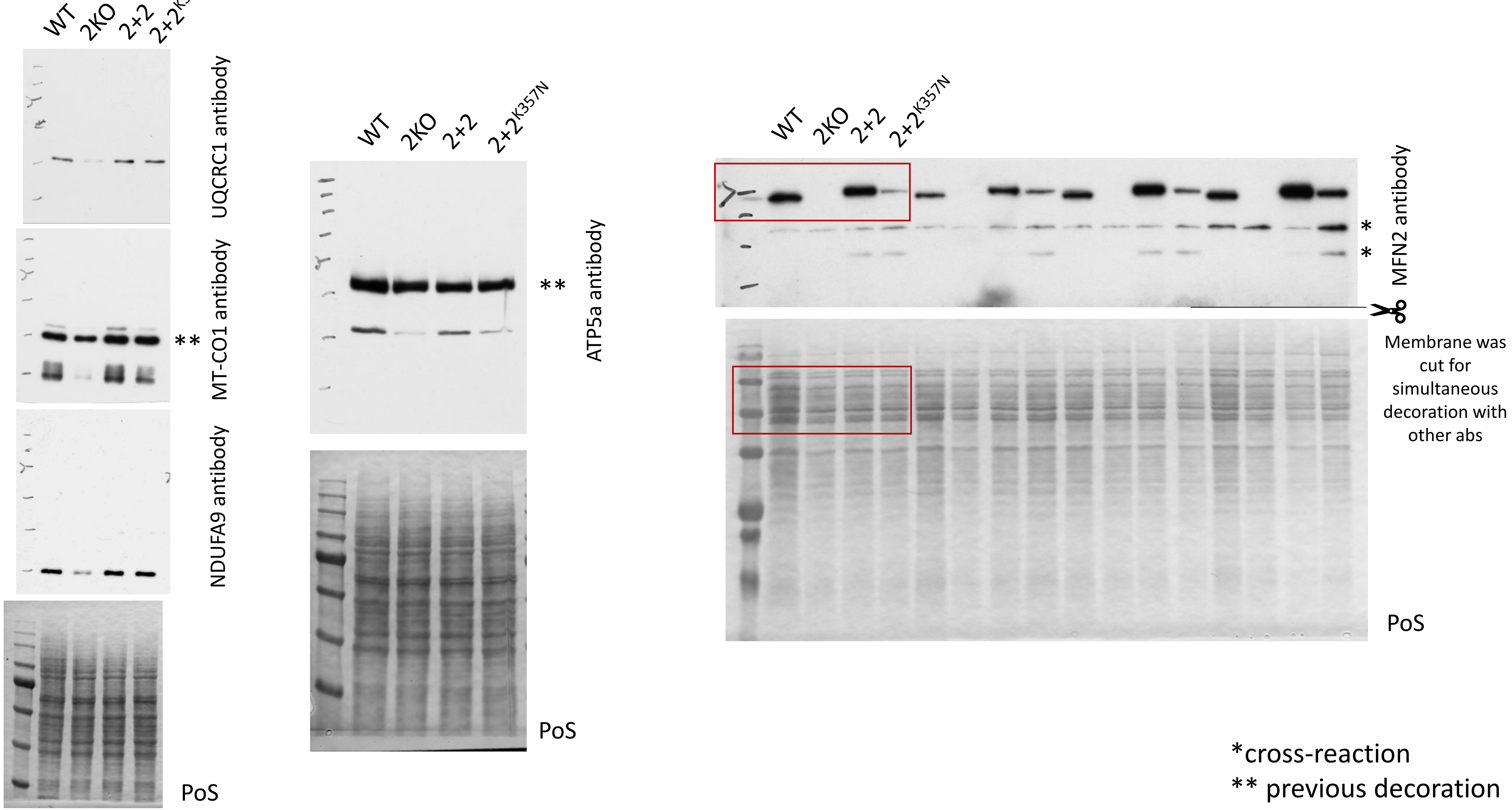
WT
2KO
2+2
2+2K357N
UQCRC1 antibody
MT-CO1 antibody
**
NDUFA9 antibody
PoS
ATP5a antibody
**
PoS
MFN2 antibody
*
*
Membrane was cut for simultaneous decoration with other abs
PoS
*cross-reaction
** previous decoration

Sup. 6b

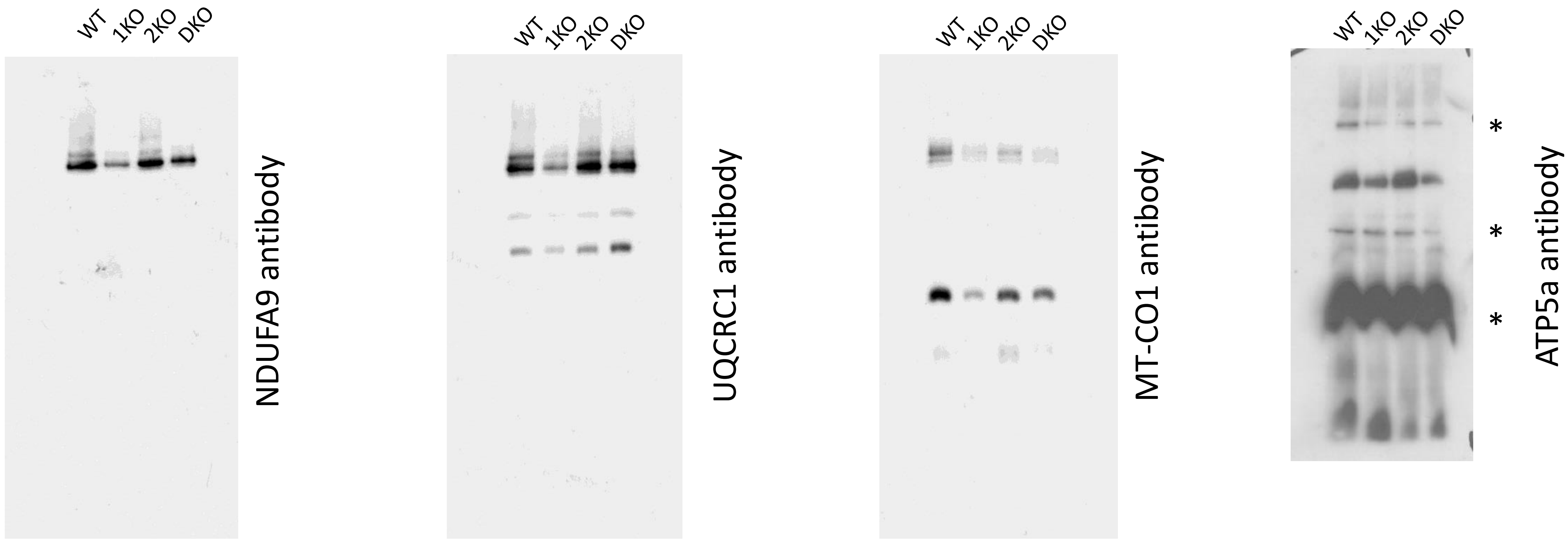

* Previous decoration

Sup. 6f

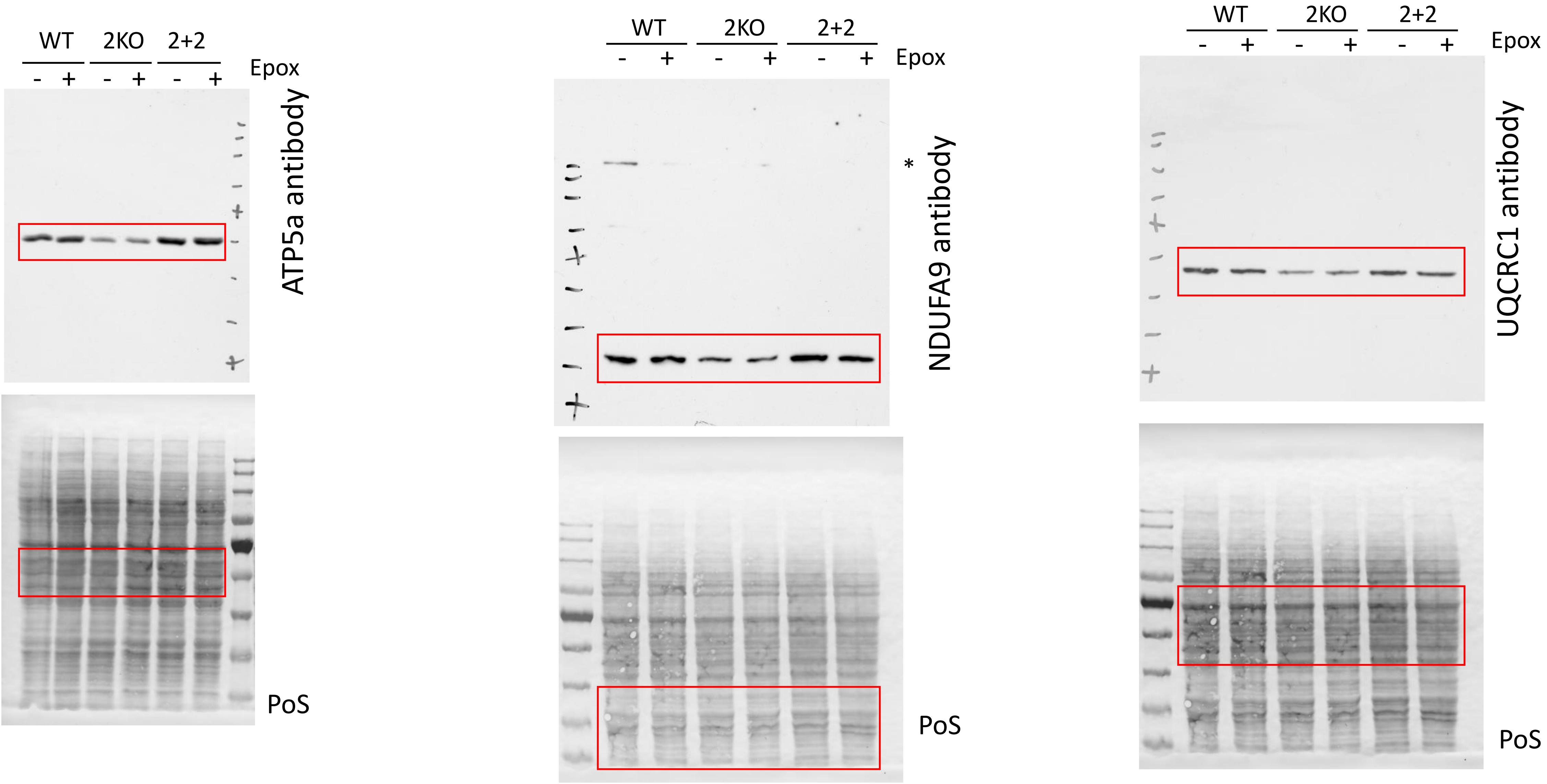

* cross-reaction

Sup. 7a

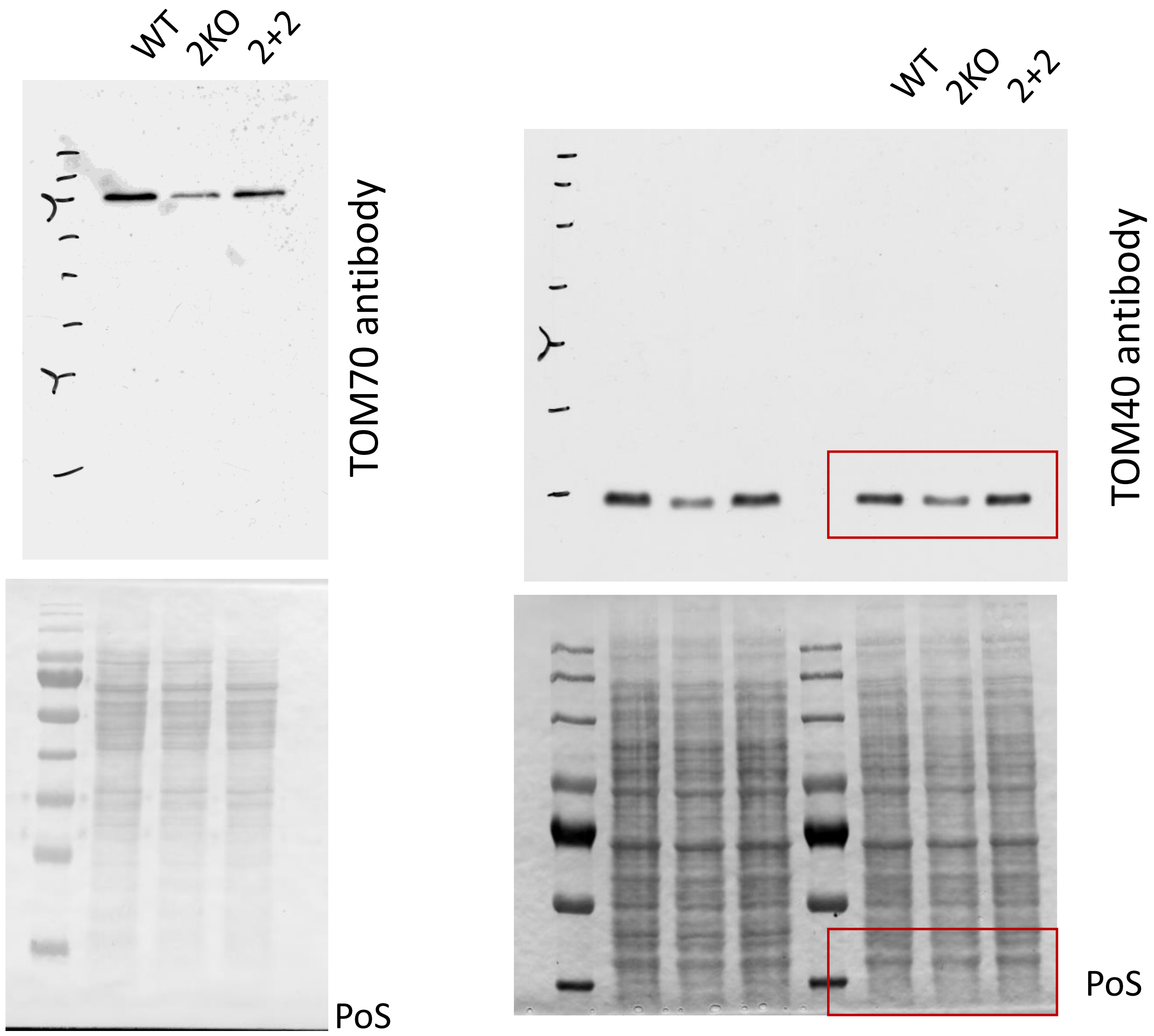
WT
2KO
2+2
TOM70 antibody
PoS
WT
2KO
2+2
TOM40 antibody
PoS

Sup. 7b

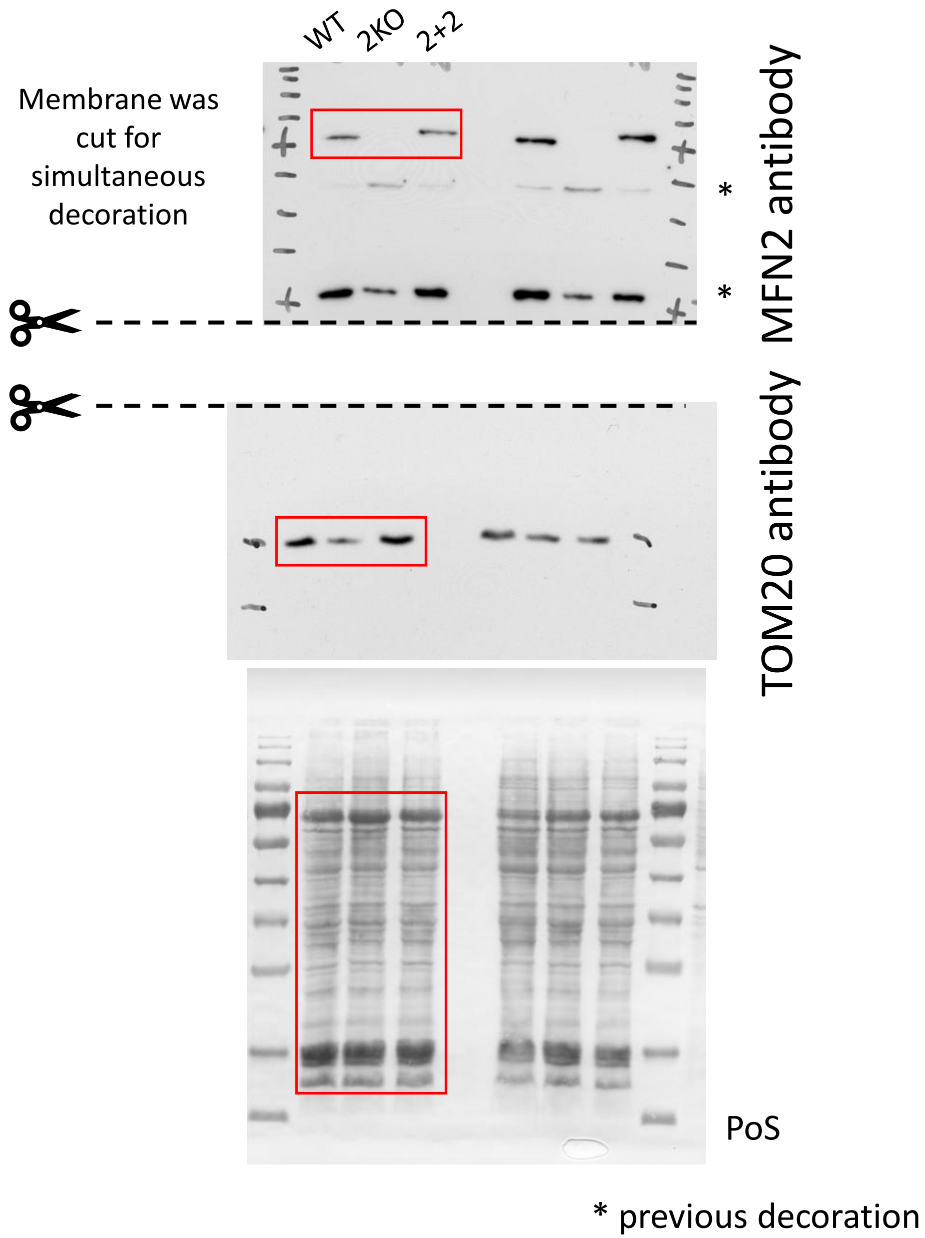
WT
2KO
2+2
Membrane was cut for simultaneous decoration
*
*
MFN2 antibody
TOM20 antibody
PoS
* previous decoration

Sup. 7c

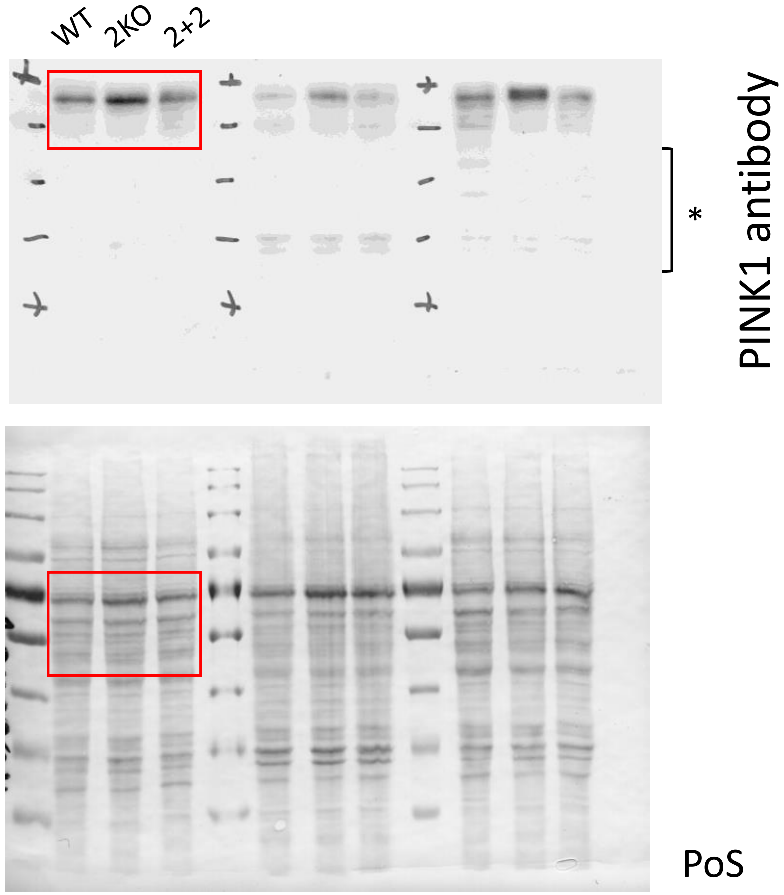

Sup. 7d

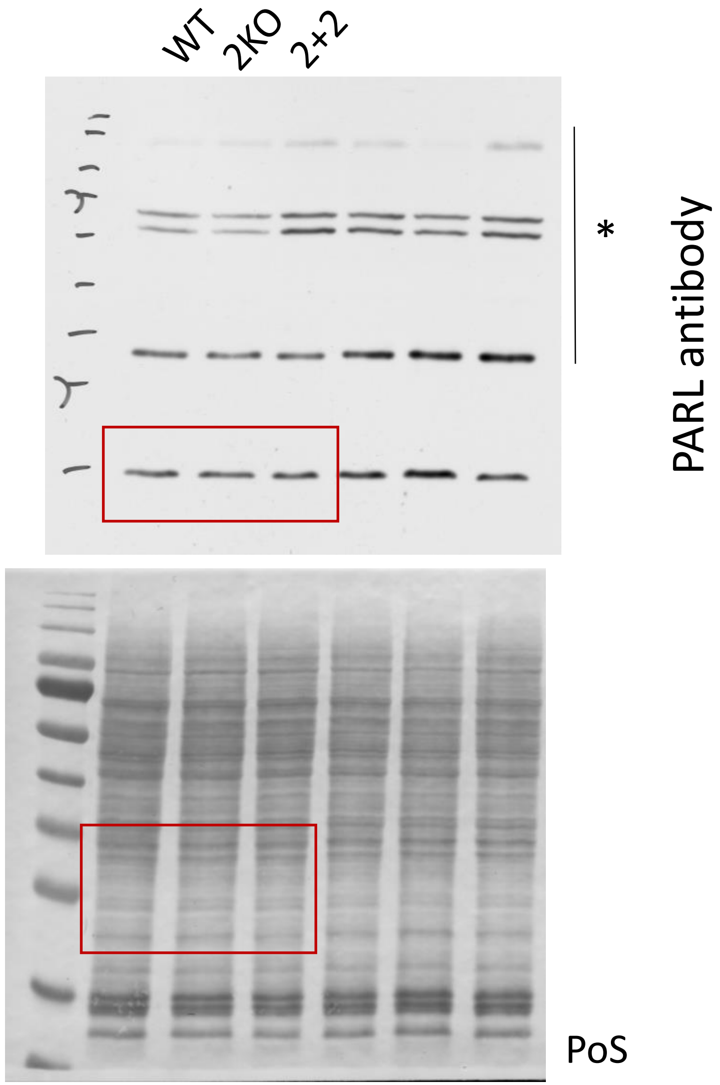

*cross-reaction

Sup. 7f

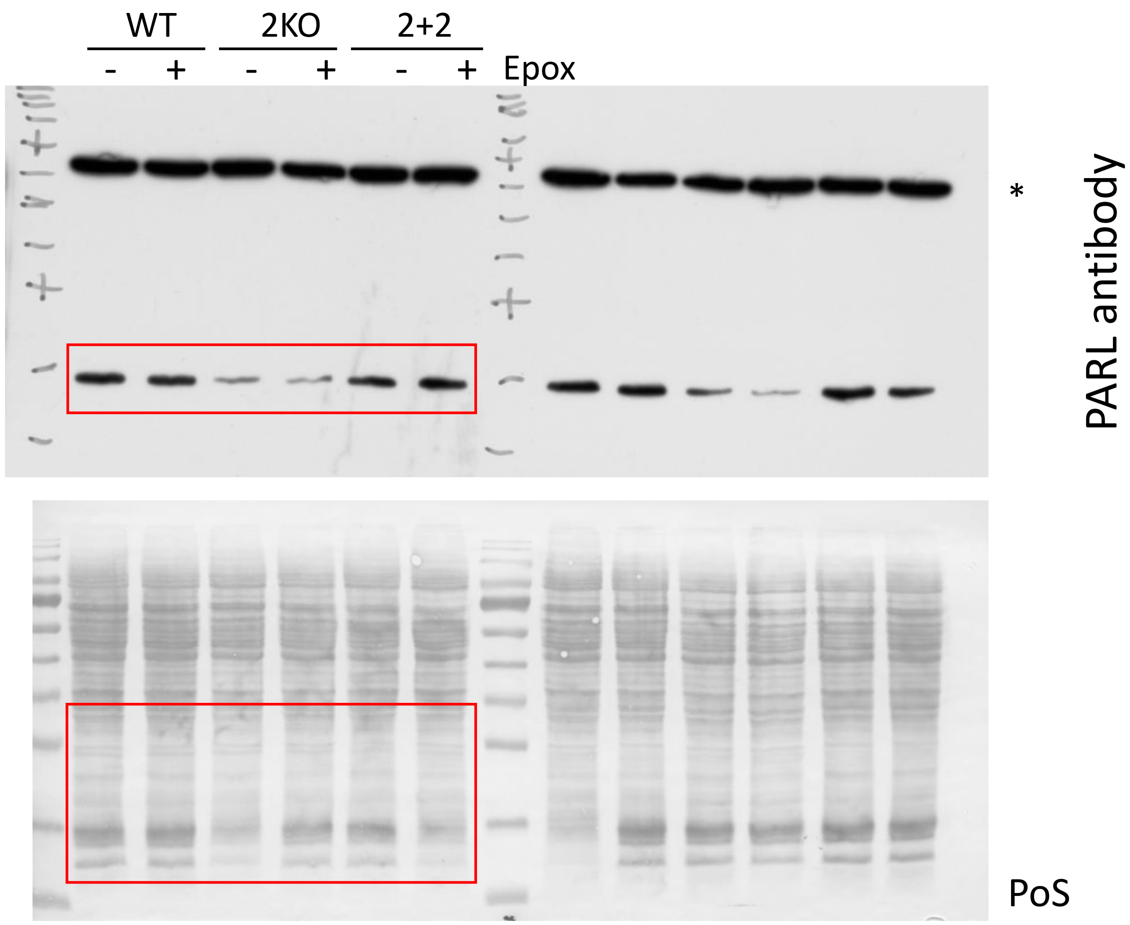

* cross-reaction

Sup. 7h

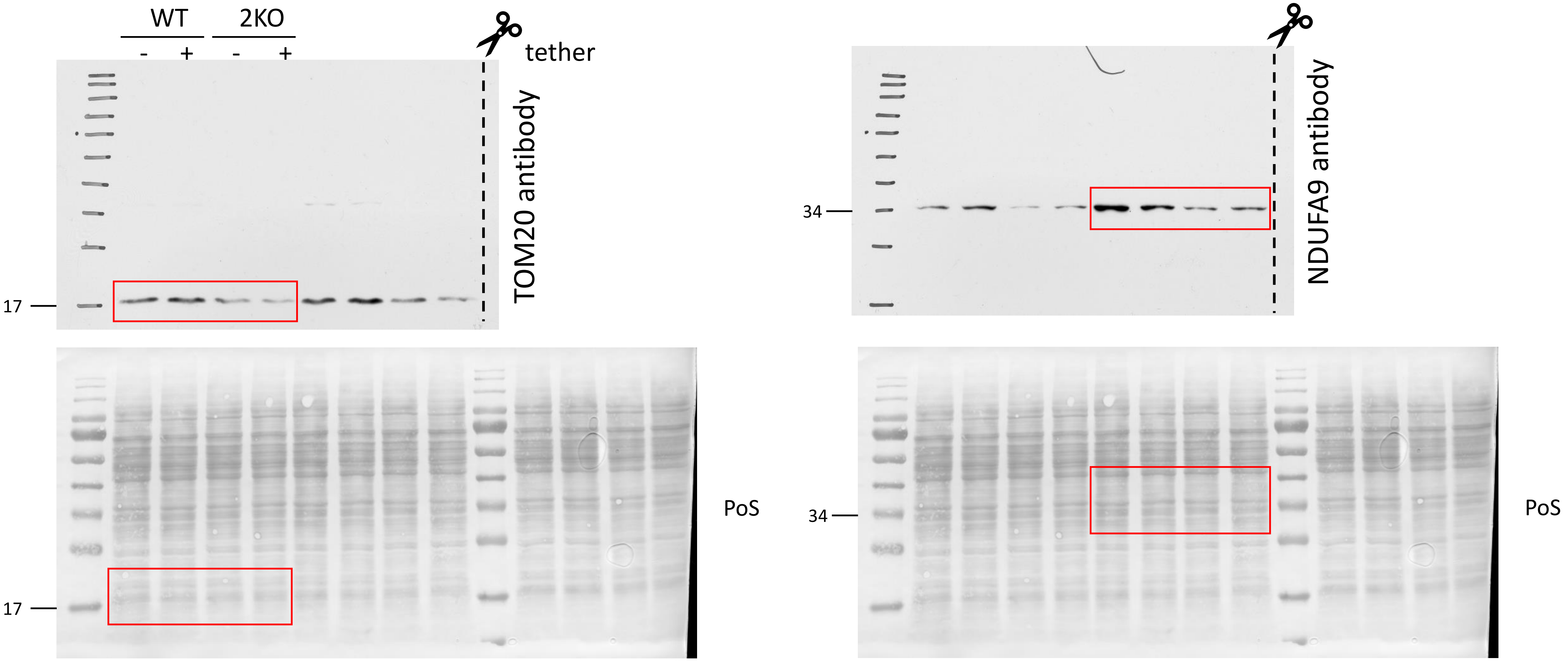

WT
2KO
- + - +
tether
TOM20 antibody
17
NDUFA9 antibody
34
PoS
17
34
PoS

Sup. 7h - continuation

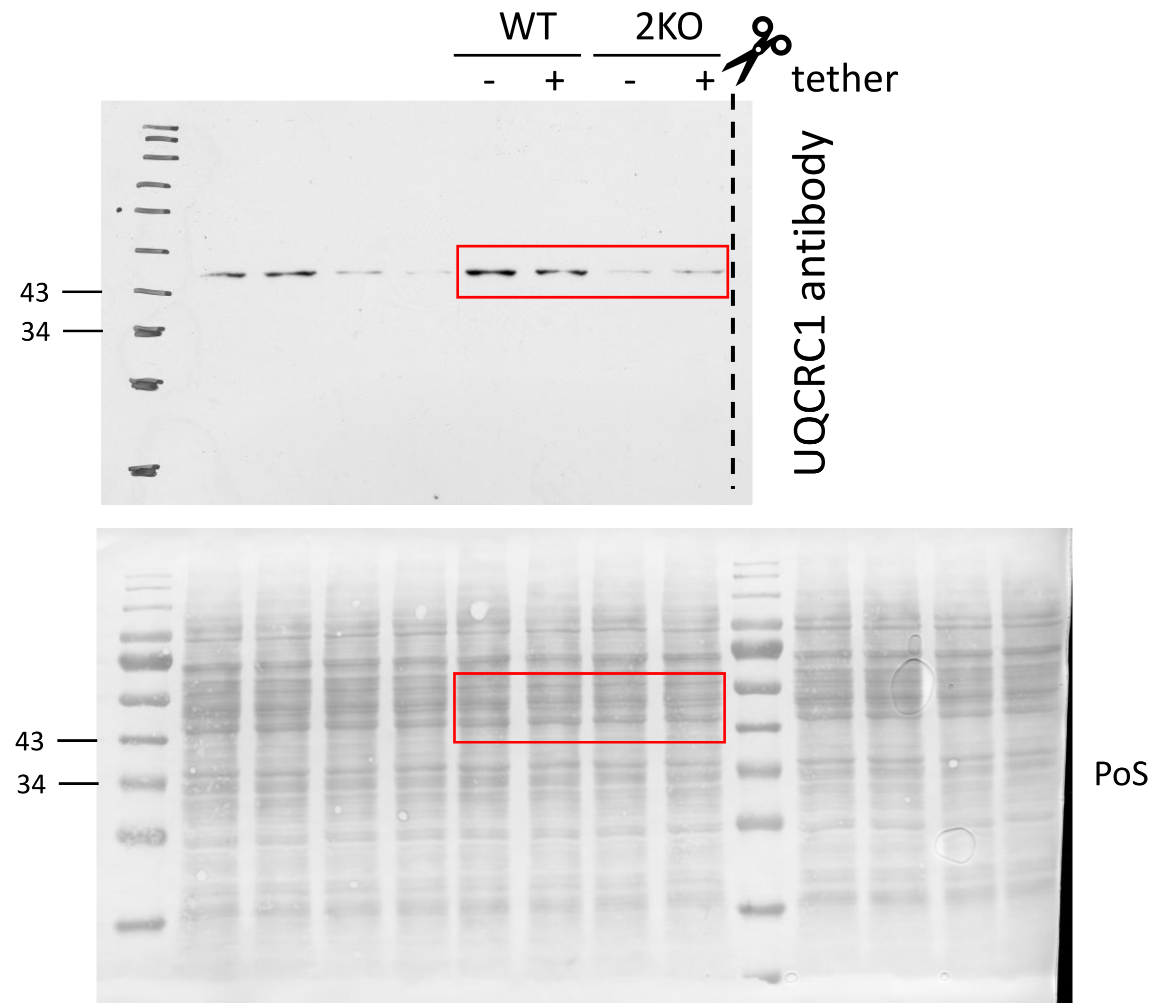

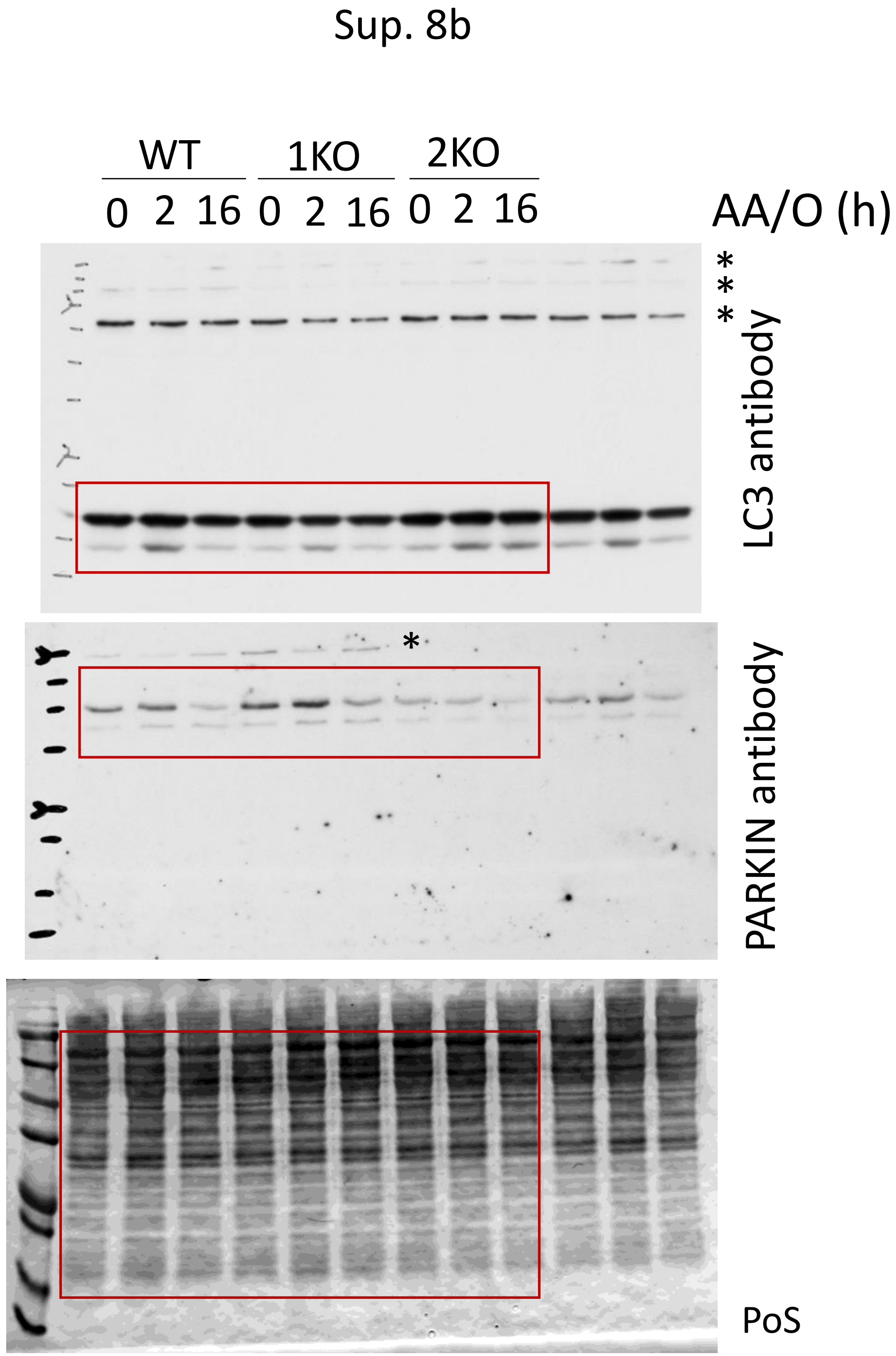
Sup. 8b
WT
1KO
2KO
0 2 16 0 2 16 0 2 16
AA/O (h)
***
LC3 antibody
*
PARKIN antibody
PoS

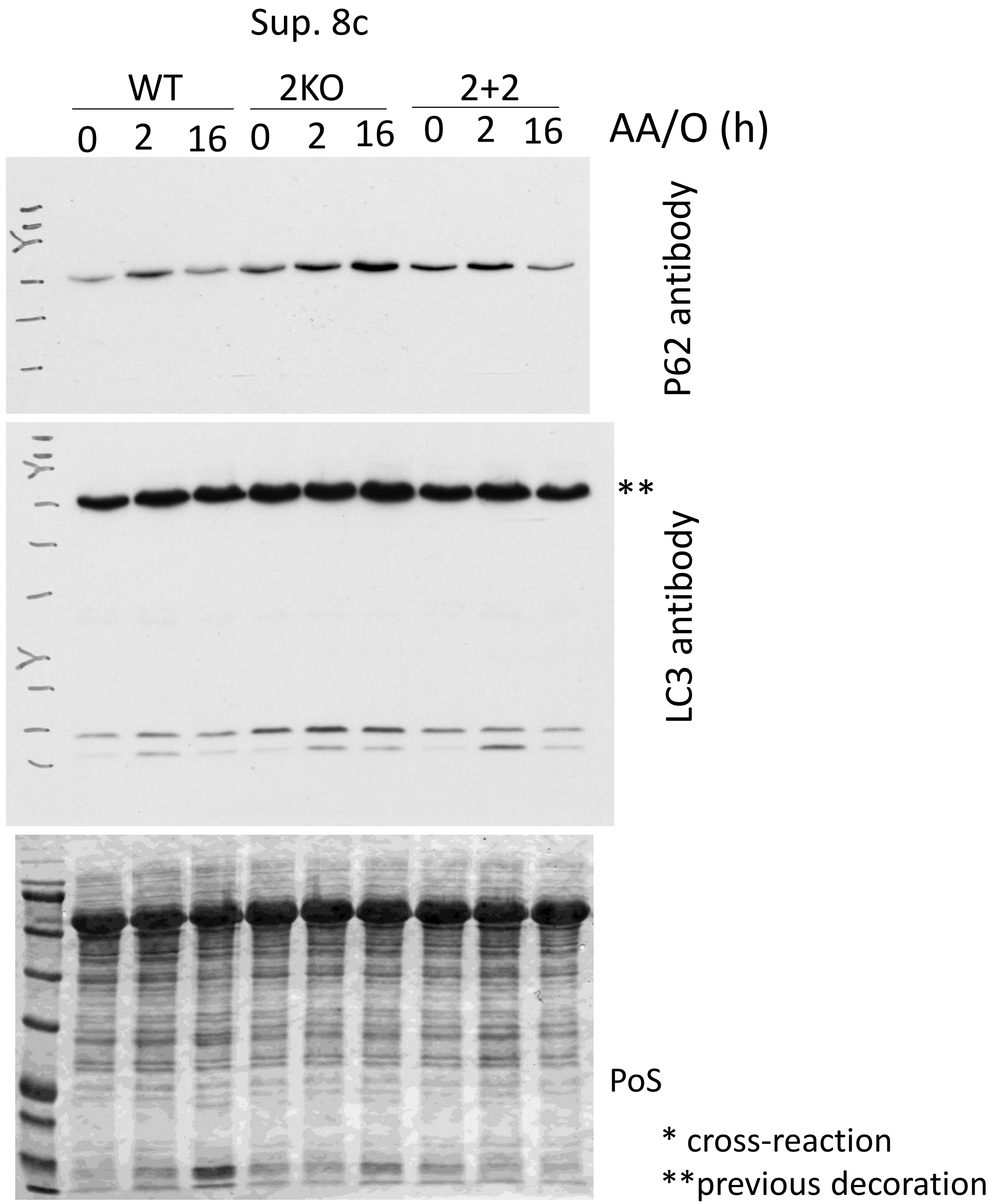
Sup. 8c
WT
2KO
2+2
0 2 16 0 2 16 0 2 16
AA/O (h)
P62 antibody
**
LC3 antibody
PoS
* cross-reaction
**previous decoration

Sup. 9a

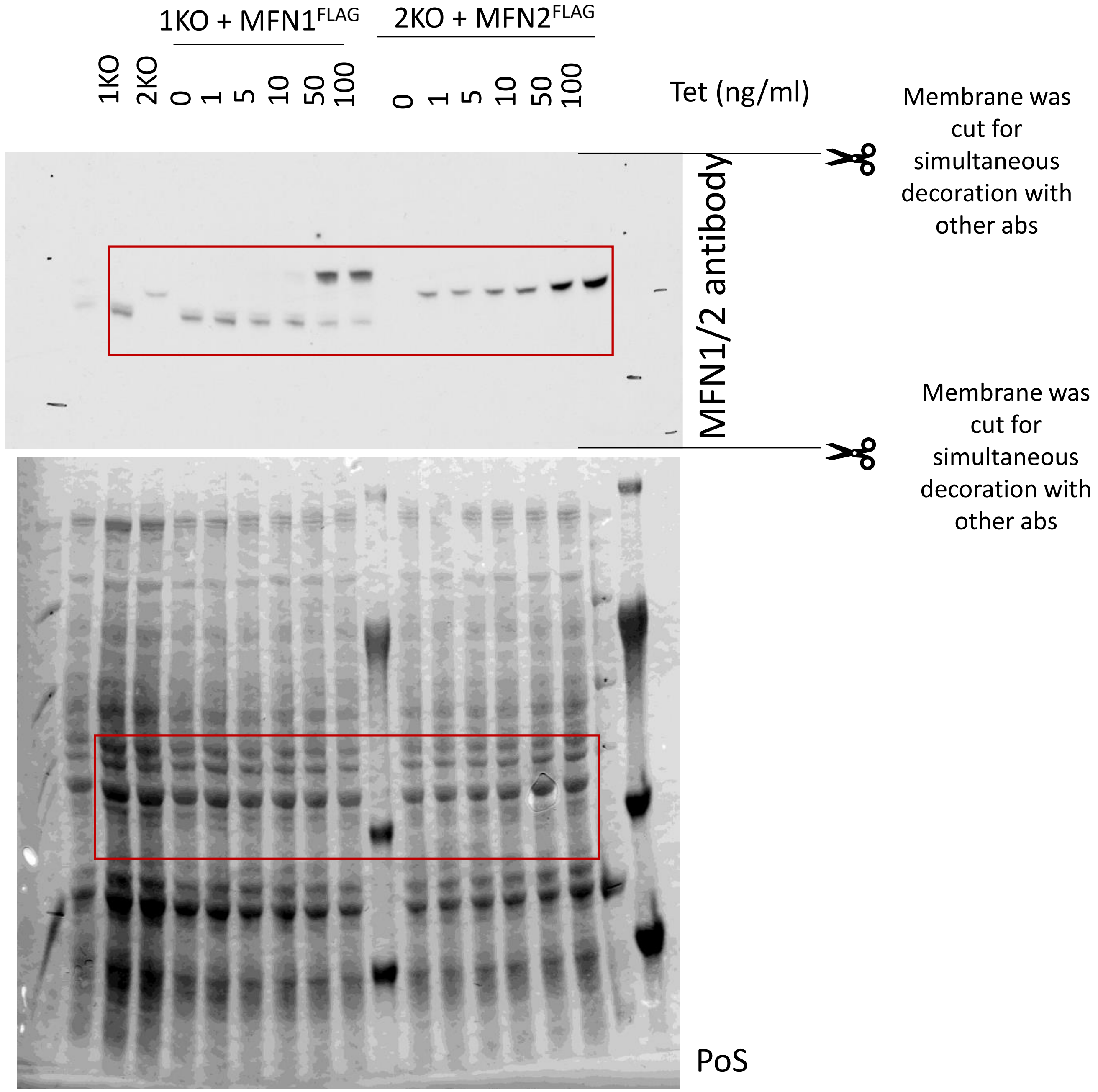
1KO + MFN1FLAG
2KO + MFN2FLAG
1KO
2KO
0
1
5
10
50
100
0
1
5
10
50
100
Tet (ng/ml)
Membrane was cut for simultaneous decoration with other abs
MFN1/2 antibody
Membrane was cut for simultaneous decoration with other abs
PoS

Sup. 10c

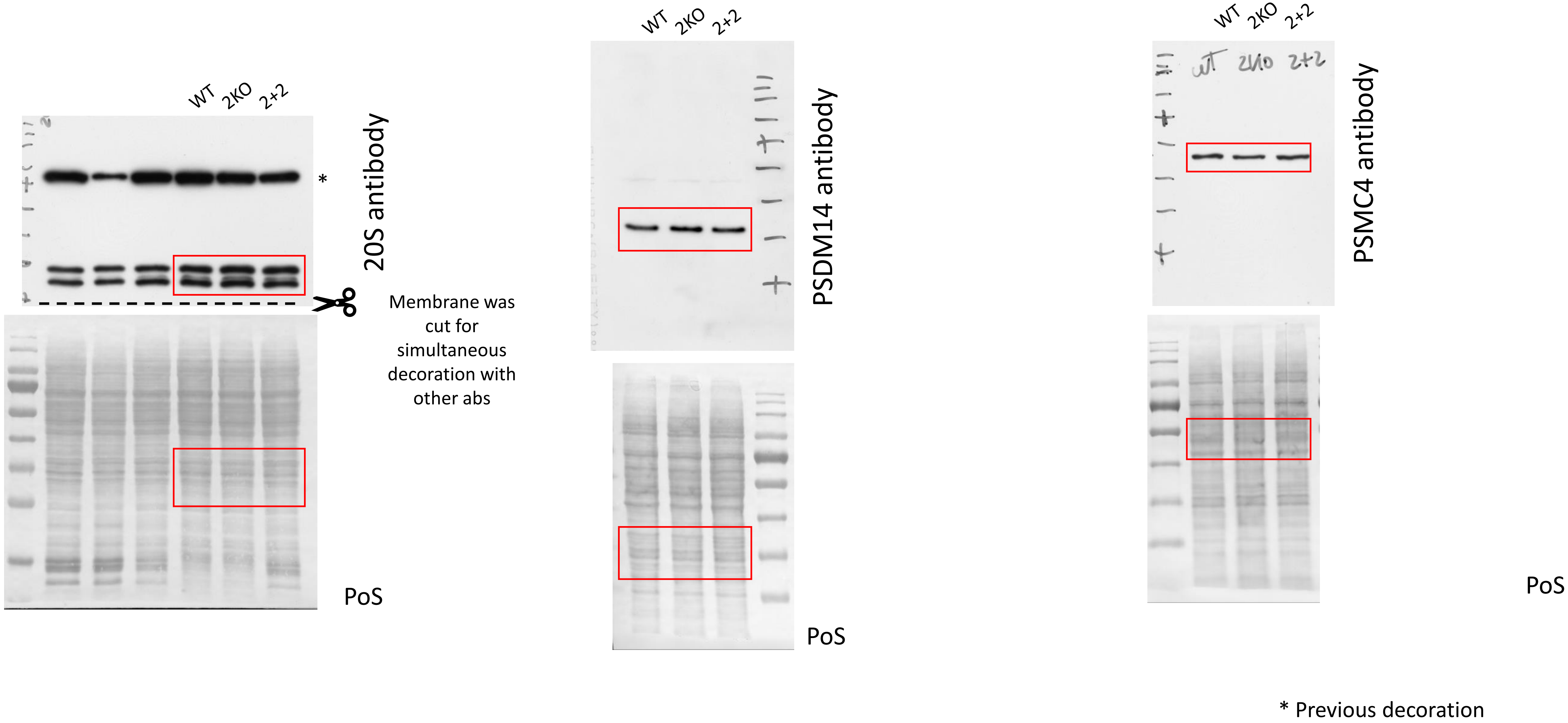
WT
2KO
2+2
20S antibody
*
Membrane was cut for simultaneous decoration with other abs
PoS
WT
2KO
2+2
PSDM14 antibody
PoS
WT
2KO
2+2
PSMC4 antibody
PoS
* Previous decoration

Sup. 10e

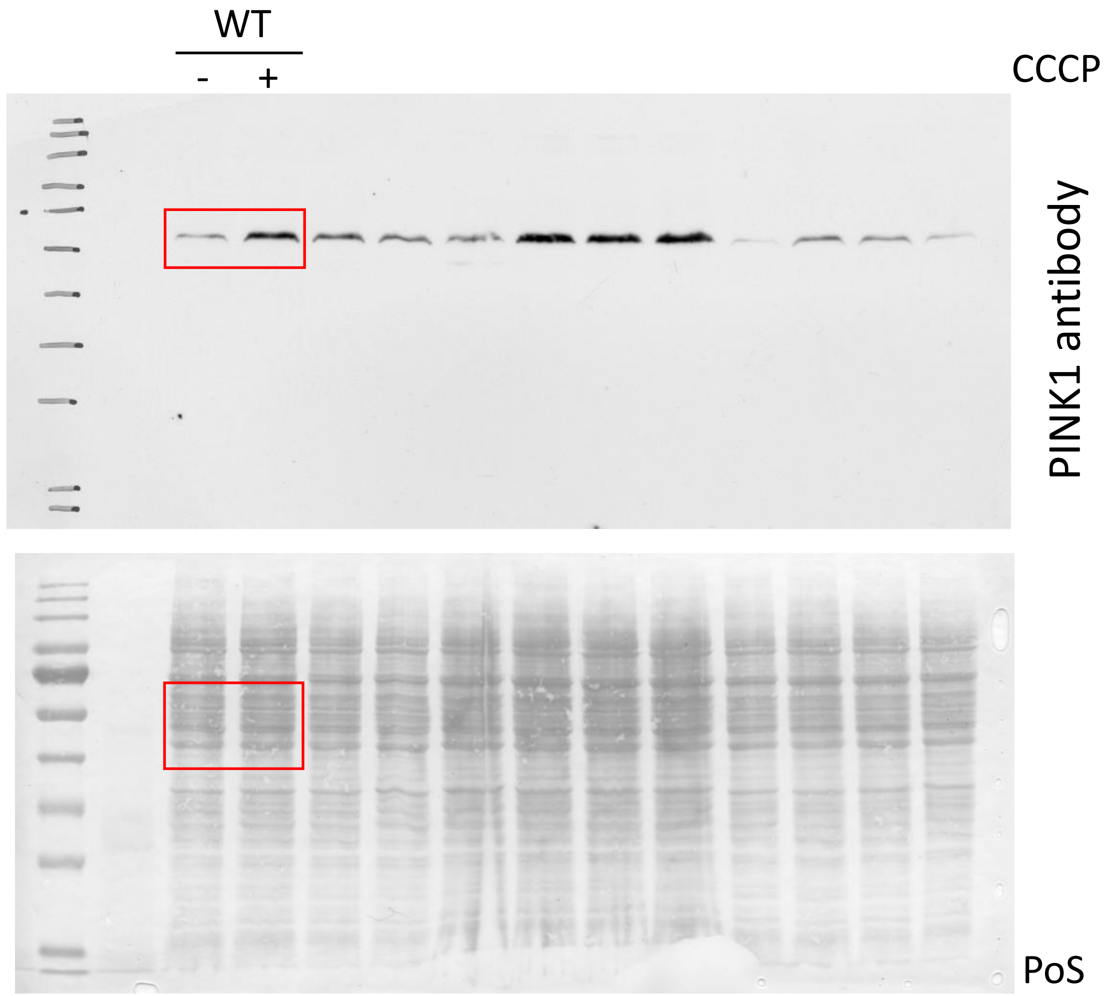

Sup. 10g

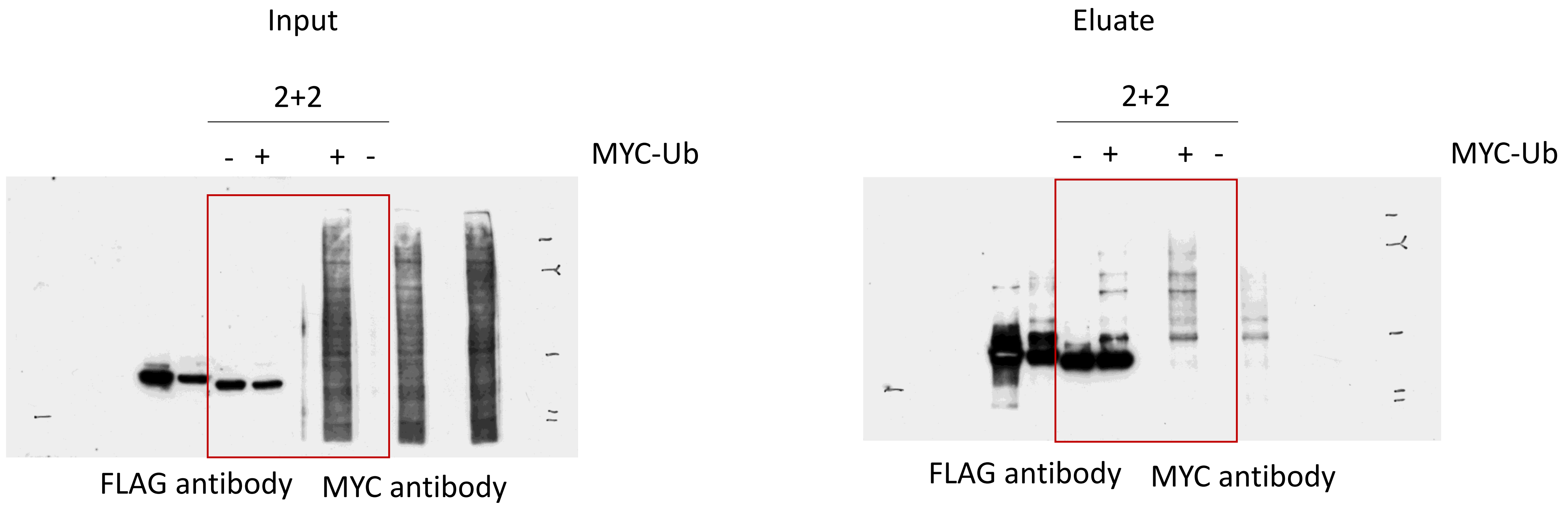

Input
2+2
- + + -
MYC-Ub
FLAG antibody
MYC antibody
Eluate
2+2
- + + -
MYC-Ub
FLAG antibody
MYC antibody

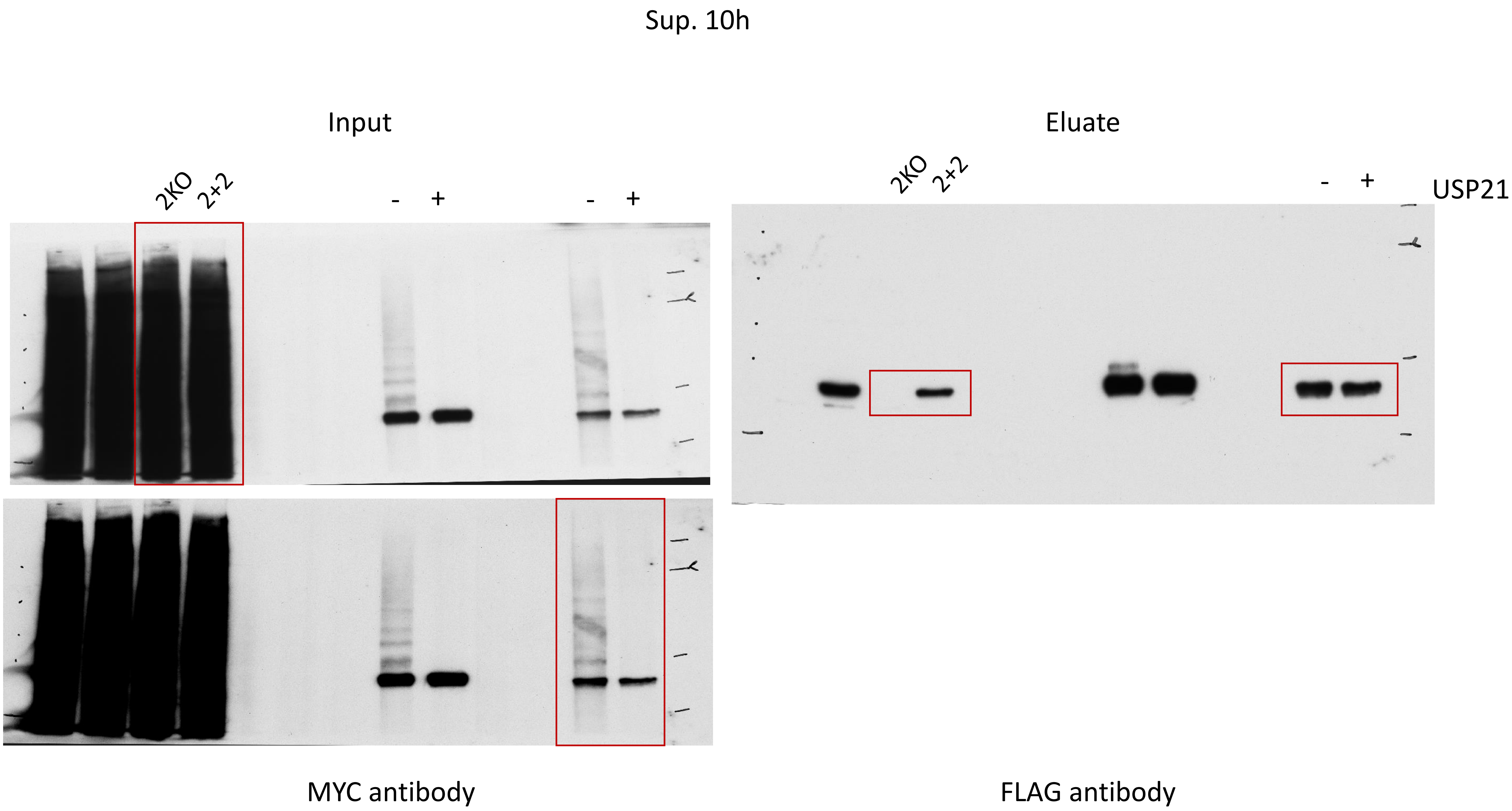
Sup. 10h
Input
Eluate
2KO
2+2
-
+
-
+
2KO
2+2
-
+
USP21
MYC antibody
FLAG antibody

Sup. 11a

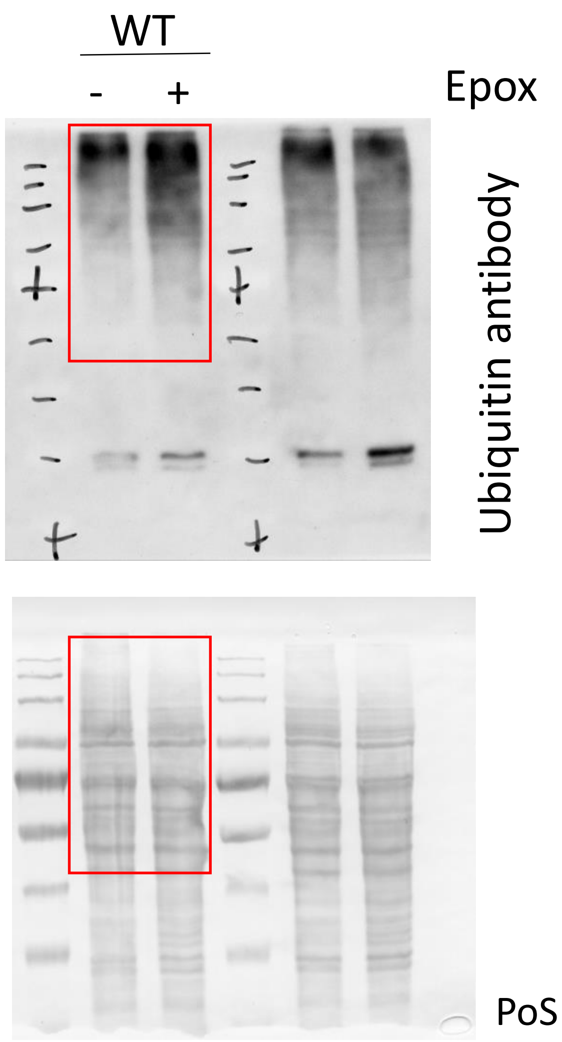

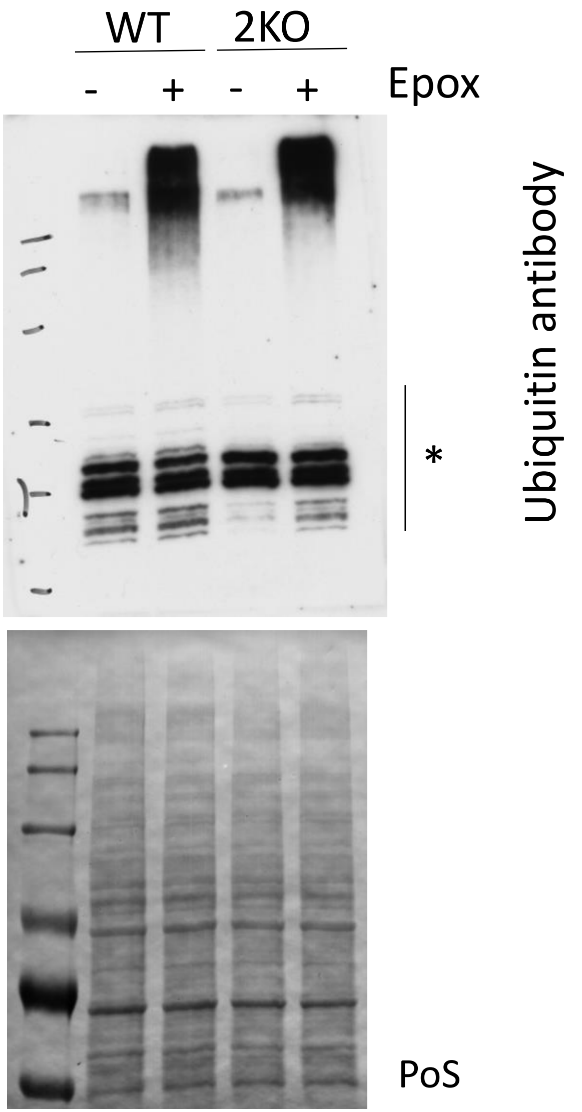

* previous decorations

Sup. 11c

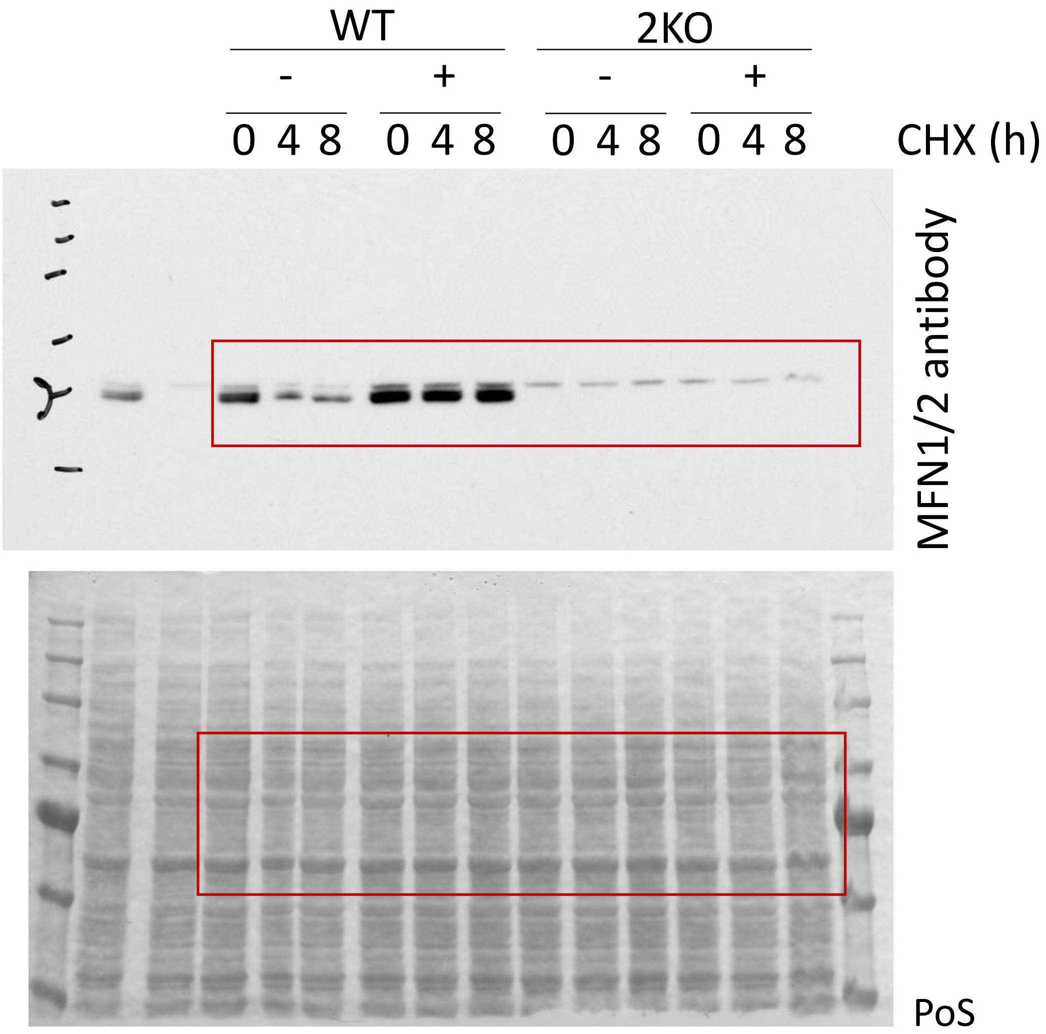

Sup. 11d

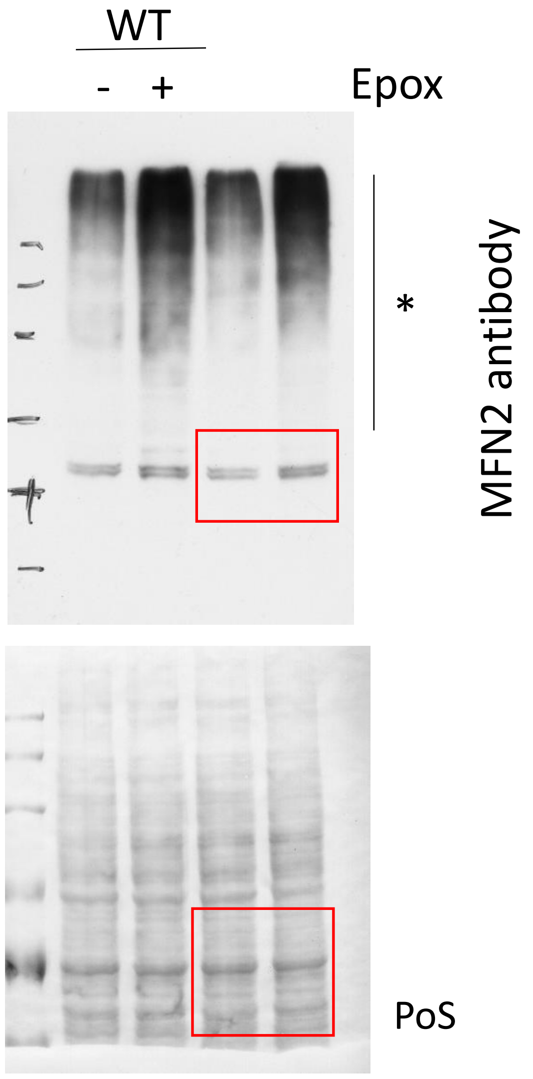

* previous decorations

Sup. 11e

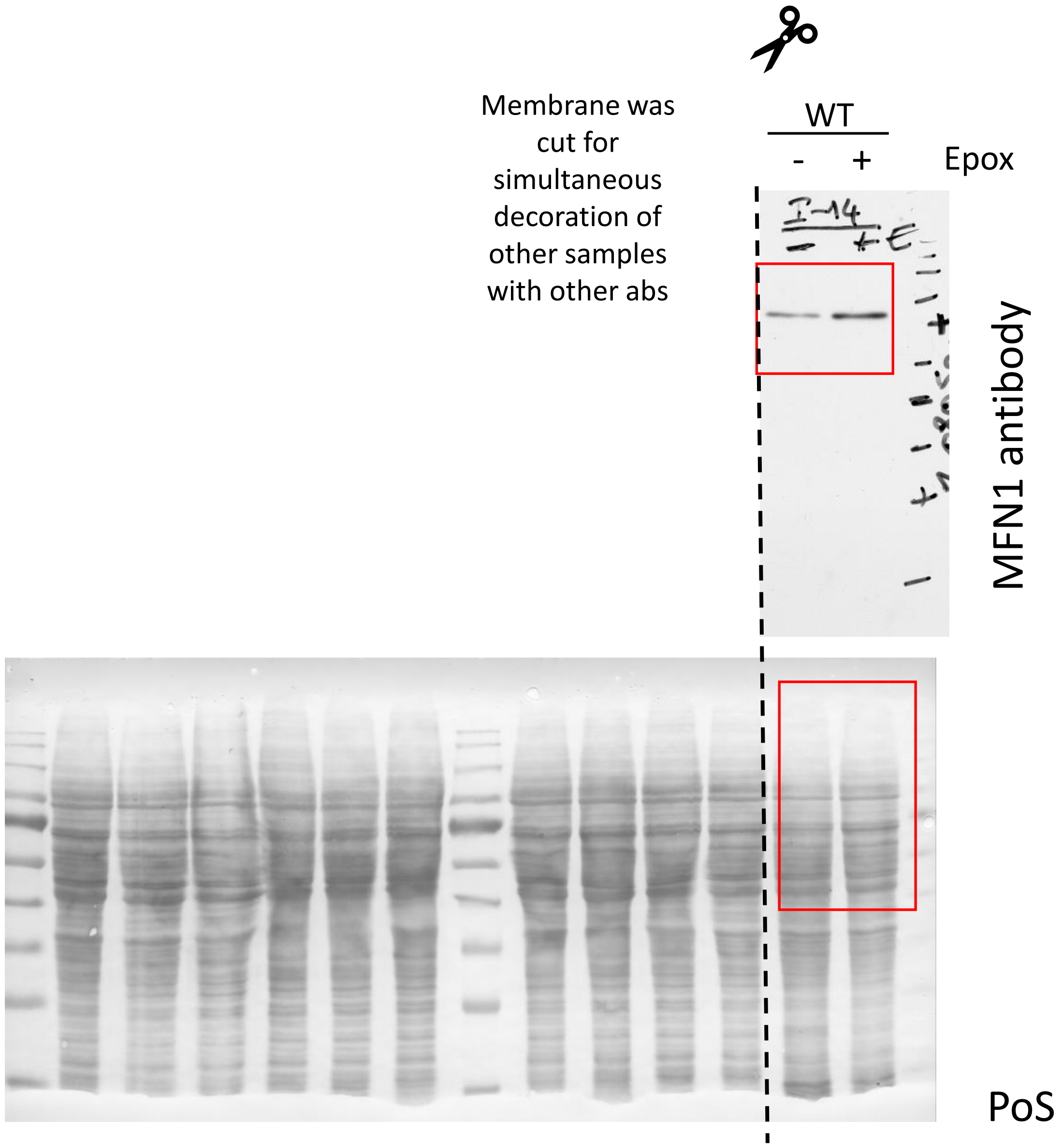
Membrane was cut for simultaneous decoration of other samples with other abs
WT
- +
Epox
MFN1 antibody
PoS

Sup. 11f

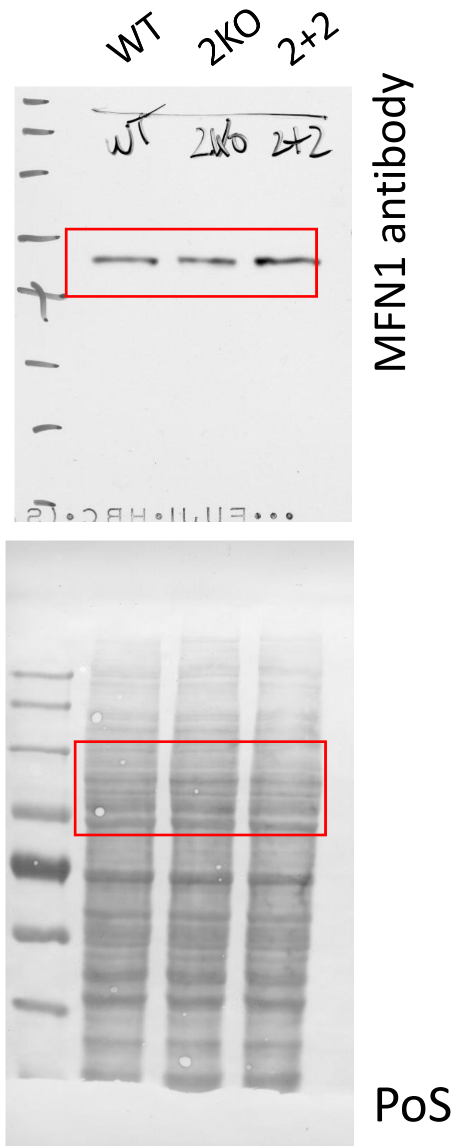

Sup. 12a

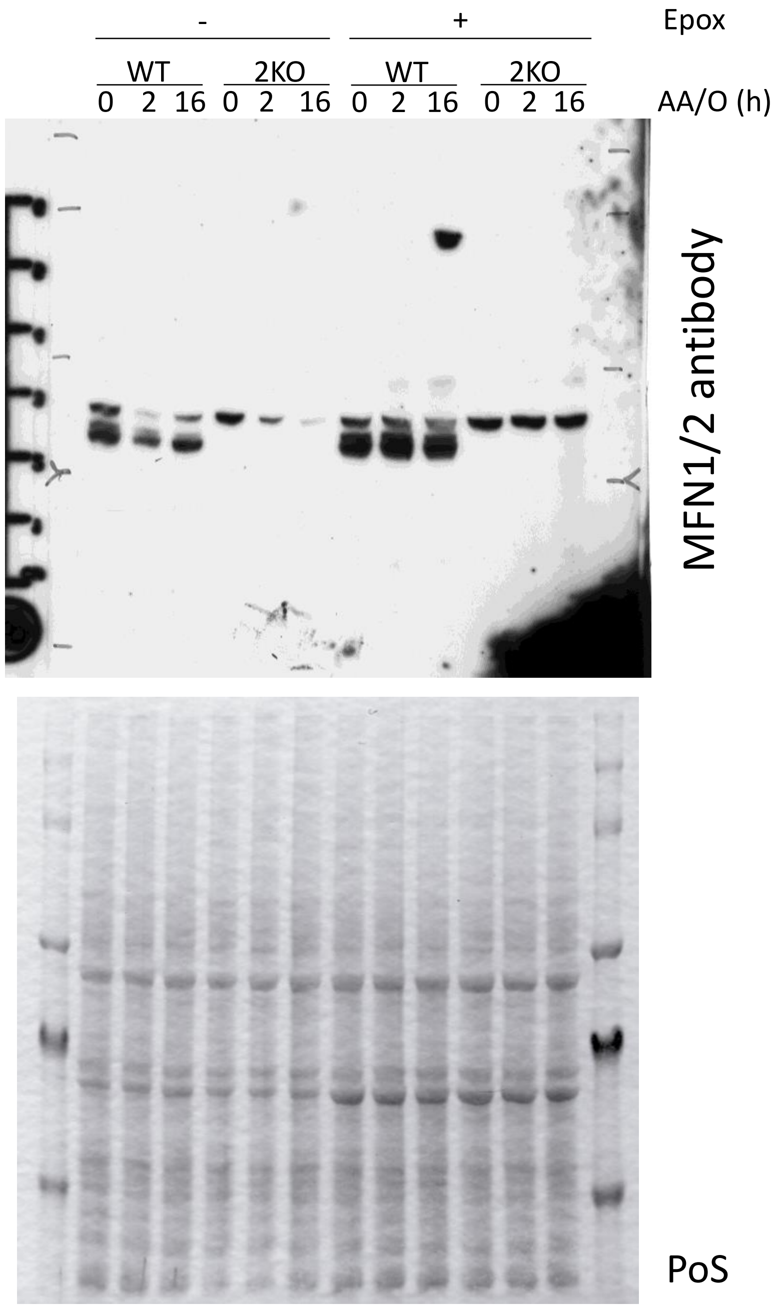

- | + | Epox
WT | 2KO | WT | 2KO
0 2 16 0 2 16 0 2 16 0 2 16 | AA/O (h)
MFN1/2 antibody
PoS

Sup. 12b

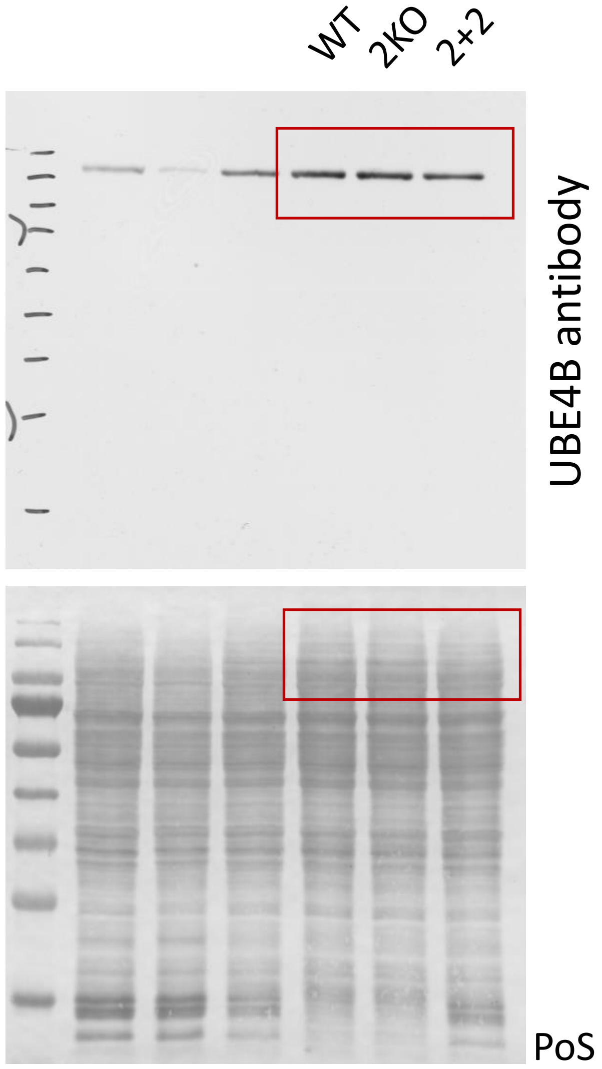

Sup. 12d

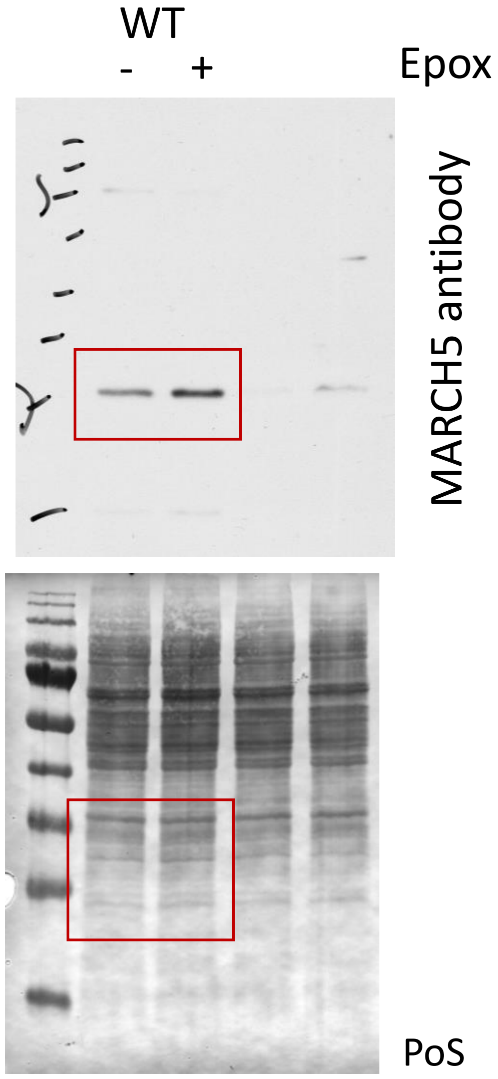

Sup. 12e

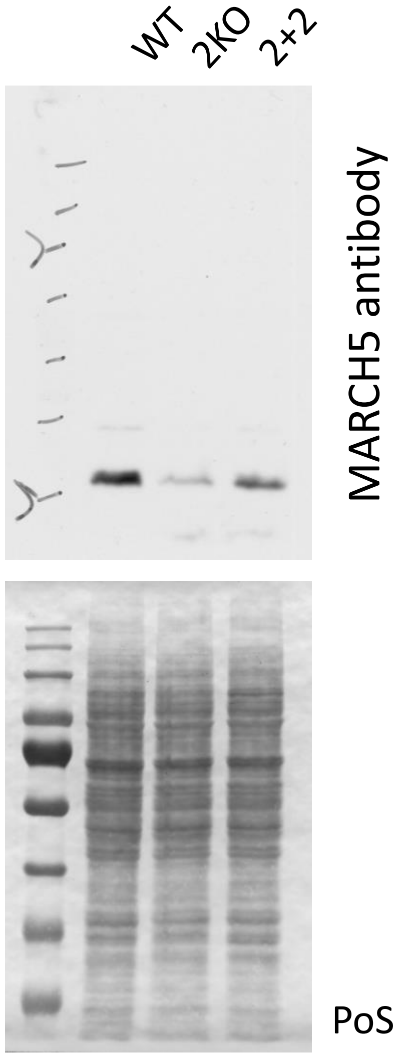

Sup. 13b

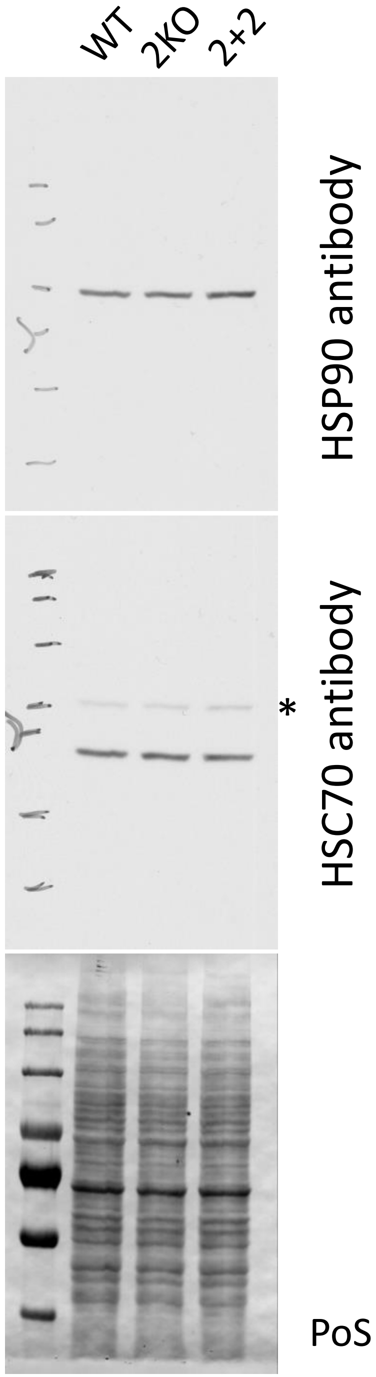

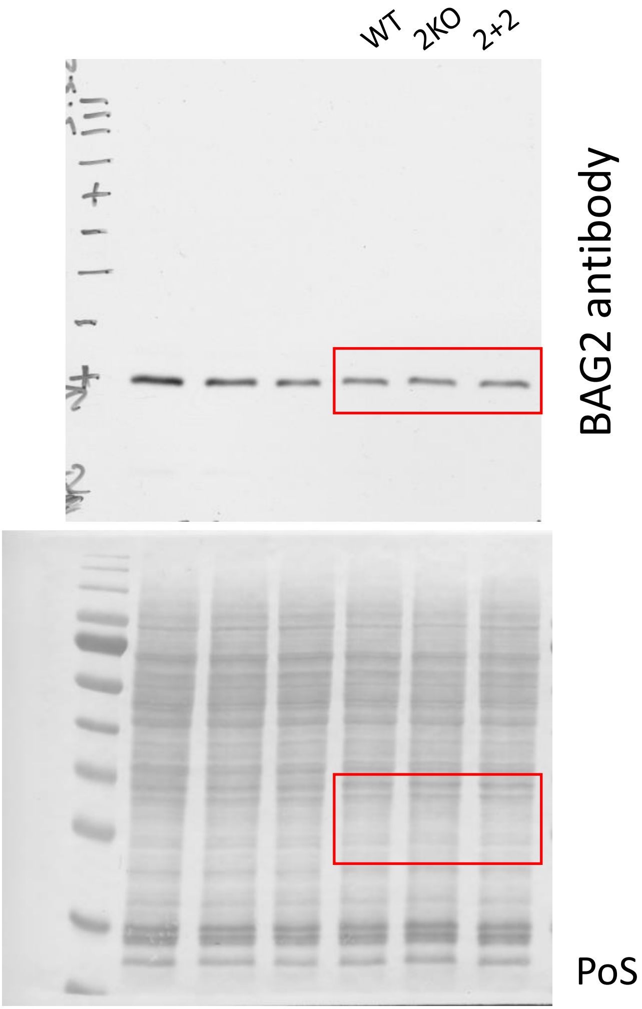

* previous decoration

Sup. 14a

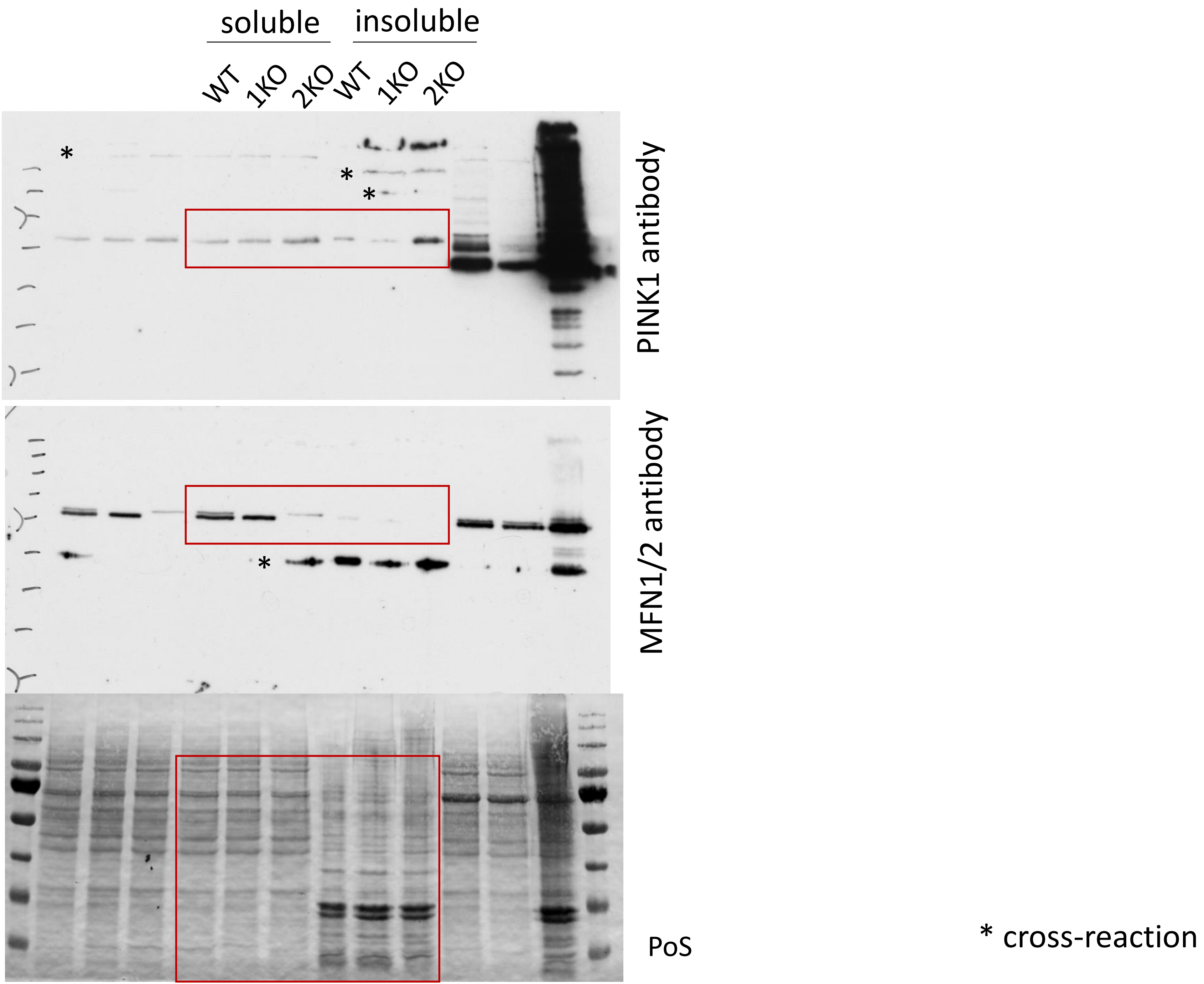
soluble
insoluble
WT
1KO
2KO
WT
1KO
2KO
*
*
*
PINK1 antibody
*
MFN1/2 antibody
PoS
* cross-reaction

Sup. 14d

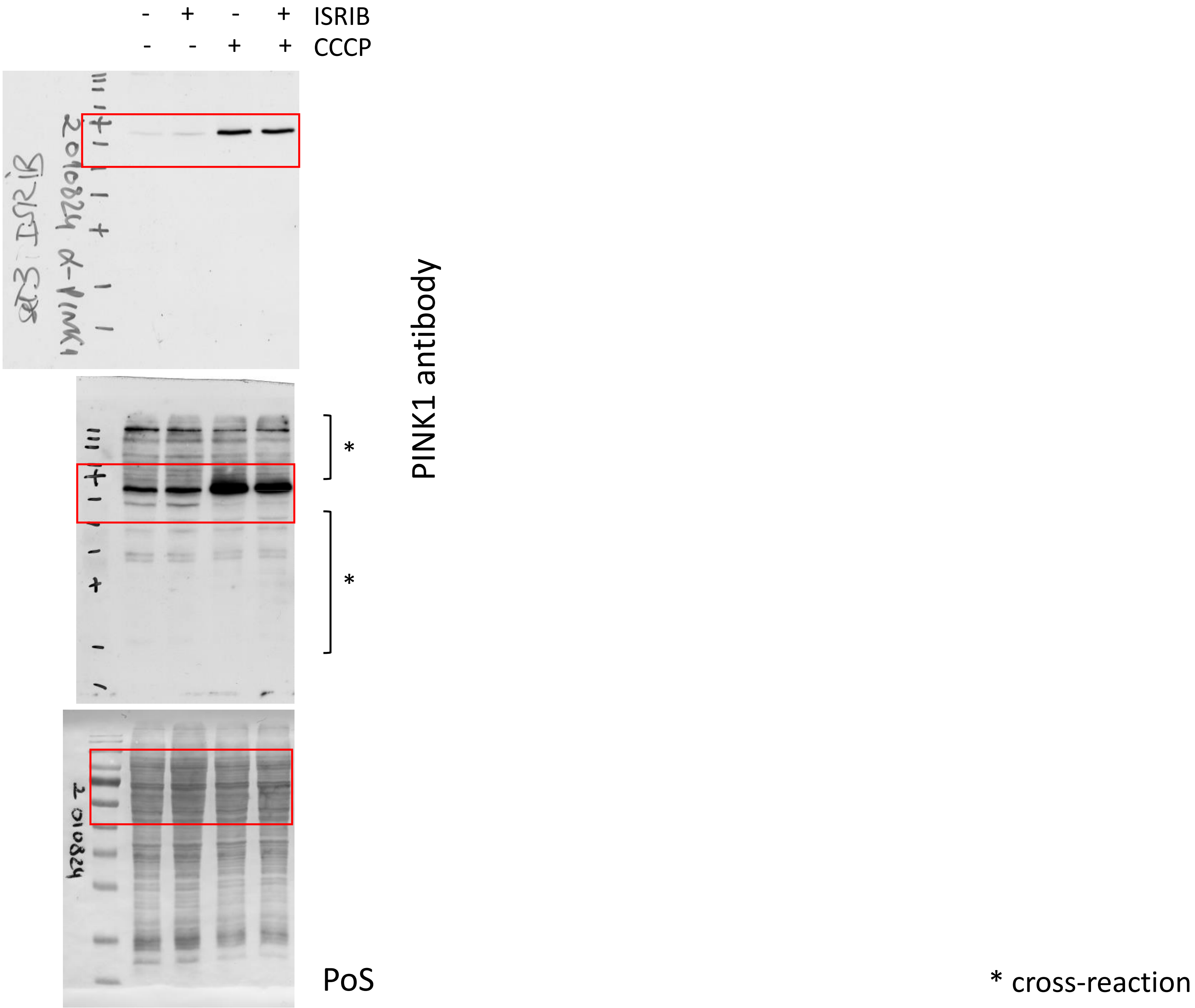

Sup. 14d

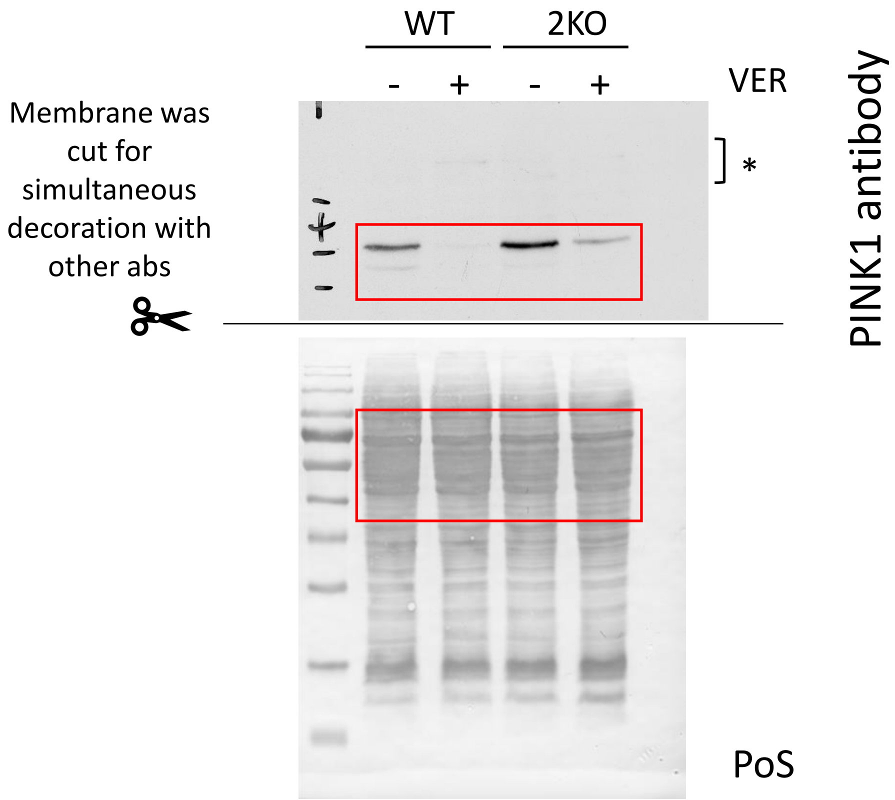

* cross-reaction

Sup. 16a

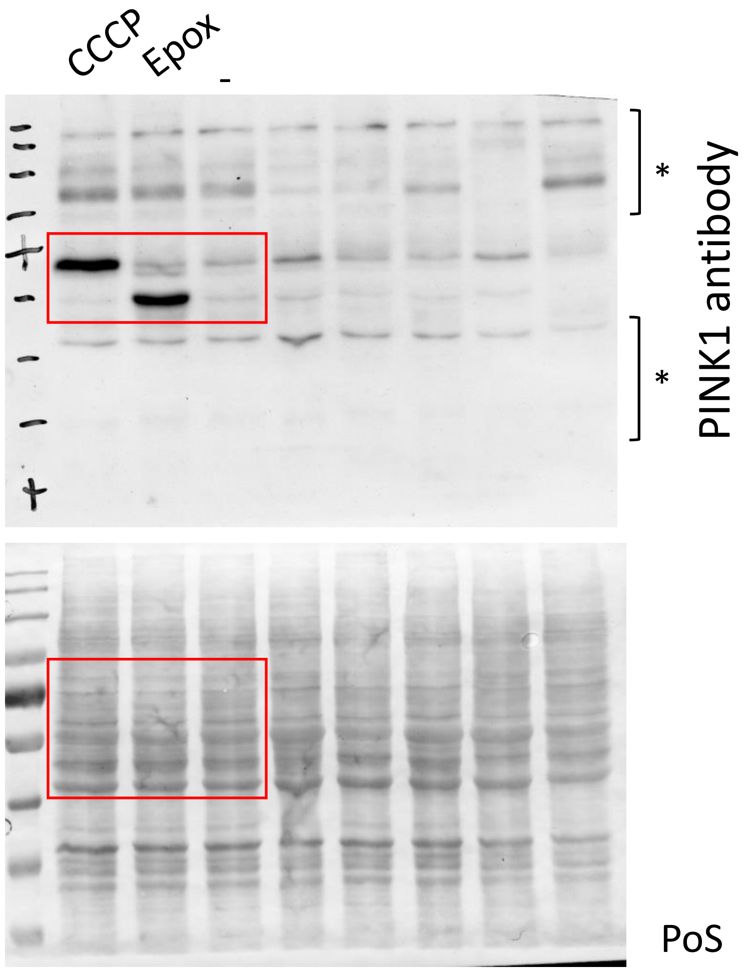

* cross-reaction

Sup. 16b

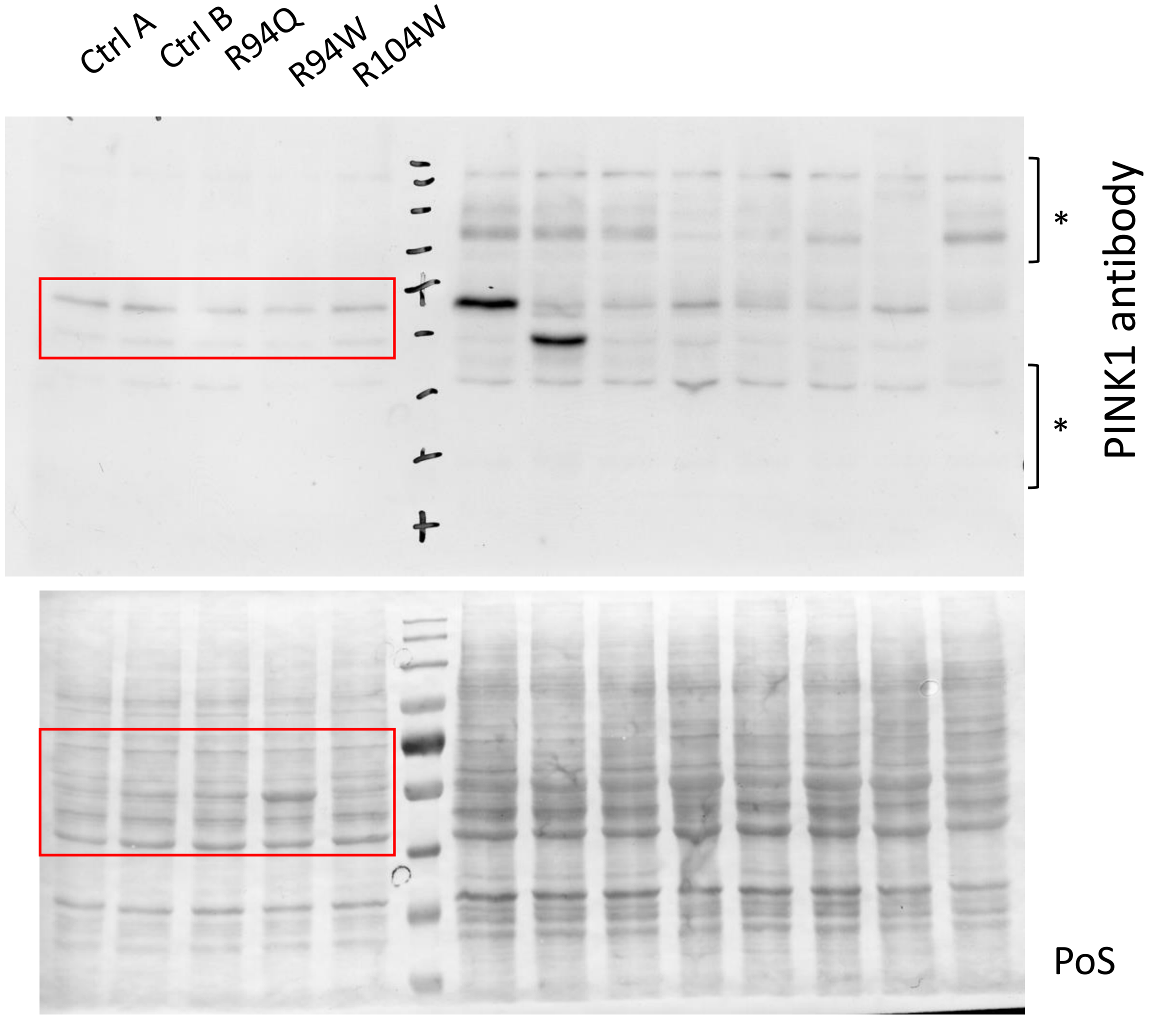

Supplement: Supplementary file 7 — Source data [file 41467_2025_56673_MOESM7_ESM.zip › Source data blots.pdf]
